# Testing the form-function paradigm: body shape correlates with kinematics but not energetics in selectively-bred birds

**DOI:** 10.1038/s42003-024-06592-w

**Published:** 2024-07-24

**Authors:** Samuel R. R. Cross, Andres C. Marmol-Guijarro, Karl T. Bates, John C. Marrin, Peter G. Tickle, Kayleigh A. Rose, Jonathan R. Codd

**Affiliations:** 1 Department of Musculoskeletal & Ageing Science, Institute of Life Course & Medical Sciences, University of Liverpool, William Henry Duncan Building, 6 West Derby Street, Liverpool, L7 8TX UK; 2 Faculty of Biology, Medicine & Health, University of Manchester, Manchester, UK; 3 German Centre for Integrative Biodiversity Research (iDiv) Halle-Jena-Leipzig, Puschstrasse 4, 04103 Leipzig, Germany; 4 Institute of Biology, Martin Luther University Halle-Wittenberg, Am Kirchtor 1, 06108 Halle, Germany; 5 3Diversity, Quito, Ecuador; 6 School of Biomedical Sciences, Faculty of Biological Sciences, University of Leeds, Leeds, UK; 7 Department of Biosciences, College of Science, Swansea University, Swansea, Wales UK

**Keywords:** Biomechanics, Animal physiology, Palaeontology, Modularity, Computer modelling

## Abstract

A central concept of evolutionary biology, supported by broad scale allometric analyses, asserts that changing morphology should induce downstream changes in locomotor kinematics and energetics, and by inference selective fitness. However, if these mechanistic relationships exist at local intraspecific scales, where they could provide substrate for fundamental microevolutionary processes, is unknown. Here, analyses of selectively-bred duck breeds demonstrate that distinct body shapes incur kinematic shifts during walking, but these do not translate into differences in energetics. A combination of modular relationships between anatomical regions, and a trade-off between limb flexion and trunk pitching, are shown to homogenise potential functional differences between the breeds, accounting for this discrepancy between form and function. This complex interplay between morphology, motion and physiology indicates that understanding evolutionary links between the avian body plan and locomotor diversity requires studying locomotion as an integrated whole and not key anatomical innovations in isolation.

## Abstract (web summary)

Duck breeds selectively bred for disparate body shapes are found to walk with different limb kinematics but incur similar energetic costs. This is attributed to the interplay of dynamic kinematic and modular anatomical processes.

## Introduction

Locomotion typically comprises a large part of an animal’s daily energy expenditure and therefore is critical to much of its behavioural ecology^1^. The biomechanics underpinning the acceleration of jointed body segments, alongside the physiological processes of energy generation by skeletal muscle, predicts that locomotor costs may be reduced through the possession of certain anatomical traits and the use of specific movement patterns^2–8^. Indeed, this has led many to infer that the evolution of locomotor morphology, mechanics and energetics are often causatively linked^9–12^. For example, the trunk and limb proportions, relatively muscular lower limbs and stiff plantigrade foot of humans have been mechanistically linked to lower energy costs and greater endurance in an upright bipedal gait when compared to the more energetically costly arboreally adapted morphologies and flexed limb postures of non-human apes^10,13–15^. In addition, a more general example can be found in terms of the hypothesised influence of body size upon causal relationships between morphology, kinematics and locomotor efficiency. Here, larger terrestrial animals tend to evolve so-called ‘graviportal’ limb morphologies^8,16,17^ and utilise more upright postures^4,18^, which may reduce external demands on both the skeleton and muscles and potentially increase pendular energy saving, yielding reduced locomotor costs^19,20^.

Birds are extensively used as a model system for studies of terrestrial locomotion^5–7,21–35^. Compared to other living bipedal clades, they exhibit unparalleled taxonomic, morphological and ecological diversity, and can therefore provide unique insight into mechanistic links and constraints between anatomical form and mechanical and physiological function. Birds stand and move with highly flexed limb postures, which are thought to have evolved from more upright postures in their dinosaurian ancestors^11,12,36,37^. The transition between these postural extremes has been tracked indirectly through fossil evidence of changes in limb proportions^37,38^, musculature^36,39–41^ and overall body shape^11,12,42^. Such use of morphological hallmarks or key innovations to infer evolutionary shifts in organismal function is commonplace in palaeontological studies^43–45^ and rests on the assumption that changes in form result in relatively straightforward or predictable changes to mechanics and/or energetics. However, despite this broad acceptance of the centrality of locomotor form-function to avian evolutionary history, relatively few studies^24,31,33–35,46^ of extant birds have quantified morphology, gait kinematics and locomotor cost simultaneously in a comparative context, to test directly for mechanistic links or interactions between form, function and physiology.

Here, we present such an analysis using three morphologically disparate breeds of Mallards (*Anas platyrhynchos*) as a case study system. Like all bipeds, the principle of static stability suggests that changes to body shape in ducks should necessitate concurrent postural changes that could impact upon locomotor energetics, while the high intraspecific disparity of domesticated ducks theoretically serves to minimise the influence of phylogenetic processes and specialisation. Our results demonstrate that selective breeding in Indian runner and Aylesbury ducks has resulted in considerable changes to the relative sizes of individual body segments, and ultimately, in gross body shape and mass distribution relative to wild Mallards. Differences in maximum performance and locomotor kinematics appear to correlate mechanistically with this morphological variation, supporting the idea that morphology and gait mechanics are coupled in response to modular changes in body proportions. However, remarkably, we find that this significant morpho-functional disparity does not result in any differences in locomotor energetics between the three breeds. We suggest therefore, that a complex interplay of factors, ranging from modular changes to morphology, and dynamic interactions between trunk and limb segments, may cancel out potential physiological gains and losses between the breeds during terrestrial locomotion, accounting for their energetic similarity. Our results stress the importance of caution when attempting to evaluate locomotor economy across evolutionary distances, as substantial changes to morphology may not necessarily correspond to changes in traditional measures of organismal performance or fitness. Furthermore, they emphasise the importance of viewing the locomotor system as a multi-element or modular complex, in which key evolutionary innovations or differences between taxa in specific anatomical characteristics should not be viewed in isolation.

## Results

### Morphology

The three duck breeds varied substantially in body size across most metrics analysed. Average whole-body mass was largest for Aylesbury ducks (2.28 ± 0.12 kg), followed by Indian runners (1.72 ± 0.05 kg), and Mallards (1.04 ± 0.05 kg). These absolute differences in mass translated to comparable differences in average skin-level segment volumes (Aylesbury, 0.0025 m^3^; Indian runner, 0.0019 m^3^; and Mallard, 0.0011 m^3^), but not average minimum skeletal convex hull volumes, where Indian runners were found to be slightly larger than Aylesburys (Indian runner, 0.0015 m^3^; Aylesbury, 0.0014 m^3^; and Mallard, 0.0007 m^3^). Differences in absolute body size were not always reflected in the absolute size of individual body segments. However, as this study will focus on normalised segment values, we provide a more thorough description of absolute segment sizes in Supplementary Notes 1.

**Fig. 1:**
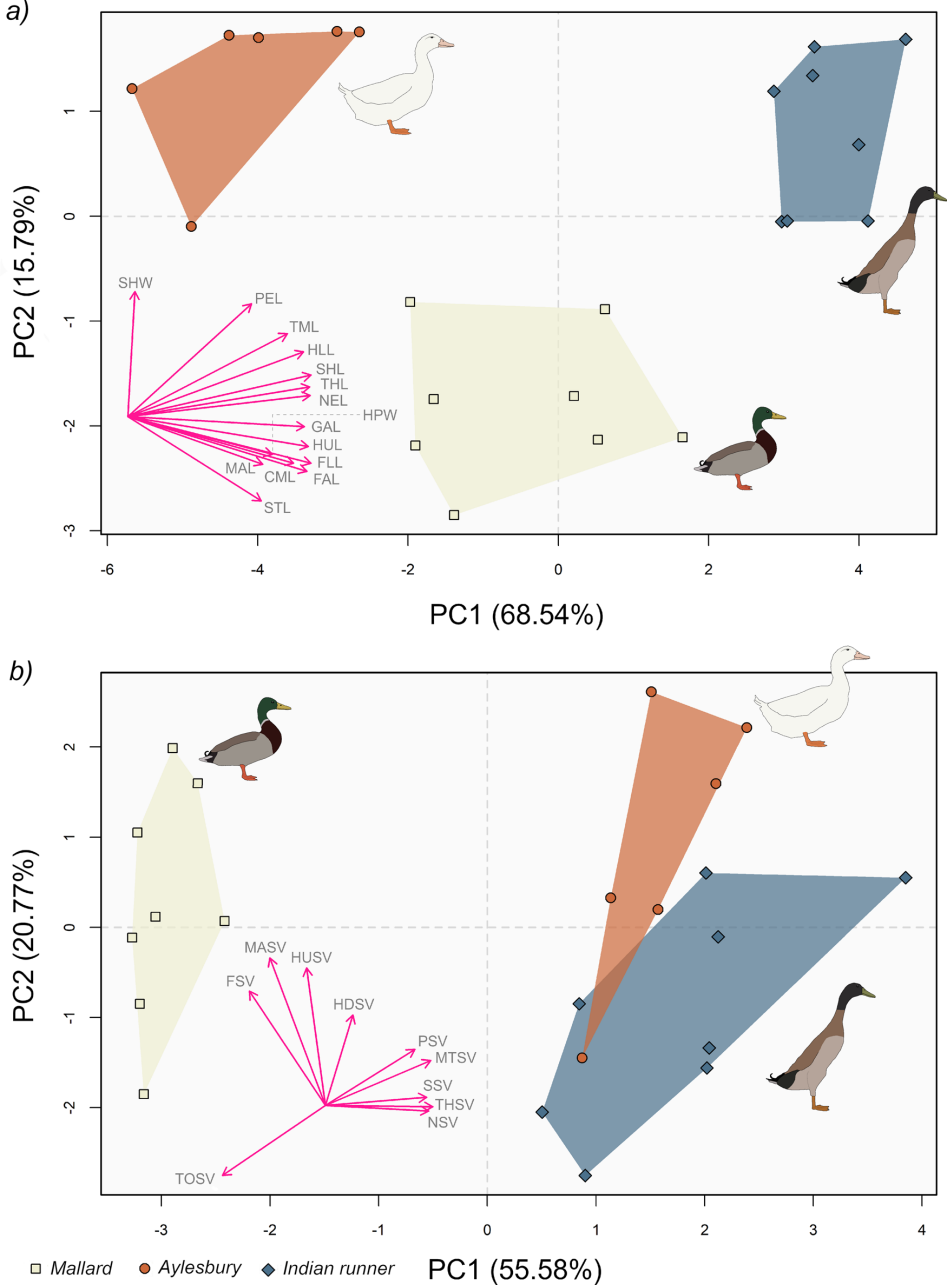
Principal component analysis of the duck breeds’ skeletal and soft tissue parameters. Linear skeletal measurements are presented in (**a**), and final skin segment volumes in (**b**), where each point represents a distinct individual. The biplots in both panels represent variable correlations with each principal component, note that these have been offset from 0,0 and increased in size (doubled), for legibility. Variable abbreviations are as follows, for **a**: THL thigh length, SHL shank length, TML tarsometatarsal length, PEL pes length, HLL total hindlimb length, HUL humeral length, FAL forearm length, CML carpometacarpal length, MAL manus length, FLL total forelimb length, NEL neck length, SHW shoulder width, HPW hip width, GAL gleno-acetabular length, STL sternum length. For **b**: HDSV head volume, NSV neck volume, TOSV torso volume, HUSV humeral volume, FSV forearm volume, MASV manus volume, THSV thigh volume, SSV shank volume, MTSV tarsometatarsal volume, PSV pes volume.

Principal component analysis (PCA) reveals strong divergence in body proportions between the three breeds, with Mallards, Aylesburys and Indian runners regularly occupying different areas of multivariate morphospace (see also Supplementary Notes 2 for additional statistical analyses). PCA of 14 linear skeletal measurements (Fig. 1a), found most parameters show a strong positive correlation with PC1 (>70%), though pes length and shoulder width were more strongly correlated with PC2 (>70%). Correlations between other parameters and PC2 were low to moderate (0% to ~50%), though a general trend of hindlimb parameters positively correlating, and forelimb and non-appendicular parameters (excluding shoulder width and neck length) negatively correlating was observed. Each breed occupies a discrete region of morphospace, where Aylesburys have the lowest PC1 scores, while Mallards then Indian runners have successively higher scores. This distribution reflects a gradient of increasing relative limb segment lengths along PC1, alongside increasing relative neck length, sternum length, and gleno-acetabular distance (Fig. 1a, Supplementary Fig. 7). This result aligns with our statistical analysis (Supplementary Table 6), which found Indian runners had significantly longer limbs and necks than the other breeds, and that Mallards had significantly longer forelimbs than Aylesburys. Both Mallards and Indian runners also have a significantly longer sternum and gleno-acetabular distance than Aylesburys, further contributing to their position on PC1. The positive distribution of the domestic breeds on PC2 is related primarily to their wide shoulders, which was found to be significantly greater than Mallards (Supplementary Table 8). Narrower shoulders and a relatively elongate forelimb, accounts for the negative distribution of Mallards along PC2 (Fig. 1a, Supplementary Fig. 7). Additional morphospace analyses were performed at a regional level (forelimb, hindlimb, non-appendicular), to investigate localised differences in the dataset. Those results corroborate Fig. 1a, finding that breeds generally segregate from one another (a full overview is provided in Supplementary Notes 3).

PCA of 11 skin segment volumes (Fig. 1b) found PC1 to strongly correlate (>75%) with hindlimb segment and neck volumes, though torso (−91%), forearm (−67%), and manus (−49%) volume showed strong-moderate negative correlations. PC2 was positively correlated with forelimb (61–79%) and head (49%) volumes, while torso volume was the primary negative correlate (−38%). Mallards scored negatively on PC1, reflecting their proportionately smaller necks and hindlimbs, and a relatively large torso and forelimb compared to Aylesbury ducks and Indian runners (Supplementary Fig. 9). This result is supported by our statistical analysis, which found significant differences between the breeds in these parameters (Supplementary Table 8). The domestic breeds primarily differentiate along PC2, with Aylesburys being more positively scored on average (Fig. 1b). This appears to be caused by significantly larger distal forelimb and pes volumes in that breed (Supplementary Fig. 9), though there is a slight overlap in space occupation. The distribution of breeds in Fig. 1b approximates that of the minimum skeletal convex hull volumes, which are presented in Supplementary Notes 3.

**Fig. 2:**
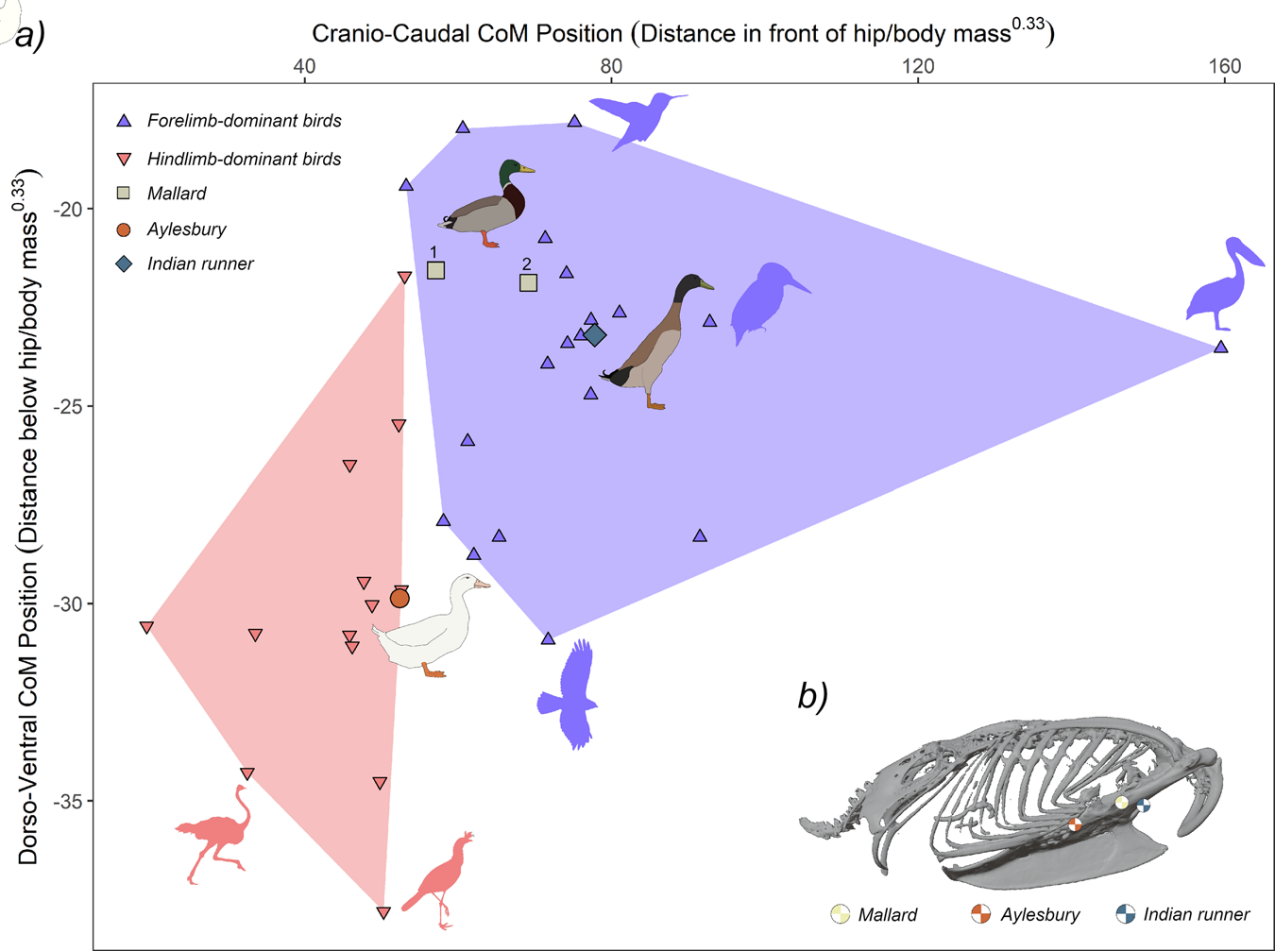
Comparison of centre-of-mass position between the duck breeds and other birds. Morphospace (**a**), illustrates craniocaudal and dorsoventral CoM position in the three duck breeds compared to the larger avian dataset of Macaulay et al.^12^, who found that extant birds group into hindlimb-dominant and forelimb-dominant morphospace zones based upon their primary locomotor habits. Note that the total number of ducks in this plot amounts to four, as Macaulay et al., included a mallard in their original dataset (designated 1, with the new mallard designated 2). CoM values (distance in front and below the hip) have been normalised to body mass^0.33^. A reference figure showing where normalised CoM for each breed would be located if positioned against the mallard torso is presented in (**b**). Duck illustrations by S.R.R.C. silhouettes of other birds are public domain from www.phylopic.org.

Consistent with the disparate occupation of body segment morphospace (Fig. 1), breeds are found to diverge substantially in overall body shape, as evidenced by the differing centre of mass (CoM) estimations (Fig. 2). When placed in the context of Macaulay et al.’s^12^ larger dataset of avian CoM, Mallards are located within the forelimb-dominant CoM space, and have a notably more dorsal CoM than the other breeds, but are cranio-caudally intermediate. A position amongst forelimb-dominant taxa is also found for the Indian runner, which has a CoM ventral and cranial to Mallards. In contrast, the Aylesbury sits within hindlimb dominant space, with a CoM slightly caudal to Mallards, but considerably more ventral than the other breeds (Fig. 2).

**Fig. 3:**
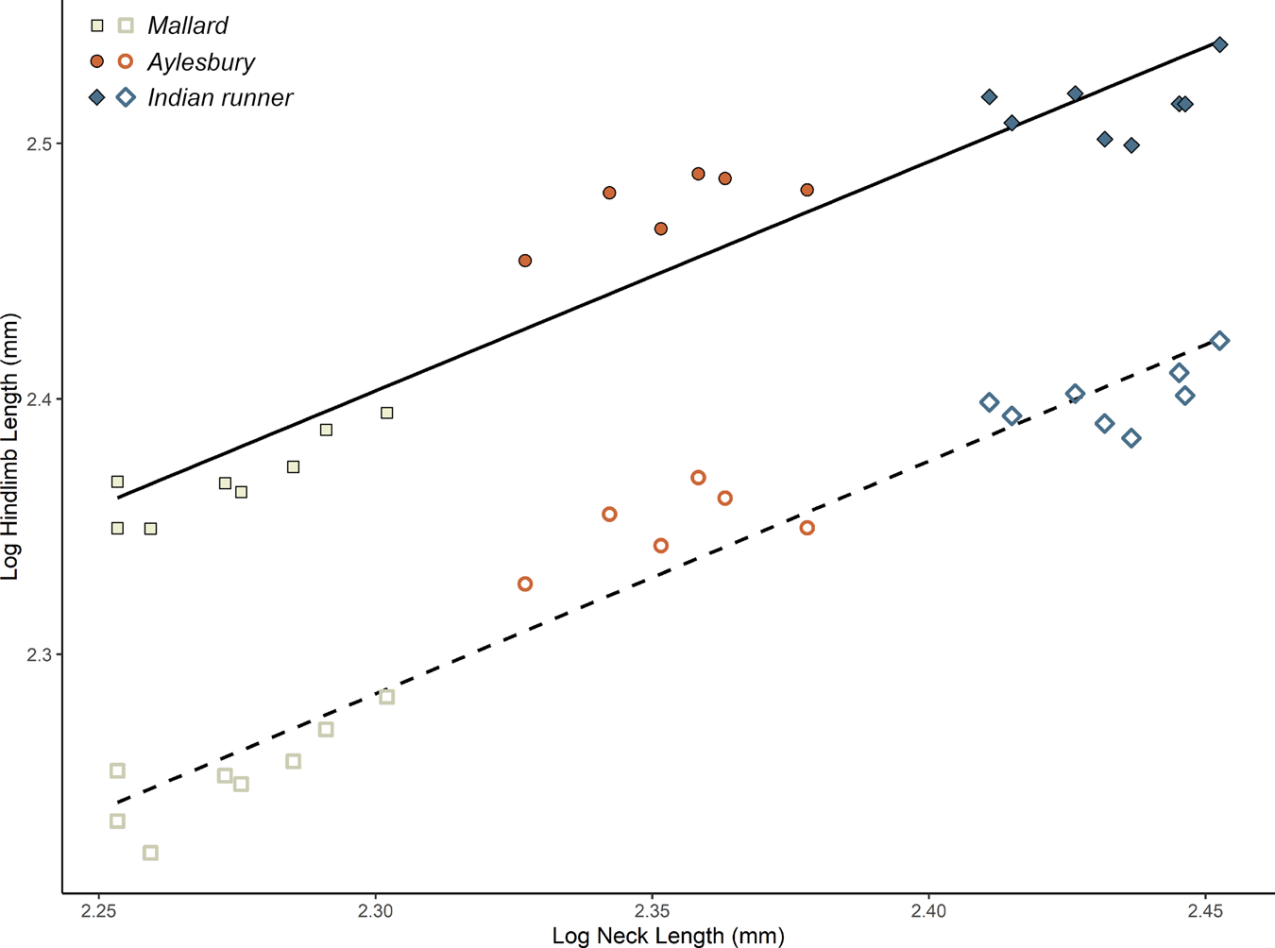
Integration of neck and hindlimb lengths in the duck breeds. Log-log plot of hindlimb and neck length in the three duck breeds, with trendlines estimated via ordinary least-squares regression. The upper (solid) line and filled points correspond to the analysis where hindlimb length was measured as its total length (i.e. the sum of the femur, tibiotarsus, tarsometatarsus, and pes), whereas the lower (dashed) line and unfilled points correspond to the analysis where functional hindlimb length was used instead (i.e. the pes is not included). Statistical information is quoted in the main text.

Specific inter-segmental relationships were investigated further with ordinary least-squares regression, which showed that raw neck length and hindlimb length (both total and functional lengths) were positively correlated across all ducks, with breeds showing distinct groupings according to their absolute sizes (Fig. 3). The relationship with total hindlimb length was significant (*p* = <0.001), and accounts for a substantial amount of total variance (adj. *R*^2^ = 0.9). Likewise, the correlation with functional hindlimb length was also significant (*p* < 0.001) and explains a similar proportion of variance (adj. *R*^2^ = 0.93).

### Energetics

All ducks walked on a treadmill at aerobically sustainable speeds, during which energetic data was collected. Maximum sustainable speed (*U*_max_) was highest in Indian runners (1.11 m s^−1^), intermediate in Aylesburys (0.83 m s^−1^) and lowest in Mallards (0.75 m s^−1^). After size-normalising speed, max ${\hat{U}}_{\max }$ became closer, particularly between Mallards (Fr = 0.56 ± 0.012) and Aylesburys (Fr = 0.56 ± 0.005). The Indian runners ${\hat{U}}_{\max }$ max, however, remained the fastest (Fr = 0.69 ± 0.008).

**Fig. 4:**
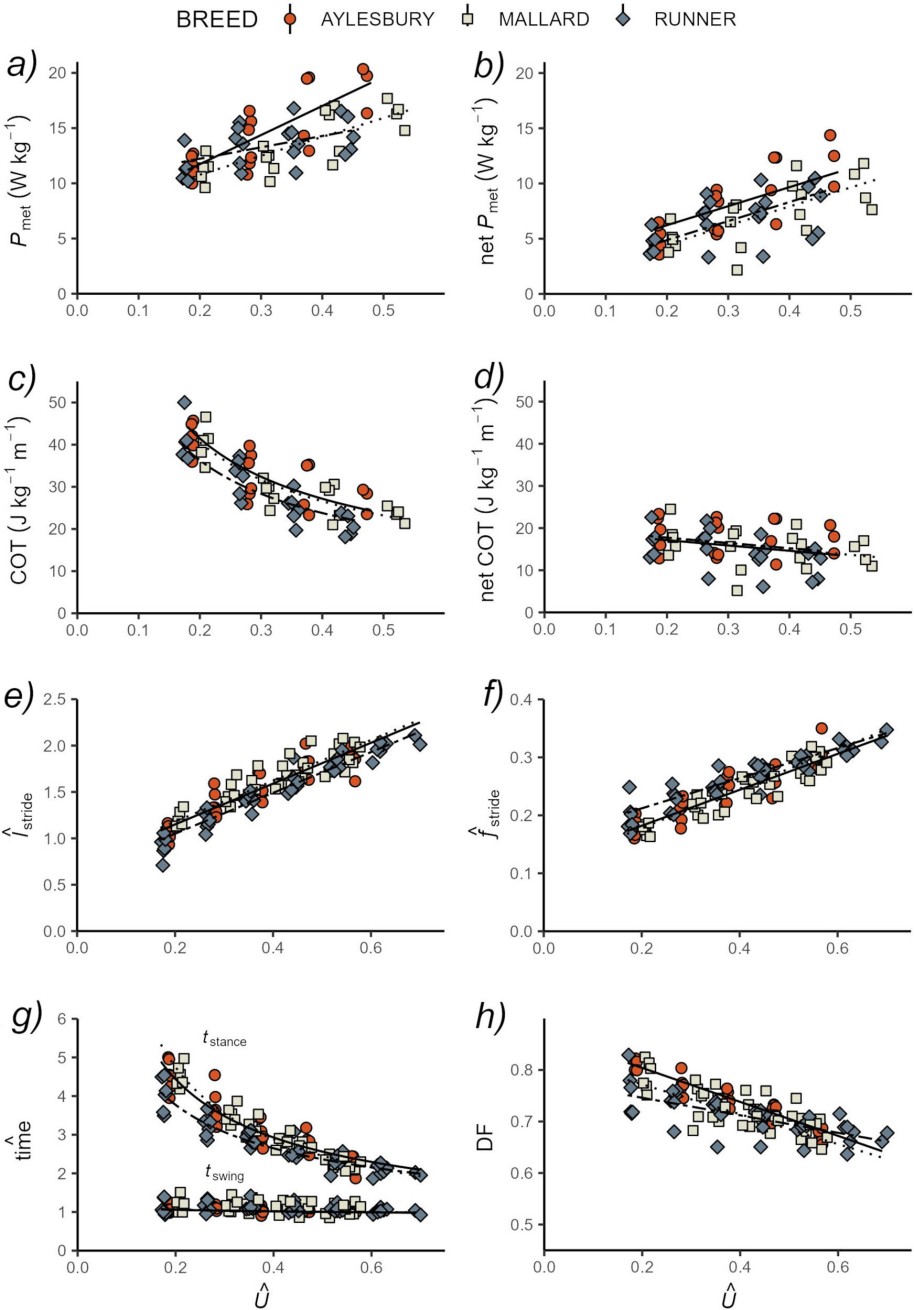
Body size-normalised energetics and spatiotemporal kinematics during treadmill locomotion. All energetics (**a**–**d**) and spatiotemporal kinematic (**e**–**h**) parameters are given for the range of *Û* and comprise data from five mallards (beige squares, dotted line), six Aylesbury ducks (red circles, solid line), and six Indian runners (blue diamonds, dot-dash line). **a** Metabolic power (${P}_{{{\rm{met}}}}$), **b** net metabolic power $({{\rm{net}}}{P}_{{{\rm{met}}}})$, **c** cost of transport (COT), **d** net cost of transport (net– COT), **e** stride length (${\hat{l}}_{{{\rm{stride}}}}$), **f** stride frequency $({\hat{f}}_{{{\rm{stride}}}})$, **g** stance $({\hat{t}}_{{{\rm{stance}}}})$ and swing $({\hat{t}}_{{{\rm{swing}}}})$ durations, and **h** duty factor (${{\rm {DF}}}$).

Some energetic differences were found between the breeds after speed was size-normalised, however, once the standing metabolic rate was accounted for, no significant differences in the cost of locomotion remained. ${{\rm{Mass}}}-{{\rm{specific}}}\; {{\rm{metabolic}}}\; {{\rm{power}}}({P}_{{{\rm{met}}}})$ increased linearly with $\hat{U}$ in all breeds, and Aylesbury ducks were found to have the highest values (Fig. 4a). ${{\rm{net}}}\mbox{-}{P}_{{{\rm{met}}}}$ also increased linearly with $\hat{U}$, while a similar pattern of offset in magnitude was observed (i.e. differing regression intercepts) between the breeds; Aylesburys incurred a metabolic rate ~94% and ~170% higher than Indian runners and Mallards, respectively (Fig. 4b; Table 1). Despite this, no differences in regression slope were detected after normalising for body size (Fig. 4b). Similarly, there was a general trend in all breeds for greater locomotor economy with increasing speed, and while differences between breeds were apparent in the magnitude of the cost of transport (CoT), fitted models shared a common slope. Indian runner CoT was ~11% and ~13% cheaper than mallards and Aylesbury ducks across all $\hat{U}$ (Fig. 4c; Table 1). However, after removing standing energetic costs, there was a significant decrease in net–CoT with $\hat{U}$ across all breeds, which shared a common slope and intercept (Fig. 4d; Table 1). Non-normalised energetics results are presented in Supplementary Notes 4.

**Table 1:** Model results for the size-normalised (relative speed; *Û*) energetic and spatiotemporal kinematic parameters

Parameter	Final model	*r*^2^	${{n}_{{\rm {p}}}}^{2}$	Equations
*Energetics*				
*P*_met_	$\hat{U}$ (*F*_1, 56_ = 2.89; *P* = 0.095) Breed (*F*_2, 56_ = 71.18; *P* < 0.001 **)	0.71	0.05 0.71	IR: $27.19\hat{U}+15.09$ MA: $27.19\hat{U}+$3.77 AL: $27.19\hat{U}+24.26$
Mass specific *P*_met_	$\hat{U}$ (*F*_1, 54_ = 51.69; *P* < 0.001 **) Breed (*F*_2, 54_ = 4.99; *P* = 0.010 *) $\hat{U}\times {{\rm{Breed}}}$ (*F*_2, 54_ = 3.65; *P* = 0.033*)	0.52	0.49 0.15 0.12	IR: $10.29\hat{U}+10.17$ MA: $17.11\hat{U}+7.36$ AL: $26.35\hat{U}+6.43$
Net *P*_met_	$\hat{U}$ (*F*_1, 56_ = 41.48; *P* < 0.001 **) Breed (*F*_2, 56_ = 3.76; *P* = 0.029 *)	0.44	0.43 0.12	IR: $17.21\hat{U}+1.43$ MA: $17.21\hat{U}+1.02$ AL: $17.21\hat{U}+2.75$
log_10_ COT	${\log }_{10}\hat{U}$ (*F*_1, 56_ = 137.82; *P* < 0.001 **) Breed (*F*_2, 56_ = 5.81; *P* = 0.005 *)	0.71	0.71 0.17	IR: $13.63{\hat{U}}^{-0.61}$ MA: $15.23{\hat{U}}^{-0.61}$ AL: $15.61{\hat{U}}^{-0.61}$
net COT	$\hat{U}$ (*F*_1, 58_ = 5.70; *P* < 0.016 **)	0.07	–	IR: $-12.59\hat{U}+19.95$ MA: $-12.59\hat{U}+19.95$ AL: $-12.59\hat{U}+19.95$
*Spatiotemporal kinematics*				
${\hat{l}}_{{{\rm{stride}}}}$	$\hat{U}$ (*F*_1, 105_ = 712.270; *P* < 0.001 **) Breed (*F*_2, 105_ = 13.532; *P* < 0.001 **)	0.87	0.87 0.20	IR: $2.20\hat{U}+0.61$ MA: $2.20\hat{U}+$0.74 AL: $2.20\hat{U}+0.70$
${\hat{f}}_{{{\rm{stride}}}}$	$\hat{U}$ (*F*_1, 103_ = 542.28; *P* < 0.001 **) Breed (*F*_2, 103_ = 9.63; *P* < 0.001 **) $\hat{U}\times {{\rm{Breed}}}$ (*F*_2, 103_ = 3.54; *P* = 0.03 *)	0.83	0.83 0.15 0.06	IR: $0.26\hat{U}+0.16$ MA: $0.34\hat{U}+0.11$ AL: $0.31\hat{U}+0.12$
${\log }_{10}\,{\hat{t}}_{{{\rm{stance}}}}$	${\log }_{10}\hat{U}$ (*F*_1, 103_ = 871.30; *P* < 0.001 **) ${{\rm{Variety}}}$ (*F*_2, 103_ = 20.21; *P* < 0.001 **) ${\log }_{10}\hat{U}\times {{\rm{Breed}}}$ (*F*_2, 103_ = 8.90; *P* < 0.001 **)	0.89	0.89 0.26 0.14	IR: $1.66{\hat{U}}^{-0.51}$ MA: $1.49{\hat{U}}^{-0.72}$ AL: $1.69{\hat{U}}^{-0.60}$
${\log }_{10}\,{\hat{t}}_{{{\rm{swing}}}}$	${\log }_{10}\hat{U}$ (*F*_1, 107_ = 4.09; *P* < 0.001 **)	0.03	–	IR: $0.96{\hat{U}}^{-0.06}$ MA: $0.96{\hat{U}}^{-0.06}$ AL: $0.96{\hat{U}}^{-0.06}$
DF	$\hat{U}$ (*F*_1, 103_ = 200.17; *P* < 0.001 **) Breed (*F*_2, 103_ = 8.87; *P* < 0.001 **) $\hat{U}\times {{\rm{Breed}}}$ (*F*_2, 103_ = 7.35; *P* = 0.002 *)	0.68	0.66 0.14 0.12	IR: $-0.17\hat{U}+0.78$ MA: $-0.29\hat{U}+0.84$ AL: $-0.33\hat{U}+0.87$

This comprises ANCOVA results for each studied parameter, identifying breed-specific differences across the studied speed range.

The minimum CoT of each duck breed was found to sit within the 95% confidence intervals of a linear regression of body mass and minimum CoT in 11 avian taxa, suggesting that these ducks are not outliers amongst birds, expending comparable energy for their mass during walking, thereby avoiding the elevated CoTs found in other domesticated poultry (for example broiler chickens^47^; Supplementary Notes 5). When all avian taxa were analysed together (i.e. the ducks were included), this yielded a new bird-specific minimum CoT allometric equation (*y* = 1.392*x*^−0.454^), as well as a phylogenetically corrected variant (*y* = 1.368*x*^−0.441^).

### Kinematics

Spatiotemporal kinematic data was collected across the same speed range as the energetics, and once normalised, showed the breeds generally used diverging kinematic strategies. A positive linear trend was found between relative stride length (${\hat{l}}_{{{\rm{stride}}}}$) and $\hat{U}$, and the rate of change with *U* was similar across the three breeds (Fig. 4e; Table 1). Mallards had the largest ${\hat{l}}_{{{\rm{stride}}}}$ across $\hat{U}$, whilst the Indian runners had the smallest (Fig. 4e; Table 1). The change in relative stride frequency $({\hat{f}}_{{{\rm{stride}}}})$ with increasing $\hat{U}$ was different between breeds (Fig. 4f; Table 1); the ${\hat{f}}_{{{\rm{stride}}}}$ slope of the Indian runner ducks was the highest, and the intercept the lowest, of the breeds although ${\hat{f}}_{{{\rm{stride}}}}$ converged at the maximum $\hat{U}$. For relative stance time (${\hat{t}}_{{{\rm{stance}}}}$), the Indian runners had a shorter support phase across the majority of $\hat{U}$, decreasing at a slower rate compared to the other breeds but converging at maximum $\hat{U}$ (Fig. 4g; Table 1). A negative curvilinear trend was detected between relative swing time (${\hat{t}}_{{{\rm{swing}}}}$) and $\hat{U}$, with a common slope and intercept for all breeds (Fig. 4g; Table 1). Duty factor (${DF}$) decreased linearly with $\hat{U}$, but none of the breeds shared a common slope or intercept (Fig. 4h; Table 1). *DF* decreased fastest in Aylesbury ducks and slowest in Indian runners. Non-normalised spatiotemporal kinematics results are presented in Supplementary Notes 4.

**Fig. 5:**
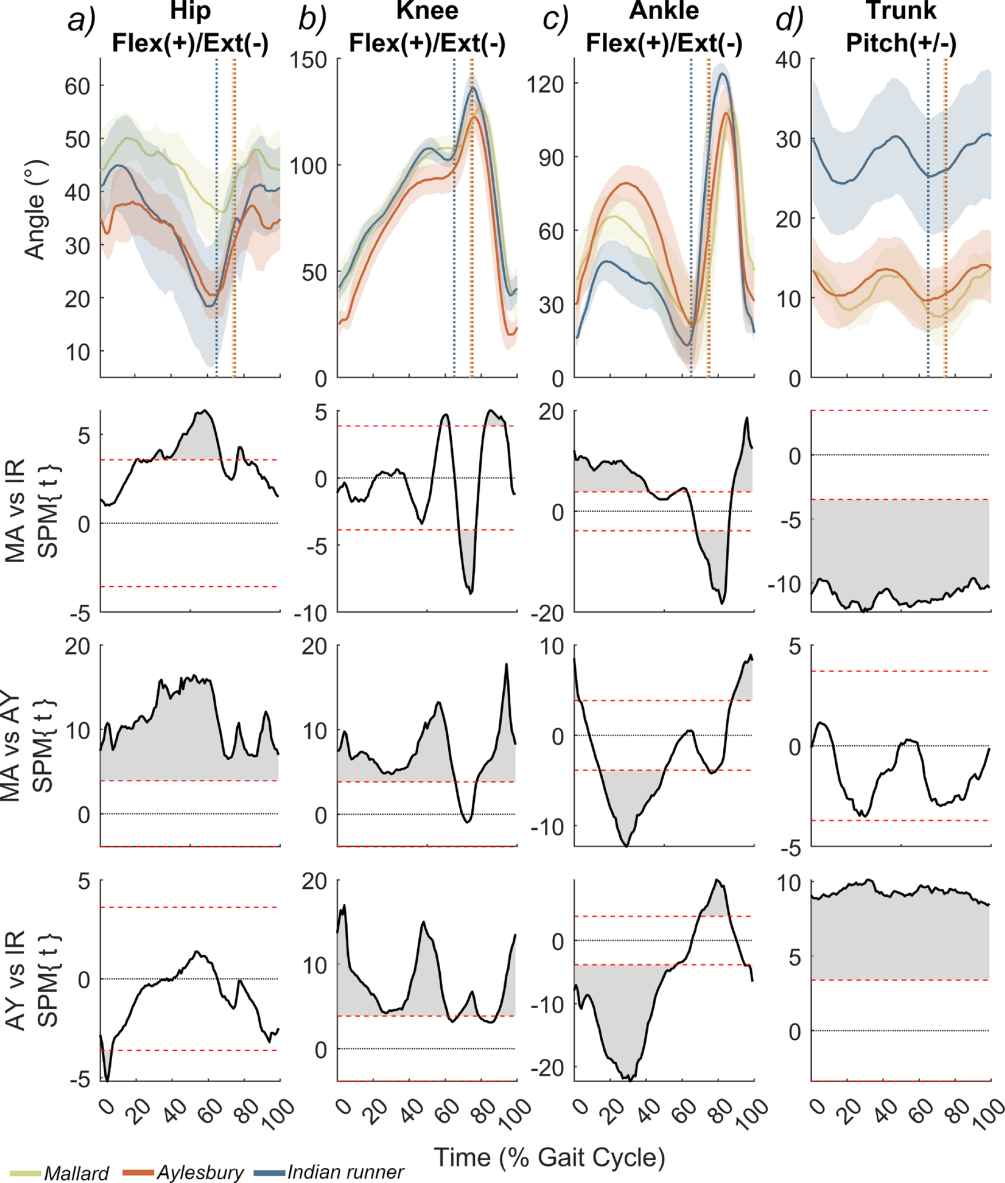
Comparison of hindlimb flexion–extension and trunk pitch angles between the duck breeds. Angular traces are shown for (**a**) the hip, (**b**) the knee, (**c**) the ankle, and (**d**) the trunk, with corresponding statistic parametric mapping (SPM) results presented in the column beneath. For all plots, the *X*-axis represents the duration of one stride, such that 0 indicates the start of stance (touch down), and the dotted lines delimit the average breed-specific start of the swing phase (toe-off). The angular traces are presented as a mean and standard deviation and have been transformed so that flexion is always positive. The SPM subplots illustrate where in the stride cycle the breeds significantly differ from one another; the region bounded by the red dashed lines indicates non-significance, and therefore wherever this area is exceeded (in grey) a significant difference occurs. The data presented here consists of 30 strides per breed, sourced from three mallards (10 continuous strides each), 2 Aylesbury ducks (15 continuous strides each), and 3 Indian runners (10 continuous strides each). The raw trace data is plotted in Supplementary Fig. 29.

Breeds were also found to utilise significantly different joint kinematics, both at specific points in the stride (Supplementary Notes 6), and across the entire gait cycle more generally (Fig. 5a–c). Aylesbury ducks were found to be more extended at the hip and knee than mallards during stance, but more flexed at the ankle (Fig. 5a–c). Indian runners underwent large excursions in hip angle, beginning stance with hip flexion intermediate between the other breeds, but most extended at terminal stance (Fig. 5a). In addition, Indian runners were found to use similar knee kinematics to mallards (Fig. 5b), but tended to operate with the most extended ankle (Fig. 5c). Mallards and Aylesburys were not found to significantly differ in terms of trunk pitch; both maintained a fairly pronograde posture, with a relatively pronounced two-hump profile. Conversely, the Indian runner tended to use a more orthograde posture, with a similar humped profile but considerably higher variance (Fig. 5d). Comparison of CoM against pes position showed that our models matched the expected positional relationships of these parameters across the gait cycle, with the foot located beneath the CoM around midstance (Supplementary Notes 7).

**Fig. 6:**
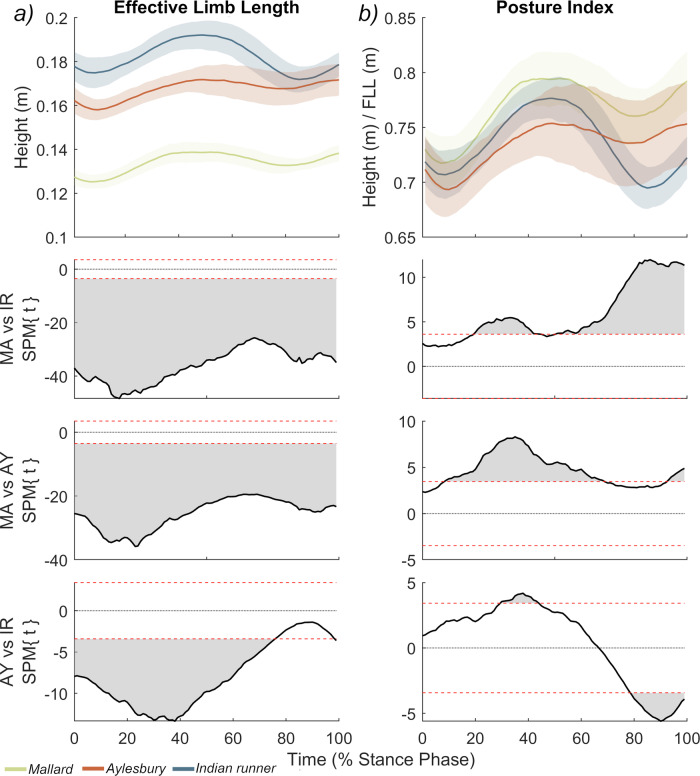
Effective limb length and postural index across the stance phase. The absolute hip height, referred to as ELL, is presented in **a**, while the postural index (ELL/FLL) is presented in (**b**). Hip height was derived from the same kinematic simulations used to acquire joint angles (Fig. 5), and these results are directly comparable to the stance phase kinematics presented in that figure. In addition, for instructions concerning interpretation of the subplots as well as sample size information, please also refer to Fig. 5. The raw trace data is plotted in Supplementary Fig. 30.

Differences in posture (Fig. 5) were found to impact the birds’ effective limb length (ELL) significantly (Fig. 6). Stance phase ELL (=hip height) tracks differences in absolute hindlimb length (Fig. 6a; Supplementary Notes 1), with Mallards significantly lower than the domesticated breeds. However, once normalised by functional limb length, the posture index (PI) effectively reverses, with Mallards having a significantly greater PI than the domestic breeds across much of stance (albeit with relatively small absolute differences), while Indian runners and Aylesburys show only marginal (and largely non-significant) differences from one another (Fig. 6b).

In addition, across all ducks midstance trunk pitch was not found to correlate with contemporary PI values but was moderately negatively correlated with midstance hip angle (Supplementary Notes 8). This trend was most pronounced in Indian runners, which showed a slight decrease in PI with increasing trunk pitch (adj. *R*^2^ = 0.24), as well as a notable decline in hip angle (adj. *R*^2^ = 0.5).

## Discussion

Models describing the mechanistic links between body proportions, limb posture and locomotor cost in tetrapods are numerous (e.g. refs. ^2,46,48–52^), and often well-supported by empirical data, in which species vary considerably in morphology and ecology (e.g. humans versus non-human apes^53,54^), or where they attempt to describe broad changes across very large ranges in body size (e.g^4,18–20,52^). However, few studies have directly addressed interactions between body morphology, gait and energetics at a finer-scale, either between closely related species that are more conservatively proportioned^34,35^ or intra-specifically^33,55,56^. In this study, we have combined 3D anatomical and experimental analyses to quantify the body shape, locomotor kinematics, and energetic cost of locomotion in three morphologically divergent breeds of duck. In agreement with theoretical predictions, we find evidence for a link between body shape and posture, reflecting the necessity to adopt postures that can accommodate different body segment proportions and mass distributions^5,11,12,21,25,51^. However, our results show ducks incur similar energetic costs during locomotion, despite their considerable differences in morphology and posture. This most likely originates from the interplay of multiple factors, namely modular interactions between body segments and a dynamic relationship between the limb joints and trunk pitching angle, which outweigh the size-related processes that (largely) govern locomotor costs at interspecific scales, via their relative influence on effective limb length.

The impact of selective breeding has led to considerable differences in skeletal and soft-tissue segment properties (Supplementary Notes 1, 2), resulting in near-complete segregation between duck breeds in multivariate analysis of their proportions (Fig. 1), as well as divergence in their whole-body CoMs (Fig. 2). As the ancestral morphotype, Mallards are found to have a CoM consistent with forelimb-dominant birds (Fig. 2; ref. ^12^), indicating relative investment in the forelimb soft tissues over the hindlimb (Supplementary Notes 2), reflective of greater wing-to-leg performance and the capacity for long-distance flight^56,57^. This sharply contrasts the domestic breeds, which have been rendered flightless through selective breeding.

In Aylesbury ducks, selection for increased meat yield has led to large increases in body mass via the deposition of extra-skeletal tissue, which is evidenced by similar skeletal convex hull volumes to Indian runners (indicative of equivalent 3D skeletal sizes; Supplementary Fig. 2), but notably larger skin segment volumes (Supplementary Notes 1) and the highest final body masses. Interestingly, the nature and magnitude of these additional soft tissues differ from that seen in other birds selectively bred for meat production. In broiler chickens^58^, and turkeys^59^, a disproportionate increase in breast muscle mass displaces the CoM cranially relative to their respective wild types. In Aylesbury ducks, however, CoM is comparatively caudal to Mallards and located within the hindlimb-dominant region of avian CoM morphospace (Fig. 2^12^; which can be attributed to approximately even increases in soft tissues along the cranio-caudal axis (Fig. 1B; Supplementary Notes 1).

Indian runner ducks present an unusual morphology as they possess the elongate, muscular hindlimbs common to terrestrial birds (Supplementary Notes 2^57^), yet plot within the forelimb-dominant region of CoM, with a CoM cranial to that of volant Mallards (Fig. 2). We suggest this may be explained by elongation of the trunk (greatest gleno-acetabular distance) and neck in this breed. In particular, neck and hindlimb lengths are thought to be highly integrated in birds (Fig. 3)^60^, so this would provide a direct mechanism through which the caudal impact of Indian runners’ large legs on CoM may be offset. These results, therefore, emphasise the possibility for modular processes to produce potentially non-intuitive changes in whole-body CoM, such that an obligate terrestrial breed is recovered with the most anatomically cranial CoM and plots amongst more volant taxa (Fig. 2).

Whole-body CoM has a considerable impact on the posture of a walking biped because: (1) the foot must be placed beneath the CoM around midstance to ensure static stability, and (2) the knee should be positioned cranially to the CoM so that a knee extensor moment is attained during and after midstance^5,11,12,21,25,26,51,61,62^. Therefore, in line with their disparate proportions and CoM positions (Figs. 1–3), we find that duck breeds also differ significantly in many aspects of their limb and trunk kinematics (Figs. 4, 5; Supplementary Notes 6), suggesting postural adjustment in response to the mechanical challenge of novel body shapes. Though all ducks are found to operate a fairly crouched limb, which is a common feature of small birds^5,21–23,25,32,35^, domestic breeds significantly depart from the kinematic strategy of Mallards (Fig. 5).

The Aylesbury duck walks with a relatively extended hip and knee (Fig. 5a, b), seemingly in alignment with its caudal CoM (Fig. 2). In addition, given that Aylesburys are the largest breed studied here, the combined demands of their additional absolute and relative soft-tissue mass (Supplementary Notes 1), favours extended postures that align the joints more closely with the vGRF, thereby reducing external joint moments whilst also allowing extensor muscles to operate with higher moment arms^4,18,40,63^. The relatively high duty factor of this breed (Fig. 4h) would additionally serve to lower peak forces by distributing force across a relatively longer stance phase, as has been suggested in other commercial poultry^59^. However, while Aylesburys walk with extended proximal joints, we also find the ankle to be the most flexed of the three breeds through stance (Fig. 5c). Though the cause of this is unclear, it presents a dichotomy with Mallards, which are proximally more flexed but utilise a significantly more extended ankle (Fig. 5a–c).

The cranial CoM of the Indian runner presents a clear postural challenge that appears to be accounted for by its dissimilar limb and (most noticeably) trunk kinematics (Figs. 2 and 5). While the displacements required to support a cranial CoM may be achieved through ‘crouching’ and increasing overall limb flexion (as we have suggested between Mallards and Aylesburys), our results show that Indian runners actually utilise some of the most extended hip and ankle postures amongst ducks (Fig. 5a), which is similar to more cursorial birds^22,23,52^, that tend to have much more caudal CoMs (Fig. 2^12^). This discrepancy between posture and body shape can be explained by the highly orthograde (upright) trunk posture used by Indian runners (Fig. 5d), which would serve to rotate the axial segments’ CoM caudally towards the hip, thereby facilitating more extended limb postures. This is further evidenced by the negative correlation between midstance trunk pitch and hip angle in this breed (Supplementary Notes 8), showing that as more pitched trunks are adopted, the hip becomes relatively extended. In our coordinate system, the net effect of this is that the femur is roughly horizontal irrespective of pitch level, and the distal limb remains relatively strut-like.

It is predicted that animals will use kinematics (joint and spatiotemporal) that are relatively optimised for their anatomy^2,5,11,12,23,34,35,46^, and that certain morphological (e.g. long limbs) and functional (e.g. upright limb posture) hallmarks bestow energetic benefits. For example, relatively long limbs (as seen in Indian runners) may allow for longer strides, lower muscle activations, and recruitment of slower, economical muscle fibres^6,7,48,49^; while relatively extended postures (as seen proximally in Aylesburys), should align the joints closer to the vGRF thereby lowering external forces and decreasing muscle activation costs^4,18^. However, despite their postural disparity, there is no appreciable difference in locomotor-specific costs between the duck breeds (net COT; Fig. 4d). ELL is considered one of the most reliable predictors of CoT across animals ranging widely in body size. However, it is necessarily size-dependent^48,49^. While differences in body size between these duck breeds are pronounced, they are probably less impactful than the multiple orders of magnitude that are usually represented in interspecific datasets (e.g.^4,5,19,49,52^), suggesting that size-specific savings may be relatively diminished. Therefore, normalising ELL to PI (Fig. 6) elucidates the impact of posture^33,52,64,65^, showing that breeds possess similar PIs regardless of their differences in limb proportions, body size, and kinematics (4% difference at midstance, Fig. 6b). This suggests that the larger domestic breeds, in particular the Indian runner, are not ‘long-limbed’ in a dynamic sense, and may incur penalties from their novel kinematic strategies that result in a PI equivalent to—or lower than—wild Mallards (Fig. 6b). For one, the significantly more crouched postures of the larger domesticated breeds could entail proportionally greater volumes of active muscle compared to Mallards, especially given that the force-generating ability of skeletal muscle becomes relatively diminished with increasing size^4,18,19^.

We also identify a potential role for trunk pitching in modulating PI in Indian runners, since this breed shows only a minor decrease in PI with increasing pitch, despite notable decreases in hip flexion (Fig. S7:1). Pitching may therefore allow Indian runners to operate more extended hip joints and maintain a comparable PI to the other breeds, thereby avoiding substantial limb flexion which would otherwise be necessary given their cranial CoM. That said, the minor decline in PI with increased pitch may indicate the existence of a threshold for pitching based on CoM and limb length. We find that the least pitched Indian runners tend to have higher PIs (Fig. S7:1A) and suggest that this may be caused by a reduction in the vertical contribution of the femur to ELL as the torso is rotated posteriorly, which remains to be examined.

Though convergence upon similar PI values may explain some of the energetic similarities between these duck breeds, there are other aspects that merit further consideration. For example, while our study has conducted a detailed analysis of gross skeletal and soft-tissue anatomy (Figs. 1–3), we have not investigated intrinsic tissue properties, and have therefore worked on an implicit assumption that these are relatively conserved across the breeds. In particular, muscle fibre lengths are typically tuned to specific kinematic ranges-of-motion so that they operate around the optimum of the force-length relationship^66,67^. Since it is difficult to argue that either domestic breed is under targeted selection for locomotor performance (even if selection has taken place upon their locomotor anatomy), it may be presumed that Mallard-like intrinsic muscle properties are retained in the two domestic breeds, that are potentially less suited for their different kinematic strategies. Likewise, we have not investigated the ability of each breed to exploit energy recovery mechanisms (elastic or mechanical), which may differ in accordance with their morphology and kinematics^3,24,29,30,32,34,46,52^. However, previous work on ducks by Usherwood et al.^30^, using the same breeds studied here, found evidence for greater mechanical energy savings in Aylesburys and Mallards over Indian runners, which occurred irrespective of whether lateral energy exchange was included (this is thought to contribute substantially to total energy recovery in waddling birds^24^). Consideration of elastic capabilities would, in a similar vein to muscle properties, require further analysis of the tendinous anatomy.

In summary, we find that selective breeding has led to pronounced differences in body shape between duck breeds, which presents varied mechanical challenges to stable bipedal locomotion and, subsequently, significantly different limb and trunk kinematics. Surprisingly however, this morphological and kinematic disparity does not appear to translate to appreciable differences in the energetics of locomotion, which we argue may occur through a many-to-one phenomenon, whereby dissimilar kinematics result in functional similarities and subsequently similar energetic costs. These results, therefore, underline the difficulty of predicting locomotor costs directly from gross skeletal morphology^48,49^, as the dynamic impact of repositioning CoM is multifaceted. Perhaps this is best exemplified by the Indian runner ducks, whose long hindlimbs are paired with an extremely cranial CoM via a general pattern of integration between the hindlimb and cervical segments in birds. To support this derived body shape, Indian runners utilise a combination of extreme trunk pitching and variable joint kinematics that entail a proportional reduction in ELL, which appears to negate the potential energetic benefits of their anatomically long legs. Therefore, when considering the marked changes in body shape hypothesised to underpin locomotor evolution in birds and non-avian theropods^11,12,36–38^, these results add further nuance to recent work by Macaulay et al.^12^, suggesting that the complex array of modular interactions between body shape and mass distribution across bird evolution, might be matched by equal (and probably non-linear) diversity of postures, with wide ramifications for the evolution of locomotor costs. Therefore, testing hypotheses about how the evolution of morphology may have incurred or been driven by changes to kinematics and energetics along the bird lineage, should consider locomotion as an integrated whole, in terms of whole-body morphology and mechanics, where seemingly unrelated features (e.g. neck elongation) may have a profound impact upon the overall system.

## Methods

### Study species

Adult (>8 months) male Aylesbury (*n* = 6), Mallard (*n* = 8) and Indian runner (*n* = 8) ducks were purchased from local breeders. These breeds were chosen because they show clear qualitative differences in body shape and posture, and ducks are not subject to the severe gait abnormalities found in other types of poultry (particularly chickens). Though ducks ancestrally present a generalised locomotor system (a trade-off between walking, flying and swimming), selective breeding has rendered the two domestic breeds flightless (neither were observed to take flight during our care). All breeds are therefore capable and frequent walkers, subject to constraints applicable to any biped, and appropriate to answer the question(s) of this study. In terms of selective pressures acting upon these breeds; Aylesburys are primarily reared for their meat; Indian runners were historically bred for their laying abilities, but since the 19th century, are also a popular show and ornamental birds; while Mallards represent the ancestral condition.

All ducks were kept at 17–22 °C, under a 12 h:12 h light–dark regime, in the Biological Services Facility within the University of Manchester. Birds were not fasted prior to experiments and access to food and water was provided ad libitum. Training trials were conducted on a motorised treadmill (Tunturi®, Finland) during the first week to establish aerobically sustainable speeds of locomotion. All experiments were approved by the University of Manchester Ethics Committee and conducted in accordance with the Animals (Scientific Procedures) Act 1986, under a UK Office project license (40/3567).

### Energetic and spatiotemporal kinematic analysis

Six Aylesburys (2.26 ± 0.06 kg), five Mallards (1.00 ± 0.03 kg) and six Indian runners (1.77 ± 0.04 kg) contributed data towards this part of the study. These birds were filmed laterally (HandyCam^®^ HDR-XR250, Sony Corporation, Japan, 100 frames per second) while exercising on a motorised treadmill during respirometry recordings. The nearest foot to the camera (right foot) was tracked using Tracker Software v. 4.97 (Open Source Physics) over 10 continuous strides to obtain stride length (${l}_{{{\rm{stride}}}}$), stride frequency $({f}_{{{\rm{stride}}}}),$ stance time (${t}_{{{\rm{stance}}}}$), swing time (${t}_{{{\rm{swing}}}}$) and duty factor (DF). Four speeds (*U*) were compared between breeds (0.28, 0.49, 0.56 and 0.69 m s^−1^).

Using open flow respirometry, we recorded the rate of oxygen consumption (${\dot{V}}_{{{{\rm{O}}}}_{2}}$) and carbon dioxide production (${\dot{V}}_{{{{\rm{CO}}}}_{2}}$) during treadmill locomotion and quiet standing, inside Perspex^®^ chambers adjusted for each breed size (mallards: 66 cm × 49 cm × 45.5 cm, Indian runners: 66 cm × 49 cm × 65.5 cm, Aylesbury: 66 cm × 49 cm × 56 cm). Air was pulled through the chamber at flow rates (FR) of 210 L min^−1^ (Indian runners), 181 L min^−1^ (Aylesbury ducks) and 150 L min^−1^ (mallards) using a Flow-Kit 500 (Sable Systems International, Las Vegas, USA). A sub-sample of excurrent air was taken from the main flow at 0.1 L min^−1^ for gas analysis. Firstly, water vapour pressure was quantified using an RH-300 water vapour analyser (Sable Systems International, Las Vegas, USA) prior to scrubbing it from the airstream with calcium chloride (2–6 mm granular, Merck, Darmstadt, Germany). CO_2_ was then quantified using a CA-10a analyser (Sable Systems International, Las Vegas, USA) and scrubbed using soda lime pellets with indicator (2–5 mm, Merck, Darmstadt, Germany). The dry and CO_2_-free air then passed through the first channel of a dual absolute and differential Oxilla-II O_2_ analyser. A parallel dried and CO_2_-free ambient air sample was simultaneously pumped at 0.1 L min^−1^ into the second channel of the O_2_ analyser to enable the calculation of the differential O_2_ concentration ($\triangle {{{\rm{O}}}}_{2}$). A UI2 data acquisition interface and ExpeData^®^ v 1.1.15 software (Sable Systems International, Las Vegas, USA) were used to record and interpret respirometry data. We performed an N_2_ dilution test^68^ to test the respirometry system accuracy (±5% across all treadmill speeds).

Since H_2_O was scrubbed from the airstream, the main flow rate used to determine metabolic rate was adjusted (FR_c_) using:

1 $${{{\rm{FR}}}}_{{{\rm{c}}}}=\frac{{{\rm{FR}}}\cdot ({{\rm{BP}}}-{{\rm{WVP}}})}{{{\rm{BP}}}}$$

where BP is the barometric pressure and WVP is the water vapour pressure. ${\dot{V}}_{{{{\rm{O}}}}_{2}}$ was then calculated as

2 $${\dot{V}}_{{{{\rm{O}}}}_{2}}=\frac{{{{\rm{FR}}}}_{{{\rm{c}}}}\left(\triangle {{{\rm{O}}}}_{2}\right)}{1-0.2095}$$

and $${\dot{{{\rm{V}}}}}_{{{{\rm{CO}}}}_{2}}$$ using

3 $${\dot{V}}_{{{{\rm{CO}}}}_{2}}=\frac{\left({{{\rm{FR}}}}_{{{\rm{c}}}}\left(\triangle {{{\rm{CO}}}}_{2}\right)\right)-\left(0.0004\left({\dot{V}}_{{{{\rm{O}}}}_{2}}\right)\right)}{1-0.0004}$$

Equations (1)–(3) were from Lighton^69^. All ducks were exercised over a range of *U* up to their maximum sustainable speed: 0.75 m s^−1^ in Mallards, 0.83 m s^−1^ in Aylesbury ducks, 1.11 m s^−1^ in Indian runners. A trial consisted of a bird walking at three randomly selected speeds per day, in each case, until stable recordings of O_2_ and CO_2_ were obtained. After each speed, ducks were observed while standing quietly for at least 5 min to enable measurement of standing metabolic rate, a proxy for resting metabolic rate.

Respirometry exchange ratios (RER) were calculated as ${\dot{V}}_{{{{\rm{CO}}}}_{2}}$:${\dot{V}}_{{{{\rm{O}}}}_{2}}$. RER ≤ 1.00 was indicative of aerobic-metabolism. These values were then used to estimate absolute metabolic power (*P*_met_) using the thermal equivalencies described by Brody^70^, which were later divided by the mass of each duck to obtain mass-specific *P*_met_. Mass-specific *P*_met_ comprises the energetic costs of locomotion, body posture^71^, critical physiological processes (e.g. breathing, circulation) and experimental stress^72^. Thus, subtracting the standing metabolic rate (W kg^−1^) of all birds while standing quietly from ${{\rm{mass}}}{{\rm{\hbox{-}}}}{{\rm{specific}}}{P}_{{{\rm{met}}}}$, allows us to estimate ${{\rm{net}}}\mbox{-}{P}_{{{\rm{met}}}}$. The cost of transport (CoT) and the net cost of transport (net-CoT) were then estimated by dividing ${{\rm{mass}}}{{\rm{\hbox{-}}}}{{\rm{specific}}}{P}_{{{\rm{met}}}}$ and ${{\rm{net}}}\mbox{-}{P}_{{{\rm{met}}}}$ for (*U*), respectively.

To compare the locomotor economy of ducks relative to other birds, we performed an ordinary least-squares regression of body mass and minimum CoT using published data for 11 avian taxa spanning three orders of magnitude. Following this, we performed additional ordinary and phylogenetic generalised least-squares regressions of the dataset when ducks were included, allowing us to generate new bird-specific allometric equations for minimum CoT (see Supplementary Notes 5) that be compared against previous work on birds and mammals^73^.

ANCOVA with Tukey post-hoc tests was used to analyse the interaction between energetics and kinematic parameters with *U* as a covariate, to identify differences in the slopes and intercepts in the three duck breeds (factors). Models were simplified by removing the interaction term (*U* × BREED) if non-significant, indicating a common slope. The resulting model was further simplified if differences in intercepts (factor BREED) were also absent. When required, the kinematic parameters were transformed to log_10_ to meet the assumption of normally distributed data.

Size-normalised comparisons were also performed. The three breeds of duck differ from each other mainly in body size, hindlimb length and body posture. Thus, we have used the square root of the Froude number ($\hat{U}=U/\sqrt{{h}_{{{\rm{hip}}}}\times g}$) as a mechanism to equalise the ratios of inertial and gravitational forces acting over the CoM of each bird^74,75^, where ${h}_{{{\rm{hip}}}}$ is hip height and *g* is gravity, allowing us to better understand the cumbersome effects that size has on the kinematics and energetics of terrestrial locomotion. Given that we did not measure ${h}_{{{\rm{hip}}}}$ directly from the birds in vivo, we used the functional limb length (i.e. the sum of the length of the femur, the tibiotarsus and the tarsometatarsus) as a proxy of ${h}_{{{\rm{hip}}}}$. All kinematic parameters were also transformed by relating them to ${h}_{{{\rm{hip}}}}$ and $g$ following^74^): relative stride length (${\hat{l}}_{{{\rm{stride}}}}={l}_{{{\rm{stride}}}}/{h}_{{{\rm{hip}}}}$), relative stride frequency (${\hat{f}}_{{{\rm{stride}}}}={f}_{{{\rm{stride}}}}/\sqrt{g/{h}_{{{\rm{hip}}}}}$), relative stance (${\hat{t}}_{{{\rm{stance}}}}={t}_{{{\rm{stance}}}}/\sqrt{{h}_{{{\rm{hip}}}}/g}$) and relative swing phase (${\hat{t}}_{{{\rm{swing}}}}={t}_{{{\rm{swing}}}}/\sqrt{{h}_{{{\rm{hip}}}}/g}$). DF was not transformed because of its dimensionless nature. Linear models were performed with $\hat{U}$ as covariate and BREED as a factor. All the analyses were conducted in R v.3.6.6^76^.

### Joint kinematic and dynamic postural analysis

3D body segment kinematics were recorded at 200 Hz, using 19 reflective markers and a 12-camera Qualisys Oqus 7 motion capture system (Qualisys Inc., Götenborg, Sweden), during continuous bouts of treadmill walking in two Aylesburys (2.3 ± 0.1 kg), three Mallards (1.07 ± 0.03 kg), and three Indian runners (1.58 ± 0.02 kg). Markers were placed on the midline between the shoulder joints, on the cranial and caudal areas of the pelvis, and either side of the midline centrally between those aforementioned pelvic markers. Two markers were placed proximally and distally on both thigh, shank and tarsometatarsus segments, and a single marker was placed distally on digit III of both feet. Each bird was recorded walking for 45 s at a similar Froude number (breed averages: Aylesbury = Fr 0.215, Mallard = Fr 0.194, Indian runner = Fr 0.199, following Alexander^73^), towards the upper end of their studied speed range. Unlike humans, birds do not experience discrete shifts in gait with speed^5,27,29,32^, therefore we expect that postural differences between the breeds will persist continually across their grounded speed range. 3D kinematic data was collected in separate trials from the respirometry data because we found the respirometry chamber and equipment impeded camera placement and overall data quality.

Marker data was tracked in Qualisys (v.2.15). and exported for inverse kinematic analysis in OpenSIM (v.4.3). A total of 30 strides were analysed for each breed, which was sourced equally across individuals (ten per individual Mallard and Indian runner, fifteen per Aylesbury) and comprised a single episode of activity. The data is derived exclusively from the left limb, which was found to have superior marker quality to the right. A rigid body OpenSIM model was constructed in NMSBuilder for one individual of each breed, using their 3D skin volumes, bones, and kinematic markers segmented from CT data (as described below). The model consisted of a combined axial and forelimb body (with fixed joints), attached to a hindlimb linked by active joints. The hip joints were modelled as a ball-and-socket joint (allowing flexion-extension, long-axis rotation and abduction–adduction) and the knee, ankle and tarsometatarsophalangeal joints were modelled as hinges, allowing only flexion-extension. These subject-specific models were then scaled according to linear skeletal proportions to produce model variants for use on other individuals of the same breed.

We performed statistical analysis on flexion-extension at the hip, knee and ankle, as well as trunk pitch. Angles were exported for every 0.05 s interval, and delimited into strides (stance and swing phases) based on toe marker position. The following parameters were analysed by Kruskal–Wallis tests; maximum flexion/pitch, minimum flexion/pitch and joint/trunk angles at foot contact, take-off and midstance. While analyses of these metrics allow kinematic comparisons at specific time points or gait events, it has the limitation of treating these time points in isolation (i.e. as statistically independent from the rest of the gait cycle). Therefore, we also investigated broader differences across the entire gait cycle with 1D statistical parametric mapping (SPM)^77^; using code adapted from Grant et al.^78^, implemented in Matlab (v. R2022a).

The impact of posture in animals with different limb lengths and proportions is difficult to assess from joint angles alone. Therefore, we were also interested in the interplay between flexion/extension and limb length, as a relatively high degree of joint flexion may lower the effective length of the limb (ELL), which in turn may impact upon energetic benefits associated with longer limbs^33,48,49^. ELL was calculated as absolute hip height across the stride (taken as the distance between the hip joint and the ground), by fixing a weightless marker to the hip joint centre on our OpenSIM models and exporting its position during the inverse kinematics. To assess the influence of posture on ELL, we divided it by functional limb length (femur + shank + tarsometatarsus lengths), to derive a posture index (PI), and analysed both measurements via an SPM procedure identical to the joint kinematics. Midstance PIs and hip angles were then regressed against midstance trunk pitch to evaluate a potential role for pitching in modulating overall limb posture (see Supplementary Notes 8).

### Morphological analysis

Following the experimental trials, the ducks were euthanised and CT scanned at the University of Liverpool Small Animal Teaching Hospital (Toshiba Aquilion PRIME helical scanner, slice thickness: 0.5 mm, 120 kVp, 100 mA). The CT data was segmented in Mimics (v. 23.0), following the approach of Macaulay et al.^12,79^. This involves generating 3D skeletal and skin volume models of the whole bird, which are then separated into discrete body segments. In addition, the skeletal segments were convex hulled in accordance with previous studies^12,80,81^, as minimum convex hulls serve multiple distinct analytical purposes. First, they provide a more representative value for absolute and relative size of certain body segments (e.g. torso, head) than single linear measurements^17^. Second, they facilitate a comparison of the relative volumetric size of skeletal body segments to the full skin volume of body segments (i.e. a measure of skeletal to extra-volume ratio).

Differences between the breeds in terms of their skeletal and soft-tissue anatomy were identified through statistical comparison of the skeletal segment lengths, minimum convex hull volumes and skin segment volumes. In total, 14 linear measurements were compared, including five from the hindlimb; femur length, tibiotarsus length, tarsometatarsus length, digit III length, and total hindlimb length; five from the forelimb, humerus length, forearm length, carpometacarpus length, phalangeal length, and total forelimb length; as well as hip width, shoulder width, gleno-acetabular distance, sternum length, and neck length. Volumetric measurements (convex hulls and skin segments) totalled 11 for each category and comprised; head, neck, torso, humerus, forearm, manus, thigh, shank, tarsometatarsus, and pes. Both size-normalised and raw segment proportions were analysed, though we focus on the size-normalised comparisons, which allow us to identify differences in relative segment proportions (i.e. body shape) between breeds. Linear parameters were normalised by body mass^0.33^, while volumetric parameters were normalised by their corresponding total (whole-body) volume.

A one-way ANOVA was performed on all individual parameters, irrespective of category, to identify statistical differences between the three breeds. Analysis was undertaken in R Studio v.4.0.5^76^, using the anova_test function of Rstatix v.0.7.0^82^. Tukey HSD was used for post-hoc multiple pairwise comparisons using the tukey_test function of Rstatix v.0.7.0. Each parameter was tested individually *post hoc* to ensure that it met the assumptions of ANOVA. Where this was not the case, a second test was run with the problematic datapoints removed, and this was compared against the original result to identify qualitative changes.

Visualisation of breed-specific differences in morphology was performed through principal component analysis (PCA) of the normalised linear body segment lengths, minimum convex hulls, and skin volumes. PCA was undertaken using the multivariate statistics package FactoMineR (v. 2.4., ref. ^83^), in R Studio (v. 4.0.5.). Four separate analyses were performed for the linear segment lengths; all parameters combined, hindlimb parameters only, forelimb parameters only, and non-appendicular parameters only, which permitted comparison between the different anatomical regions. For the minimum convex hull and skin volume parameters, a single combined analysis was conducted for each category.

To determine if differences in body proportions affected CoM position between the breeds, we realigned the final skin segment volumes from one individual of each breed into a standardised reference pose (following Macaulay et al.^12^), and computed segment CoM in Meshlab v.2022.02. The segment CoM values were then combined to generate a whole-body CoM, which was then incorporated into the dataset of CoM positions for 33 extant birds from Macaulay et al.^12^ for wider context and comparison. To ensure a fair comparison to the dataset of the original authors, we replicated their choice of body segment densities, using 1000 kg m^−3^ for all segments except the neck (800 kg m^−3^) and torso (850 kg m^−3^).

Previous studies have suggested that within birds, the neck and hindlimb show modular coupling in terms of their total length^60^. It may be expected, therefore, that changes to either region within the ducks would result in corresponding changes to the other, which would potentially negate some of the impact of regional elongation upon whole-body CoM. To investigate integration between these two segments, we plotted raw neck length against raw total and functional hindlimb lengths and used ordinary least-squares regression to estimate correlation strength and significance.

### Reporting summary

Further information on research design is available in the Nature Portfolio Reporting Summary linked to this article.

### Supplementary information

Supplementary Information

Description of Additional Supplementary Materials

Supplementary Data 1

Reporting Summary

## Data Availability

All numerical data used in this study is available in Supplementary Data 1. 3D segmental data and OpenSim model files are available at 10.17638/datacat.liverpool.ac.uk/2734^84^.

## Code availability

The analytical code needed to repeat the analyses is available at 10.17638/datacat.liverpool.ac.uk/2734^84^.
