# Supplementary material for: Testing the form-function paradigm: body shape correlates with kinematics but not energetics in selectively-bred birds
Source: Commun Biol. 2024 Jul 24;7:900. doi: 10.1038/s42003-024-06592-w (PMC11269648; doi:10.1038/s42003-024-06592-w)
Supplement: Supplementary file 1 — Supplementary Information [file 42003_2024_6592_MOESM1_ESM.pdf]

SUPPLEMENTARY MATERIALS FOR: **TESTING THE FORM-FUNCTION PARADIGM: BODY SHAPE CORRELATES WITH KINEMATICS BUT NOT ENERGETICS IN SELECTIVELY-BRED BIRDS**

Samuel R. R. Cross*, Andres C. Marmol-Guijarro, Karl T. Bates, John C. Marrin, Peter G. Tickle, Kayleigh A. Rose, Jonathan R. Codd

*Corresponding Author. Email: Samuel.Cross@liverpool.ac.uk

This document includes:

Supplementary Notes 1: Extended results for the statistical analysis of the non-normalised body segments, including supplementary figures 1-6 and supplementary tables 1-5.

Supplementary Notes 2: Extended results for the statistical analysis of the normalised body segments, including supplementary figures 7-12 and supplementary tables 6-8.

Supplementary Notes 3: Extended results for the principal component analysis of normalised body segments, including supplementary figures 13-16, and supplementary tables 9-20.

Supplementary Notes 4: Modelling results for non-normalised energetic and spatiotemporal kinematic data, including supplementary figure 17 and supplementary table 21.

Supplementary texts S1 – S6

Supplementary Notes 5: Linear regression analysis of avian cost of transport, including supplementary figure 18 and supplementary table 22.

Supplementary Notes 6: Statistical analysis of discrete kinematic parameters, including supplementary figures 19-26 and supplementary tables 23-26.

Supplementary Notes 7: Positional relationship between the foot and centre of mass, including supplementary figure 27.

Supplementary Notes 8: Influence of trunk pitching on effective limb length, including supplementary figure 28 and supplementary tables 27-28.

Additional Supplementary Figures: contains supplementary figures 29-30.

Other Supplementary Materials for this manuscript include the following:

Supplementary Dataset S1 – S13

Supplementary Notes 1: Statistical comparisons of absolute segment sizes

Extended Results

As detailed in the main text, the breeds differed in total body size (mass + volume), with mallards being consistently the smallest, followed by Indian runners the Aylesbury ducks (though the latter reversed for the minimum convex hulls). However, size differences may not be uniform across individual body segments, so this section provides statistical comparison of absolute segment sizes.

ANOVA of the non-normalised linear and volumetric parameters found contrasting proportions between the breeds, which can predominately be attributed to absolute size differences between them, though with specific considerations depending on parameter type (this trend is more noticeable for the soft-tissue than skeletal parameters), and breed (differences are most pronounced between the domestic breeds and the mallard, and less so between the domestic breeds themselves) (Supplementary Figures 1-3; Supplementary Tables 1-3).

Prior and post-hoc tests found the data largely fit the test assumptions of ANOVA, however there were issues with several parameters, namely; thigh length, thigh hull volume, and neck skin volume were found to contain statistically extreme outliers using the *identify_outliers* function of RStatix v.0.7.0. (*1*); thigh length, manus hull volume, thigh hull volume, TMT hull volume, thigh skin volume, and pes skin volume were found to possess residuals which deviated substantially from normality (Supplementary Figures 4-6); and neck skin volume, torso skin volume, manus skin volume, thigh skin volume, and TMT volume were determined to be heteroscedastic using the *levene_test* function of RStatix v.0.7.0. (*1*). For each of these parameters we ran either, (1) a second ANOVA with the anomalous datapoints removed from analysis (Supplementary Table 4), or (2) a Welch's ANOVA in the instances were only the homoscedasticity assumption was violated (Supplementary Table 5). Comparison of these results with the original tests found qualitative differences in only two parameters (discussed below).

For the linear skeletal measurements, Indian runners were found to possess the longest limbs and neck, widest hips and greatest GA distance; Aylesbury's were found the have the widest shoulders, while mallards were the smallest breed in all absolute proportions (Supplementary Figure 1; Supplementary Table 1). All parameters were found to significantly differ between at least one breed, with Indian runners and mallards shown to be the most divergent (all parameters significantly differed), followed by Aylesbury's and mallards (all parameters besides hip width), then Indian runners and Aylesbury's (the distal limbs (CMC, Manus and Pes lengths) and sternum length were found to not significantly differ).

For the volumetric parameters (minimum convex hulls and skin segments), there were again pronounced differences between the mallards and the domestic breeds (Supplementary Figures 2-3; Supplementary Tables 2-3). Convex hull volumes were significantly smaller in mallards, while the other breeds differed only in the manus volume, which was greater in the Aylesbury (Supplementary Figure 2; Supplementary Table 2). In contrast, skin segment volumes were found to significantly differ across all three breeds, reflecting their absolute size differences (Supplementary Figure 3; Supplementary Table 3). The only exception to this trend were the thigh and torso volumes, which did not significantly differ between Aylesbury's and Indian runners, once they had been corrected for violation of test assumptions (Supplementary Tables 4-5).

These results add nuance to the assessment of overall body size. Mallards remain statistically smaller than the domestic breeds in all segments analysed, however, Aylesbury's and Indian runners show an asymmetry in soft tissue deposition (Supplementary Figures 1-3; Supplementary Tables 1-3). Indian runners are found to possess a more elongate skeleton than Aylesbury's (Supplementary Figure 1; Supplementary Table 1), and have similar skeletal volumes (Supplementary Figure 2; Supplementary Table 2), yet final skin segment volumes are significantly greater in Aylesbury's. This indicates a massive absolute increase in soft tissue in Aylesbury's compared to Indian runners.

Figures & Tables accompanying Supplementary Notes 1

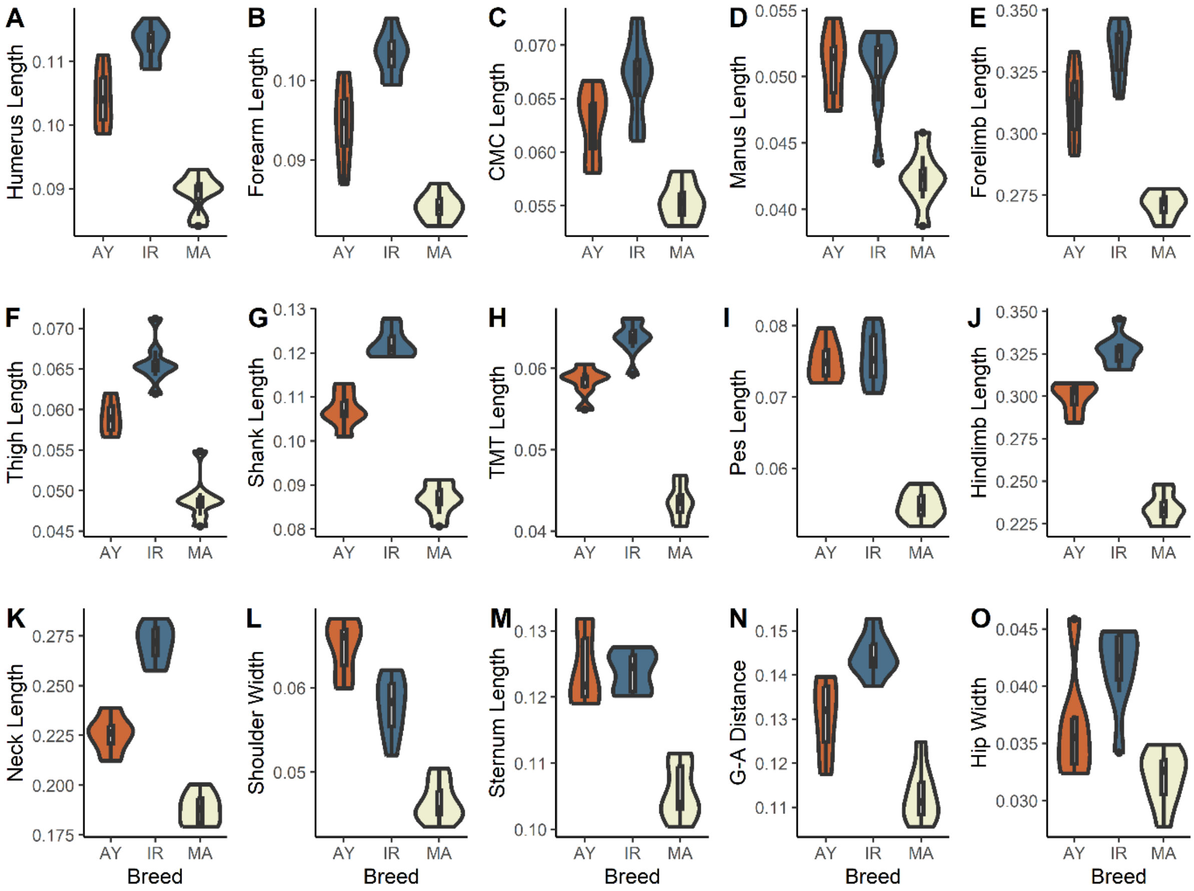

Supplementary Figure 1. Violin plots showing the primary statistics of each of the non-standardised linear measurements used in this analysis; **A-E**, forelimb parameters; **F-J**, hind limb parameters; **K-O**, non-appendicular parameters.

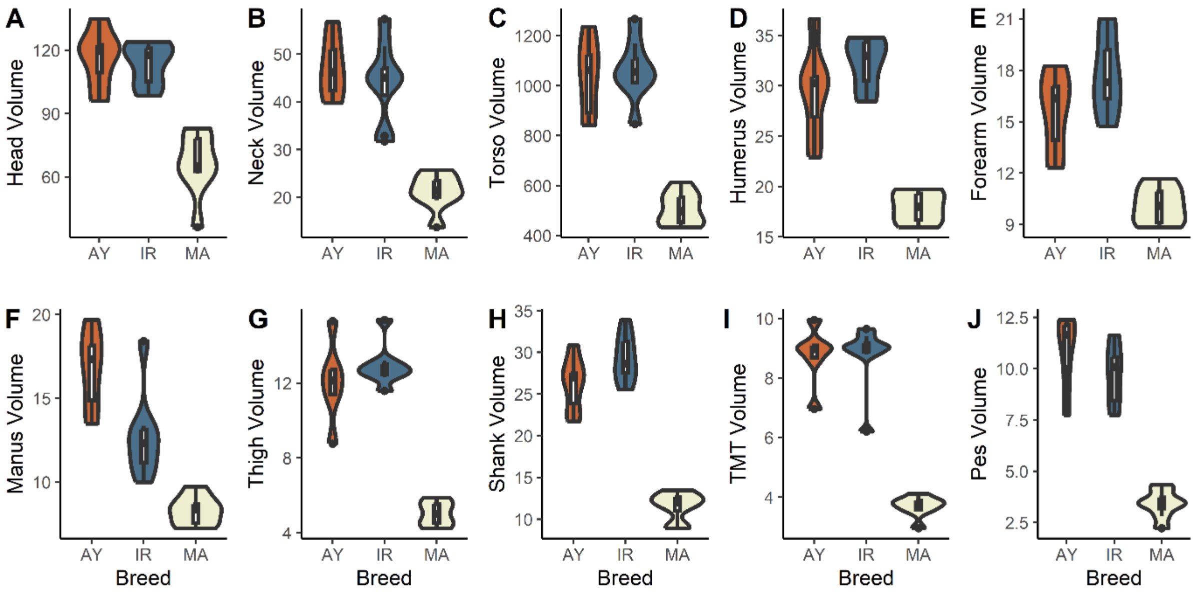

Supplementary Figure 2. Violin plots showing the primary statistics for each of the non-standardised minimum convex hull volumes used in this analysis; **A-C**, axial parameters; **D-F**, forelimb parameters; **G-J**, hind limb parameters. Volumes given in cm^3.

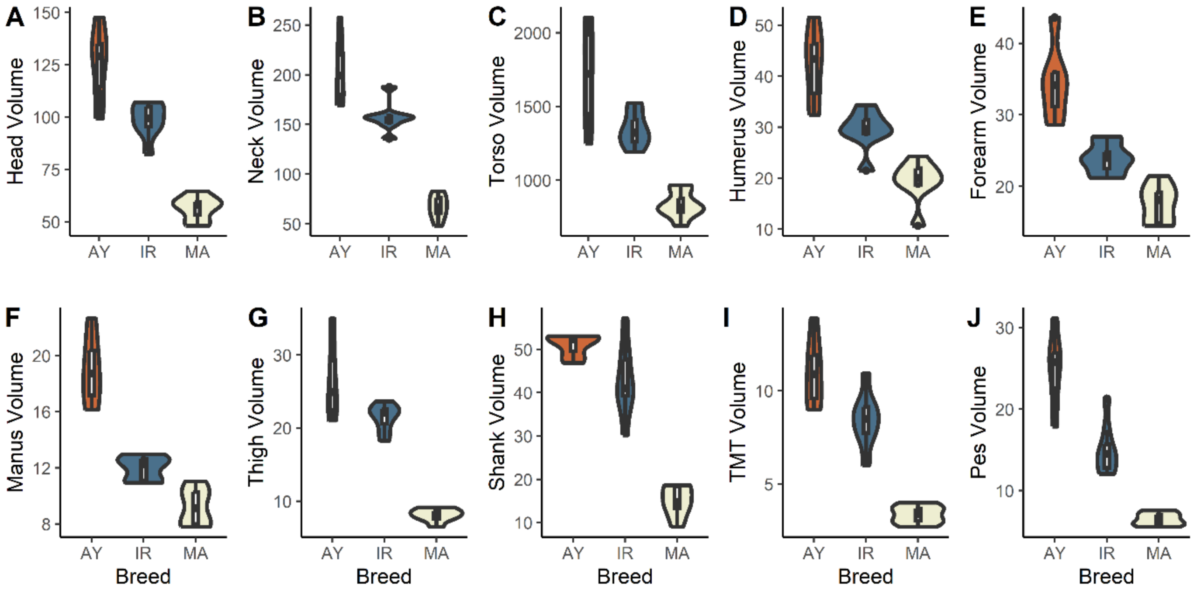

Supplementary Figure 3. Violin plots showing the primary statistics for each of the non-standardised skin segment volumes used in this analysis; **A-C**, axial parameters; **D-F**, forelimb parameters; **G-J**, hind limb parameters. Volumes given in cm^3.

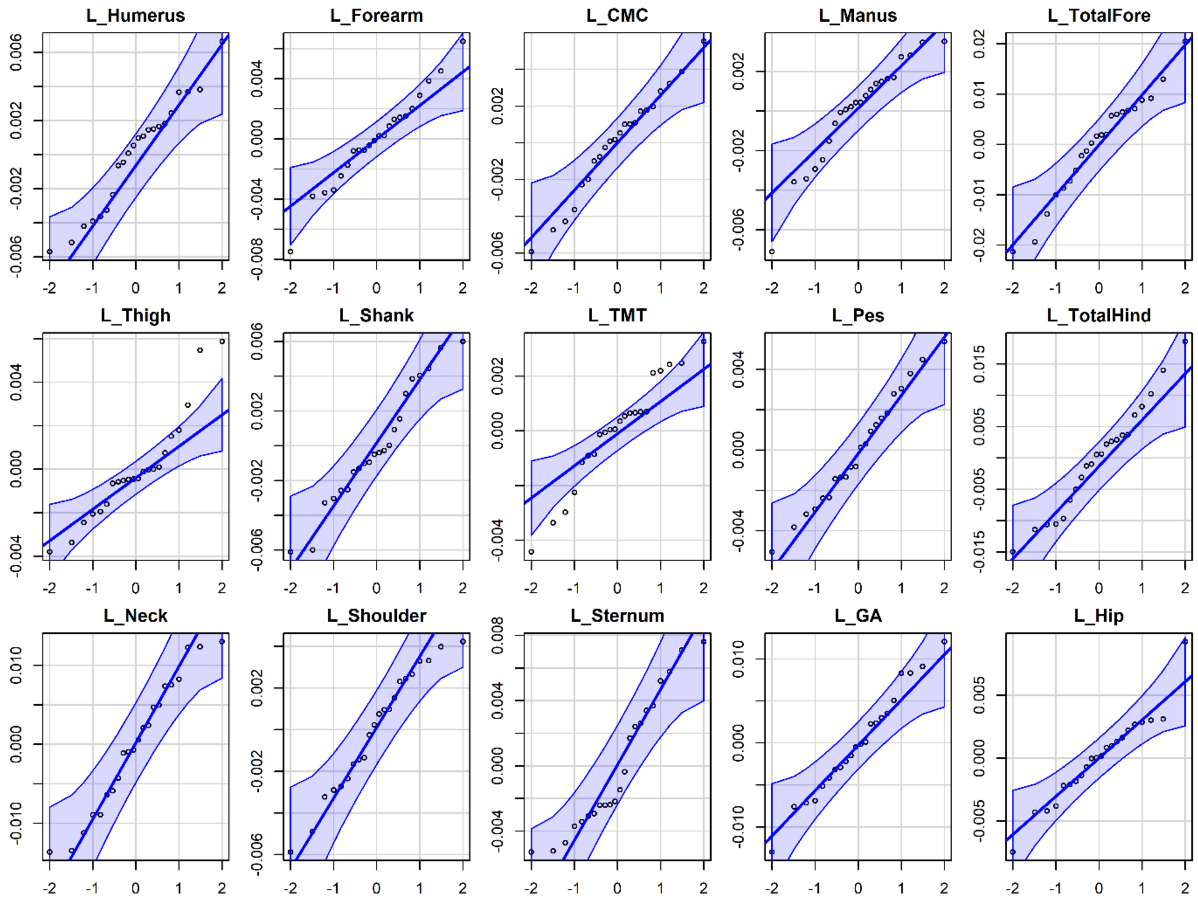

Supplementary Figure 4. QQ-plots showing the distribution of test residuals versus a normal distribution, for each of the non-standardised linear measurements. Normality was assessed by eye from these plots, on the condition that the datapoints should approximate the normal distribution (but can show some deviation, particularly towards the ends). We determined that thigh length contained non-normal data.

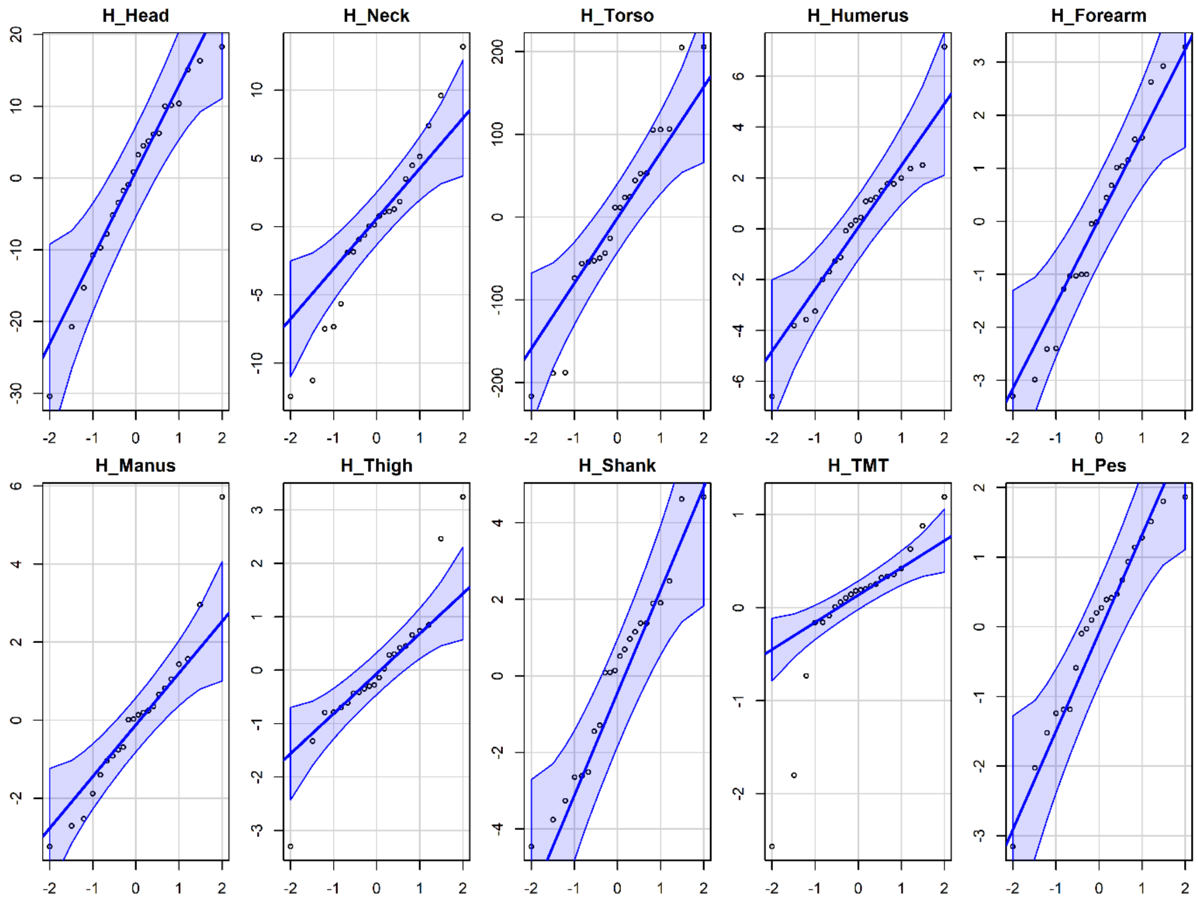

Supplementary Figure 5. QQ-plots showing the distribution of test residuals versus a normal distribution, for each of the non-standardised convex hull volumes. Normality was assessed by eye from these plots, on the condition that the datapoints should approximate the normal distribution (but can show some deviation, particularly towards the ends). We determined that the manus, thigh, and TMT hull volumes contained non-normal data.

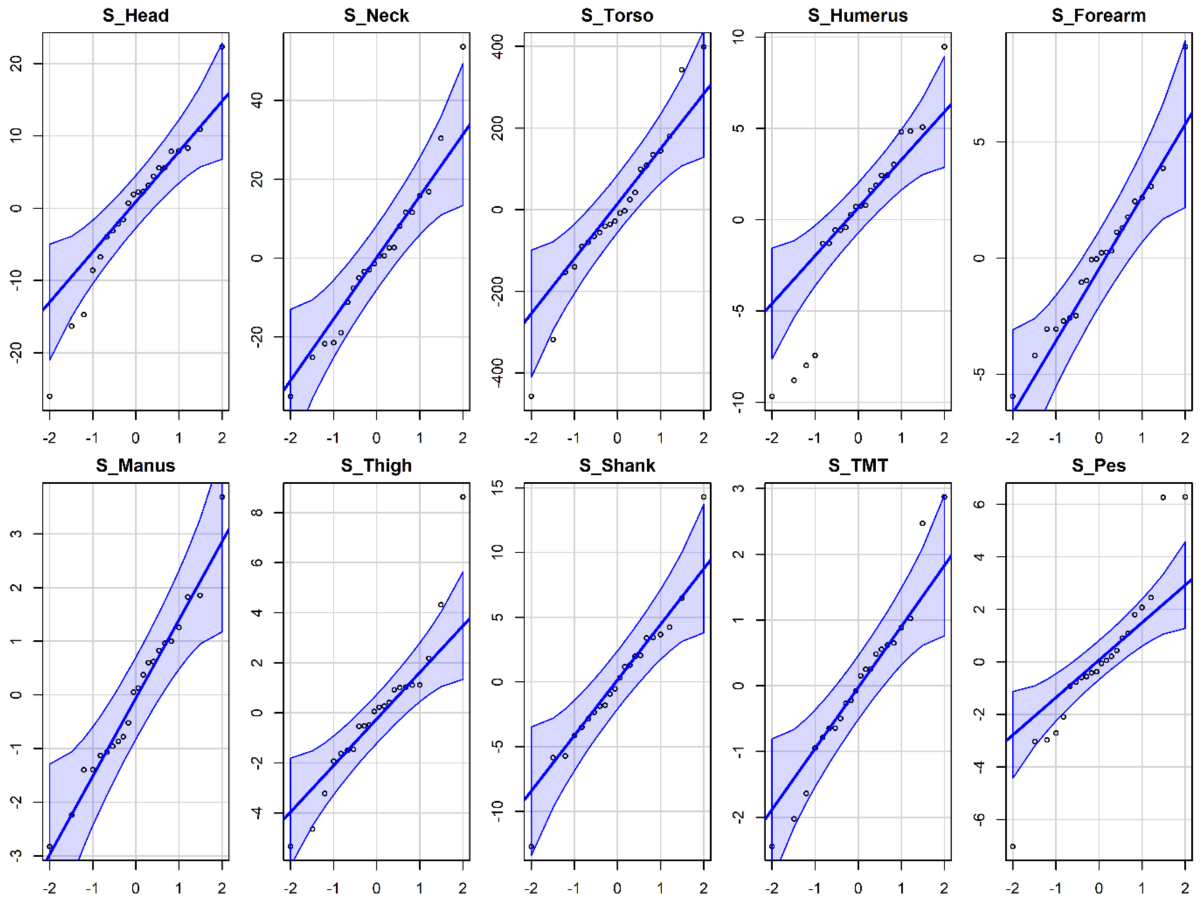

Supplementary Figure 6. QQ-plots showing the distribution of test residuals versus a normal distribution, for each of the non-standardised skin segment volumes. Normality was assessed by eye from these plots, on the condition that the datapoints should approximate the normal distribution (but can show some deviation, particularly towards the ends). We determined that the thigh and pes skin volumes contained non-normal data.

Parameter	ANOVA			Tukey HSD (p)		
	F	p	η^2	AY - IR	AY - MA	IR - MA
Humerus Length	95.84	<0.001*	0.91	0.001*	<0.001*	<0.001*
Forearm Length	68.275	<0.001*	0.88	<0.001*	<0.001*	<0.001*
CMC Length	29.51	<0.001*	0.76	0.050	0.001*	<0.001*
Manus Length	23.543	<0.001*	0.71	0.977	<0.001*	<0.001*
Forelimb Length	67.763	<0.001*	0.88	0.005*	<0.001*	<0.001*
Thigh Length	87.648	<0.001*	0.90	<0.001*	<0.001*	<0.001*
Shank Length	197.331	<0.001*	0.95	<0.001*	<0.001*	<0.001*
TMT Length	197.883	<0.001*	0.95	<0.001*	<0.001*	<0.001*
Pes Length	124.204	<0.001*	0.93	0.958	<0.001*	<0.001*
Hindlimb Length	220.385	<0.001*	0.96	<0.001*	<0.001*	<0.001*
Neck Length	180.686	<0.001*	0.95	<0.001*	<0.001*	<0.001*
Shoulder Width	65.974	<0.001*	0.87	0.001*	<0.001*	<0.001*
Sternum Length	45.956	<0.001*	0.83	0.990	<0.001*	<0.001*
GA Distance	46.716	<0.001*	0.83	0.003*	<0.001*	<0.001*
Hip Width	13.978	<0.001*	0.60	0.047*	0.077	<0.001*

Supplementary Table 1. ANOVA and Tukey HSD test results for all non-standardised linear segment lengths. For ANOVA, the F statistic (*F*), significance value (*p*), and estimated effect size (η^2) are presented, while N = 22 and D.F = 2. For the Tukey results the significance value (*p*) of each pairwise comparison is provided.

Parameter	ANOVA			Tukey HSD (p)		
	F	p	η^2	AY - IR	AY - MA	IR - MA
Head Volume	35.827	<0.001*	0.79	0.916	<0.001*	<0.001*
Neck Volume	34.787	<0.001*	0.79	0.697	<0.001*	<0.001*
Torso Volume	53.86	<0.001*	0.85	0.868	<0.001*	<0.001*
Humerus Volume	50.185	<0.001*	0.84	0.226	<0.001*	<0.001*
Forearm Volume	31.873	<0.001*	0.77	0.142	<0.001*	<0.001*
Manus Volume	28.966	<0.001*	0.75	0.005*	<0.001*	0.001*
Thigh Volume	80.395	<0.001*	0.89	0.489	<0.001*	<0.001*
Shank Volume	101.078	<0.001*	0.91	0.0993	<0.001*	<0.001*
TMT Volume	90.281	<0.001*	0.91	1	<0.001*	<0.001*
Pes Volume	65.504	<0.001*	0.87	0.305	<0.001*	<0.001*

Supplementary Table 2. ANOVA and Tukey HSD test results for all non-standardised minimum convex hull volumes. For ANOVA, the F statistic (F), significance value (p), and estimated effect size (η^2) are presented, while N = 22 and D.F = 2. For the Tukey results the significance value (p) of each pairwise comparison is provided.

Parameter	ANOVA			Tukey HSD (p)		
	F	p	η^2	AY - IR	AY - MA	IR - MA
Head Volume	71.95	<0.001*	0.88	<0.001*	<0.001*	<0.001*
Neck Volume	81.837	<0.001*	0.90	0.001*	<0.001*	<0.001*
Torso Volume	34.384	<0.001*	0.78	0.009*	<0.001*	<0.001*
Humerus Volume	34.249	<0.001*	0.78	0.001*	<0.001*	0.002*
Forearm Volume	42.961	<0.001*	0.82	<0.001*	<0.001*	0.004*
Manus Volume	67.756	<0.001*	0.88	<0.001*	<0.001*	0.006*
Thigh Volume	70.162	<0.001*	0.88	0.02*	<0.001*	<0.001*
Shank Volume	84.406	<0.001*	0.90	0.036*	<0.001*	<0.001*
TMT Volume	62.465	<0.001*	0.87	0.005*	<0.001*	<0.001*
Pes Volume	62.747	<0.001*	0.87	<0.001*	<0.001*	<0.001*

Supplementary Table 3. ANOVA and Tukey HSD test results for all non-standardised skin segment volumes. For ANOVA, the F statistic (F), significance value (p), and estimated effect size (η^2) are presented, while N = 22 and D.F = 2. For the Tukey results the significance value (p) of each pairwise comparison is provided.

Parameter	ANOVA			Tukey HSD (*p*)		
	F	*p*	η^2	AY - IR	AY - MA	IR - MA
Thigh Length[1,2]	174.137	<0.001*	0.953	<0.001*	<0.001*	<0.001*
Manus Hull Volume[2]	50.217	<0.001*	0.848	<0.001*	<0.001*	0.001*
Thigh Hull Volume[1,2]	338.755	<0.001*	0.977	0.443	<0.001*	<0.001*
TMT Hull Volume[2]	509.527	<0.001*	0.984	0.999	<0.001*	<0.001*
Neck Skin Volume[1,3]	138.413	<0.001*	0.945	0.001*	<0.001*	<0.001*
Thigh Skin Volume[2,3]	112.853	<0.001*	0.926	0.051	<0.001*	<0.001*
Pes Skin Volume[2]	67.386	<0.001*	0.888	<0.001*	<0.001*	<0.001*
Humerus Length[2]	82.371	<0.001*	0.902	<0.001*	<0.001*	0.001*
Shoulder Width[1,2]	2.994	0.075	0.25	0.663	0.069	0.25
GA Distance[1,2]	59.733	<0.001*	0.875	<0.001*	<0.001*	<0.001*
Head Hull Volume[1,2]	24.316	<0.001*	0.741	0.969	<0.001*	<0.001*
Neck Hull Volume[1]	0.071	0.932	0.008	0.961	0.999	0.934
Humerus Skin Volume[2]	6.939	0.006*	0.435	0.149	0.296	0.004*
Forearm Skin Volume[1]	23.357	<0.001*	0.722	0.003*	0.149	<0.001*
Pes Skin Volume[1,2]	96.564	<0.001*	0.915	<0.001*	<0.001*	<0.001*

Supplementary Table 4. ANOVA and Tukey HSD results for non-standardised (1st half, normal font), and standardised (2nd half, italics) parameters, whose raw data violated at least one assumption of ANOVA. The table presents the ANOVA results for each parameter following removal of the problematic datapoints, and therefore provides a comparison with the uncorrected results. The superscript numbers indicate which assumptions were violated; 1 = statistically extreme outlier, 2 = visible non-normality on QQ-plot, 3 = heteroscedasticity. For ANOVA, the F statistic (*F*), significance value (*p*), and estimated effect size (η^2) are presented. For the Tukey results the significance value (*p*) of each pairwise comparison is provided.

Parameter	Welch's ANOVA		Games-Howell		
	W	*p*	AY - IR	AY - MA	IR - MA
Torso Skin Volume	56.59	<0.001*	0.11	0.003*	<0.001*
Manus Skin Volume	38.55	<0.001*	0.001*	<0.001*	0.001*
TMT Skin Volume	82.15	<0.001*	0.047*	<0.001*	<0.001*
Hindlimb Length	101.28	<0.001*	<0.001*	0.804	<0.001*
Neck Length	124.88	<0.001*	<0.001*	0.021	<0.001*

Supplementary Table 5. ANOVA and Tukey HSD results for non-standardised (1st half, normal font), and standardised (2nd half, italics) parameters, whose raw data violated solely the homoscedasticity assumption of ANOVA. The table presents the Welch's ANOVA and Games-Howell post-hoc results, which do not assume similar variances across the breeds. This serves as a comparison to the with the results of the standard ANOVA. For Welch's ANOVA, the W statistic (*W*) and significance value (*p*) are presented. For the Tukey results the significance value (*p*) of each pairwise comparison is provided.

Supplementary Notes 2: Statistical comparisons of normalised segment sizes

Extended Results

Though breeds vary substantially in absolute proportions, both in their total and individual segment size (main text; Supplementary Notes 1), standardised segment lengths and volumes allow for direct comparisons between breeds, and relative differences inferred. In this section we provide a detailed overview of statistical analyses of standardised variants of the segments detailed in main text Figure 1.

ANOVA of the normalised linear and volumetric parameters found widespread differences between the three breeds, which tended to be more regional/localised than the non-normalised results, reflecting proportional differences between the ducks (Supplementary Figures 7-9; Supplementary Tables 6-8). We applied the same procedure for checking that test assumptions were met by our data, as was performed on the non-normalised data (Supplementary Notes 1), and found several parameters violated these conditions; shoulder width, gleno-acetabular distance, head hull volume, neck hull volume, forearm skin volume, and pes skin volume were found to include statistically extreme outliers; humerus length, shoulder width, gleno-acetabular distance, head hull volume, humerus skin volume, and pes skin volume possessed residuals which deviated substantially from normality (Supplementary Figures 10-12); and hindlimb length and neck length were shown to be heteroscedastic. We applied the same methods, as detailed for the non-normalised data in Supplementary Notes 1, to account for these anomalous parameters, and corrected ANOVA results are provided for each parameter in Supplementary Tables 4-5 (above). Comparison between these tests showed qualitative differences to several parameters following correction, neck length, shoulder width, head hull volume, humerus skin volume, and forearm skin volume, which are discussed below.

All normalised linear segment parameters differed significantly between at least two breeds (Supplementary Figure 7; Supplementary Table 6). Indian runners were found to bear the overall longest limbs of the three breeds, particularly in terms of the hindlimb where all segments were significantly longer than the other breeds. Mallard's were found to have reasonably long forelimbs, the distal segments (CMC, manus) were statistically comparable to the Indian runners, and all segments were significantly longer than the Aylesbury. The

mallards also exceeded the Aylesbury in their proximal hindlimb segments (thigh, shank). The non-appendicular parameters followed a similar trend (Supplementary Figure 7; Supplementary Table 6); Aylesbury's were the shortest in all parameters besides shoulder width, though this was no longer significant following correction (Supplementary Table 4); Indian runners were found to have longer necks and gleno-acetabular distances than the other breeds, and a longer sternum and wider hips than Aylesbury's; while mallards also have a longer sternum, wider hips, and a greater gleno-acetabular distance than Aylesbury's, though apparent differences in neck length do not survive correction (Supplementary Table 5).

Far fewer differences between the breeds were found in their normalised minimum convex hull segments, and those that did exist were concentrated in the forelimb (Supplementary Figure 8; Supplementary Table 7). Of the axial segments, only the head hull volume was found to significantly differ between the breeds, being larger in the mallards than the Indian runners (and the Aylesbury's following correction; Supplementary Table 4). The mallards were found to have proportionately the largest forelimbs, significantly differing for the other breeds in all parameters baring the Aylesbury in manus hull volume. The Indian runners had a significantly larger thigh and shank than the mallard, while the Aylesbury had a significantly larger pes hull volume than the other breeds.

The normalised skin segment values differed primarily between the mallards and the domesticated breeds, with few significant differences between Indian runners and Aylesbury's themselves (Supplementary Figure 9; Supplementary Table 8). There was no significant difference in head volume between the three breeds, but mallards had significantly larger torsos and smaller necks than the other breeds, which showed no statistical differences from one another in these parameters. Indian runners were found to have significantly smaller distal forelimb volumes than the other breeds (forearm, manus), and this was extended to the humerus when compared to mallards (though this was only once a correction was applied; Supplementary Table 4). Of the hindlimb parameters, the domesticated breeds did not differ significantly from one another, besides for pes volume, which was greater in the Aylesbury. The mallards were found to have the substantially lower hindlimb volumes than the other breeds (across all parameters).

There is a dichotomy between linear and volumetric proportions, similar to the findings of Supplementary Material 1. Indian runners have proportionately the longest skeleton (although

mallards are long in the forelimb), but have comparable skeletal and soft-tissue volumetric proportions to Aylesbury's (and are often smaller, particularly for the distal limbs; Supplementary Figures 8-9). This reinforces ideas suggested in Supplementary Notes 1, that a disproportionate increase in Aylesbury soft tissue has occurred without corresponding skeletal changes (at least in terms of the parameters investigated here). In contrast, mallards are consistent, having relatively long forelimbs, and thus relatively increased soft-tissue in those segments. Perhaps their biggest distinction with the domestic breeds is their relatively large torso, though this may be considered artefactual and resulting from the large necks and hindlimbs of the other breeds, which have lowered their relative torso size (the large forelimbs and hind limbs in Aylesbury's would further explain why they have the smallest relative torso.)

<u>Figures & Tables accompanying Supplementary Notes 2</u>

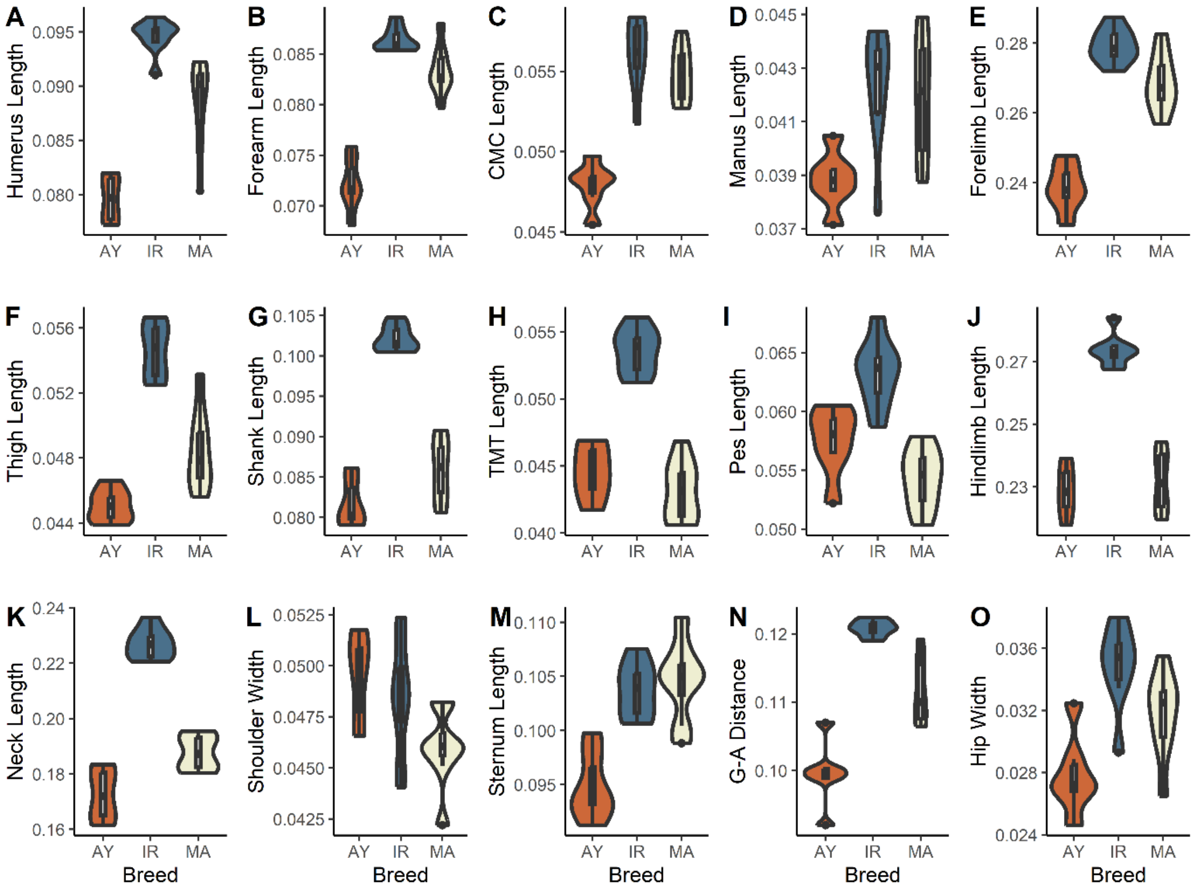

Supplementary Figure 7. Violin plots showing the primary statistics of each of the normalised linear measurements used in this analysis; **A-E**, forelimb parameters; **F-J**, hind limb parameters; **K-O**, non-appendicular parameters.

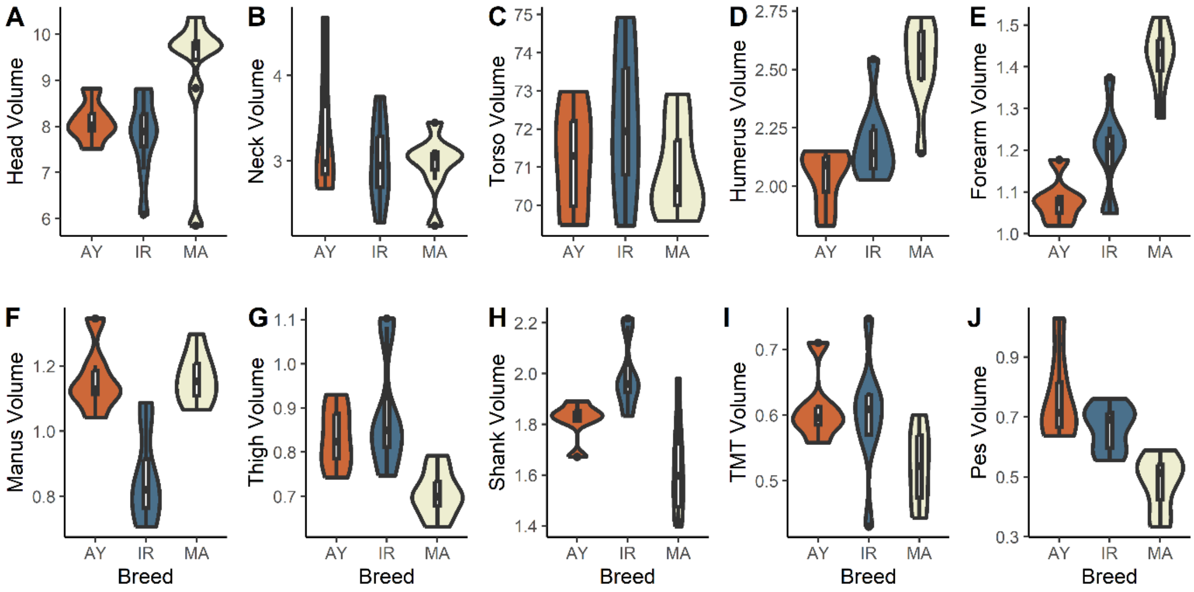

Supplementary Figure 8. Violin plots showing the primary statistics for each of the standardised minimum convex hull volumes used in this analysis; **A-C**, axial parameters; **D-F**, forelimb parameters; **G-J**, hind limb parameters.

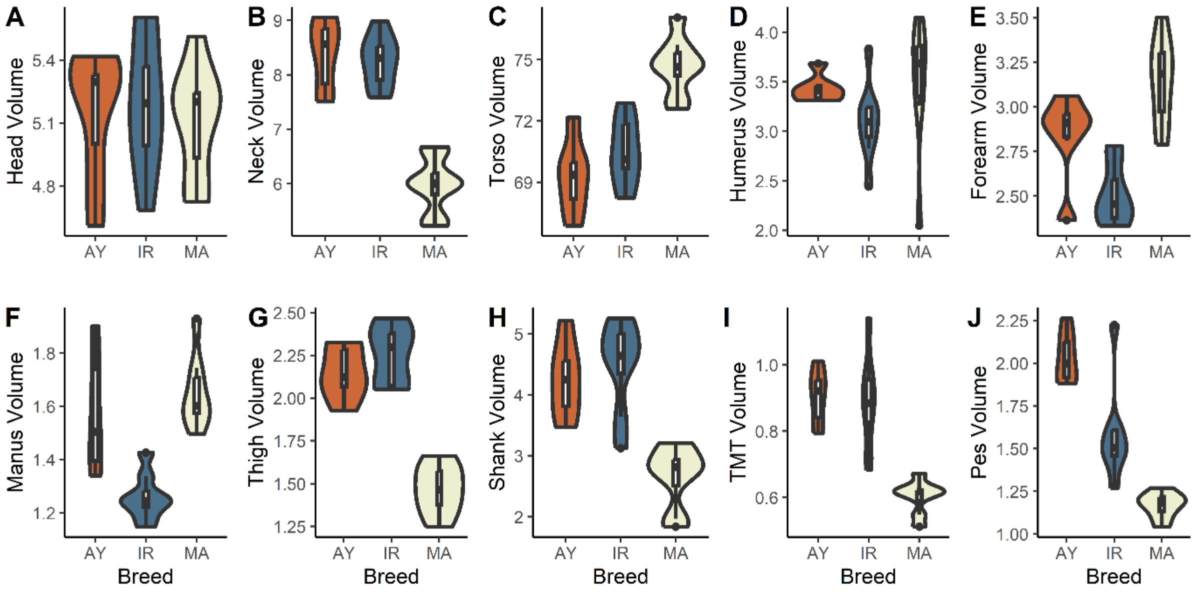

Supplementary Figure 9. Violin plots showing the primary statistics for each of the standardised final skin segment volumes used in this analysis; **A-C**, axial parameters; **D-F**, forelimb parameters; **G-J**, hind limb parameters.

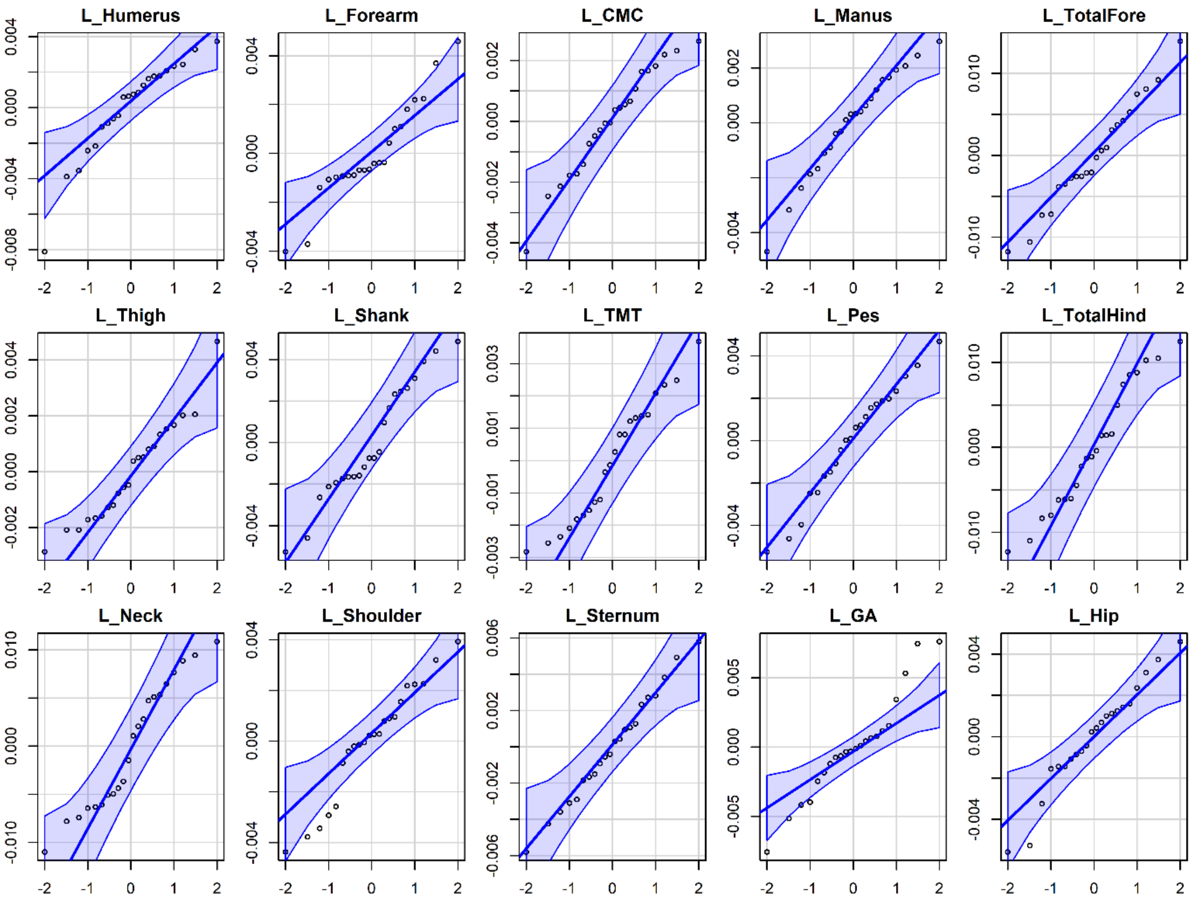

Supplementary Figure 10. QQ-plots showing the distribution of test residuals versus a normal distribution, for each of the standardised linear measurements. Normality was assessed by eye from these plots, on the condition that the datapoints should approximate the normal distribution (but can show some deviation, particularly towards the ends). We determined that humerus length, shoulder width, and gleno-acetabular distance contained non-normal data.

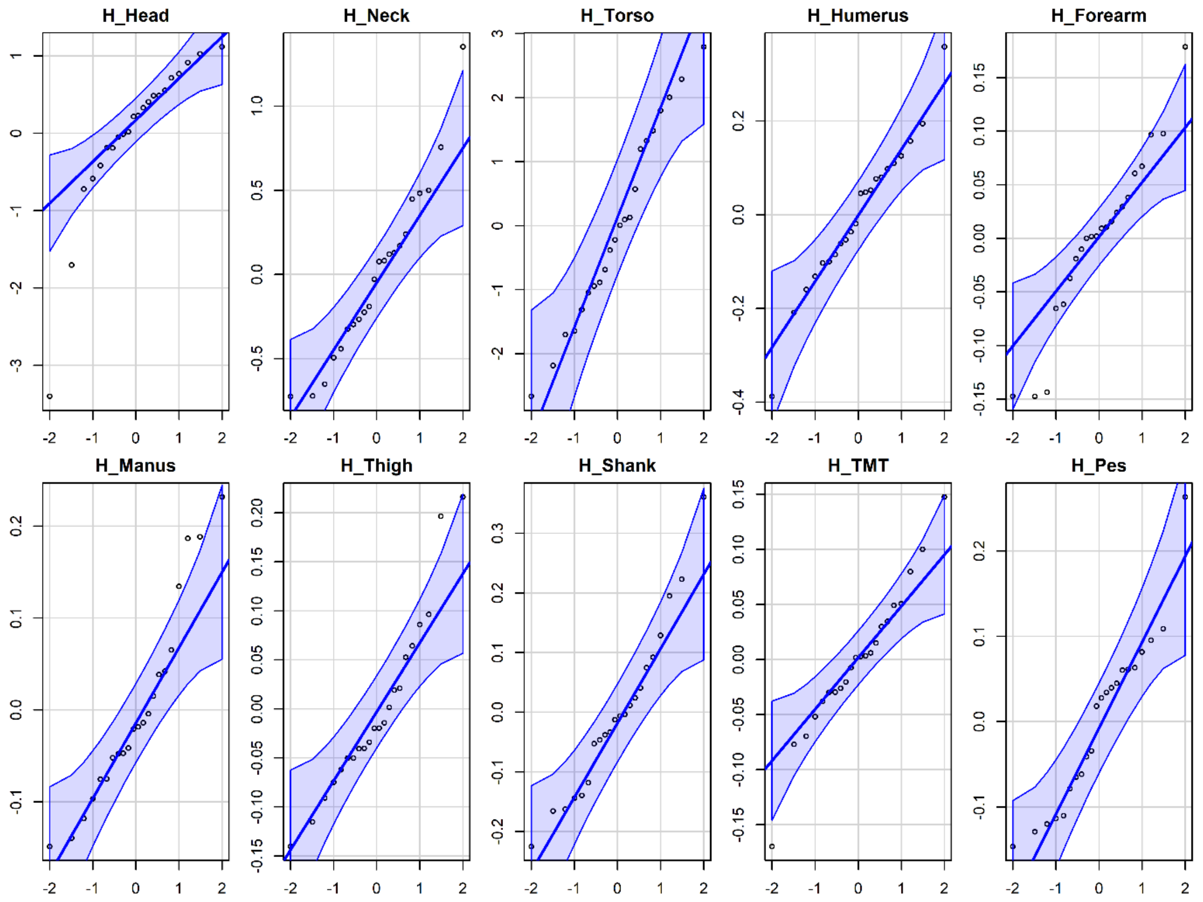

Supplementary Figure 11. QQ-plots showing the distribution of test residuals versus a normal distribution, for each of the standardised minimum convex hull volumes. Normality was assessed by eye from these plots, on the condition that the datapoints should approximate the normal distribution (but can show some deviation, particularly towards the ends). We determined that head hull volume contained non-normal data.

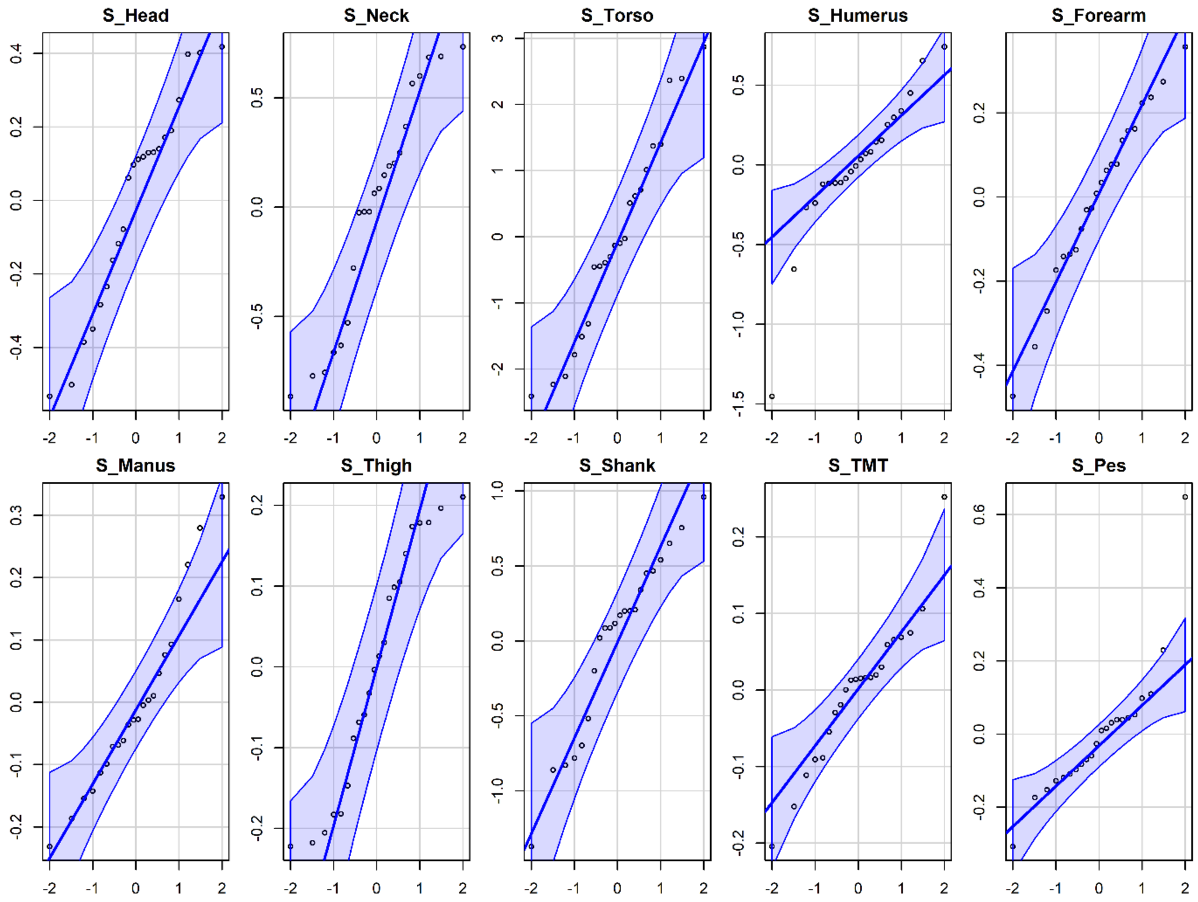

Supplementary Figure 12. QQ-plots showing the distribution of test residuals versus a normal distribution, for each of the standardised skin segment volumes. Normality was assessed by eye from these plots, on the condition that the datapoints should approximate the normal distribution (but can show some deviation, particularly towards the ends). We determined that the humerus and pes skin volumes contained non-normal data.

Parameter	ANOVA			Tukey HSD (*p*)		
	F	*p*	η^2	AY - IR	AY - MA	IR - MA
Humerus Length	45.381	<0.001*	0.83	<0.001*	<0.001*	0.001*
Forearm Length	80.72	<0.001*	0.90	<0.001*	<0.001*	0.027*
CMC Length	36.295	<0.001*	0.79	<0.001*	<0.001*	0.414
Manus Length	5.881	0.01*	0.38	0.013*	0.027*	0.926
Forelimb Length	63.982	<0.001*	0.87	<0.001*	<0.001*	0.014*
Thigh Length	45.862	<0.001*	0.83	<0.001*	0.01*	<0.001*
Shank Length	96.842	<0.001*	0.91	<0.001*	0.046*	<0.001*
TMT Length	63.846	<0.001*	0.87	<0.001*	0.408	<0.001*
Pes Length	21.359	<0.001*	0.69	0.003*	0.119	<0.001*
Hindlimb Length	77.95	<0.001*	0.89	<0.001*	0.757	<0.001*
Neck Length	118.534	<0.001*	0.93	<0.001*	0.002*	<0.001*
Shoulder Width	4.181	0.031*	0.31	0.691	0.033*	0.122
Sternum Length	19.347	<0.001*	0.67	<0.001*	<0.001*	0.836
GA Distance	51.318	<0.001*	0.84	<0.001*	<0.001*	<0.001*
Hip Width	11.577	0.001*	0.55	<0.001*	0.038*	0.079

Supplementary Table 6. ANOVA and Tukey HSD test results for all standardised (by body mass$^{0.33}$) linear segment lengths. For ANOVA, the F statistic (*F*), significance value (*p*), and estimated effect size (η^2) are presented, while N = 22 and D.F = 2. For the Tukey results the significance value (*p*) of each pairwise comparison is provided.

Parameter	ANOVA			Tukey HSD (p)		
	F	p	η^2	AY - IR	AY - MA	IR - MA
Head Volume	4.202	0.031*	0.31	0.848	0.134	0.031*
Neck Volume	0.919	0.416	0.09	0.5	0.439	0.993
Torso Volume	1.273	0.303	0.12	0.528	0.944	0.298
Humerus Volume	16.612	<0.001*	0.64	0.255	<0.001*	0.002*
Forearm Volume	29.928	<0.001*	0.76	0.052	<0.001*	<0.001*
Manus Volume	19.154	<0.001*	0.67	<0.001*	0.999	<0.001*
Thigh Volume	7.607	0.004*	0.45	0.56	0.055	0.003*
Shank Volume	12.704	<0.001*	0.57	0.09	0.063	<0.001*
TMT Volume	3.736	0.043*	0.28	0.961	0.068	0.082
Pes Volume	13.652	<0.001*	0.59	0.202	<0.001*	0.006*

Supplementary Table 7. ANOVA and Tukey HSD test results for all standardised (by total hull volume) minimum convex hull volumes. For ANOVA, the F statistic (F), significance value (p), and estimated effect size (η^2) are presented, while N = 22 and D.F = 2. For the Tukey results the significance value (p) of each pairwise comparison is provided.

Parameter	ANOVA			Tukey HSD (p)		
	F	p	η^2	AY - IR	AY - MA	IR - MA
Head Volume	0.126	0.883	0.01	0.965	0.976	0.872
Neck Volume	46.728	<0.001*	0.83	0.907	<0.001*	<0.001*
Torso Volume	23.129	<0.001*	0.71	0.378	<0.001*	<0.001*
Humerus Volume	1.64	0.22	0.15	0.399	0.965	0.228
Forearm Volume	16.929	<0.001*	0.64	0.03*	0.045*	<0.001*
Manus Volume	13.995	<0.001*	0.60	0.004*	0.613	<0.001*
Thigh Volume	57.89	<0.001*	0.86	0.427	<0.001*	<0.001*
Shank Volume	19.069	<0.001*	0.67	0.757	0.001*	<0.001*
TMT Volume	21.358	<0.001*	0.69	0.95	<0.001*	<0.001*
Pes Volume	33.746	<0.001*	0.78	0.001*	<0.001*	0.002*

Supplementary Table 8. ANOVA and Tukey HSD test results for all standardised (by total skin volume) skin segment volumes. For ANOVA, the F statistic (*F*), significance value (*p*), and estimated effect size (η^2) are presented, while N = 22 and D.F = 2. For the Tukey results the significance value (*p*) of each pairwise comparison is provided.

Supplementary Notes 3: Extended principal component analysis

Extended Results

PCA of hindlimb linear measurements only (Supplementary Figure 13), found all parameters to have a strong positive correlation with PC1 (> 87%), while PC2 showed a moderate positive correlation with pes length (46%), and a weaker negative correlation with femoral length (−32%). Mallards and Aylesbury ducks cluster in this morphospace due to similar PC1 scores. Differences between these two breeds primarily reside in terms of PC2, which corroborates the statistical results showing their divergence in the traits correlated with this component (Supplementary Figure 7). Indian runners are notably segregated from the other breeds, driven by more positive PC1 scores, consistent with bearing relatively longer hind limbs (Supplementary Figure 7). This breed tends to be neutrally scored in terms of PC2, reflecting the fact that all hindlimb segments are relatively elongate, and not specific segments as in the other breeds (Supplementary Figure 7).

Similar results were recovered for the PCA of forelimb only measurements (Supplementary Figure 14), where all parameters showed a strong positive correlation with PC1 (> 80%); but PC2 primarily reflected differences in just two parameters, in this case manus length (58%) and humeral length (−29%). However, the distribution of duck breeds in morphospace differed from the hindlimb analysis. Here, Indian runners and mallards were clustered by their PC1 scores, which are notably more positive than Aylesbury ducks, and indicative of proportionally longer forelimbs (the Indian runners being the longest; Supplementary Figure 7). Both Indian runners and mallards are noted for their variable PC2 scores, contrasting Aylesbury ducks which trended towards a neutral score on this component (Supplementary Figure 14). The highest scores on this component are held by mallards, which reflects the relatively long manus found in several members of this breed (also see Supplementary Figure 7). The Indian runner morphospace is exaggerated by a single aberrant individual with an unusually small manus (Supplementary Figure 14).

For the non-appendicular parameters (Supplementary Figure 15), neck length, sternum length, GA distance, and hip width were most strongly correlated with PC1 (> 76%), while shoulder width showed a weak negative correlation with this component (−16%). However,

shoulder width strongly correlated with PC2 (95%). Aylesbury ducks again occupy the negative end of PC1, while mallards and Indian runners were distributed at the positive end (Supplementary Figure 15). In terms of PC2, mallards tend to be more negatively scored, with Indian runners possessing the highest positive scores on this component. This morphospace shows that Indian runners have the longest necks, GA distance and widest hips, and that both domesticated breeds tend to have broader shoulders than mallards (Supplementary Figure 7).

PCA of the minimum convex hull volumes (Supplementary Figure 16), found PC1 to primarily reflect differences in fore and hind limb volumes. Hind limb hull volumes are moderately to strongly positively correlated with this component (60-77%), alongside neck volume and torso volume more moderately (20% and 36% respectively). Conversely, forelimb hull volumes display a moderate-high negative correlation (−50 to −82%), as does head volume (−67%). PC2 shows a strong positive correlation with neck volume (73%), and a moderate positive correlation with head, metatarsal, and pes volumes (48%, 34%, and 44%). Humerus, forearm, thigh, and shank volumes show little correlation (10% to −10%), while torso volume is strongly negatively correlated (−81%). Mallards score strongly negative on PC1, causing them to segregate from the domesticated breeds (Figure S3:4). This position reflects proportionally larger head and forelimb minimum convex hull volumes in the wild type compared to Aylesbury ducks and Indian runners, which have larger minimum convex hull volumes in the hindlimb, neck, and torso segments (Supplementary Figure 8). The primary distinction between the domestic breeds lies along PC2, where the more negative scores of Indian runners represent larger torso minimum convex hull volumes, and the Aylesbury duck's positive distribution reflects greater neck volumes (Supplementary Figure 8). However, there exists a moderate overlap in morphospace occupation between these breeds, indicating these trends are not entirely exclusive (Supplementary Figure 16).

Figures & Tables accompanying Supplementary Notes 3

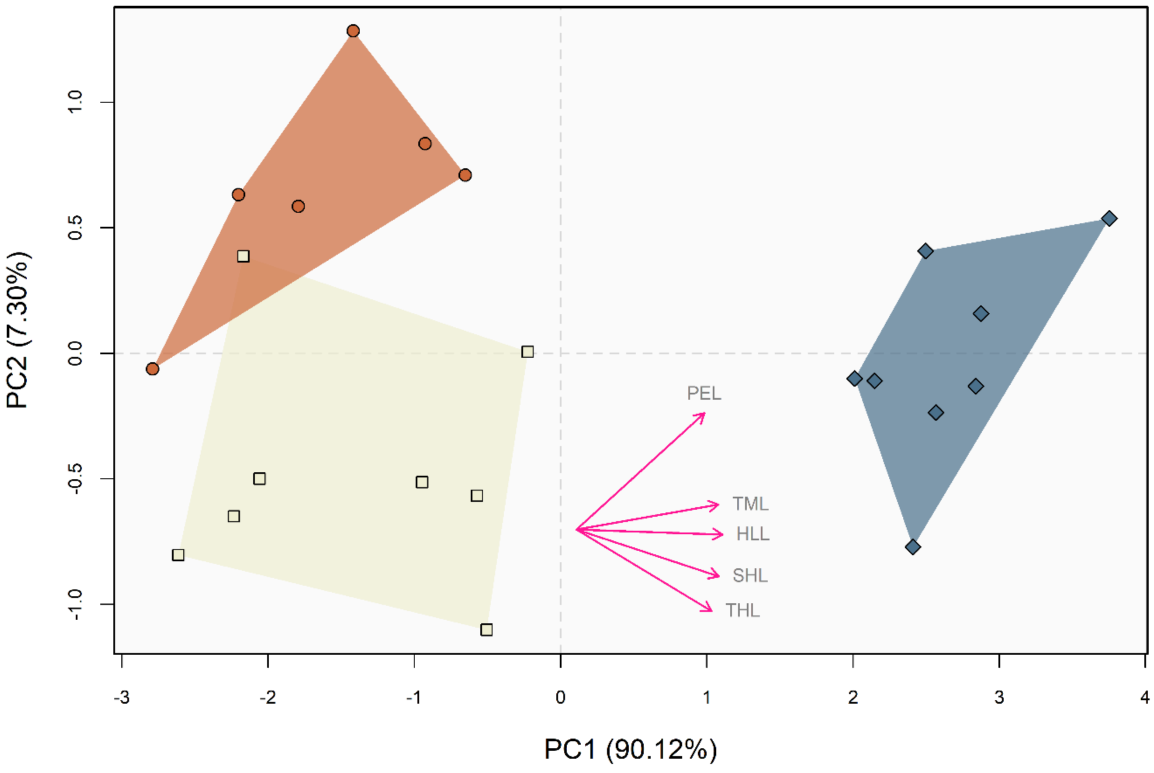

Supplementary Figure 13. PCA of hindlimb linear measurements, illustrating morphological differences between the three breeds. The biplot represents variable correlations with each component, note that it has been offset from 0,0 and increased in size (doubled), to improve legibility. FEL, femoral length; TIL, tibiotarsal length; TML, tarsometatarsal length; PEL, pes length; HLL, total hindlimb length.

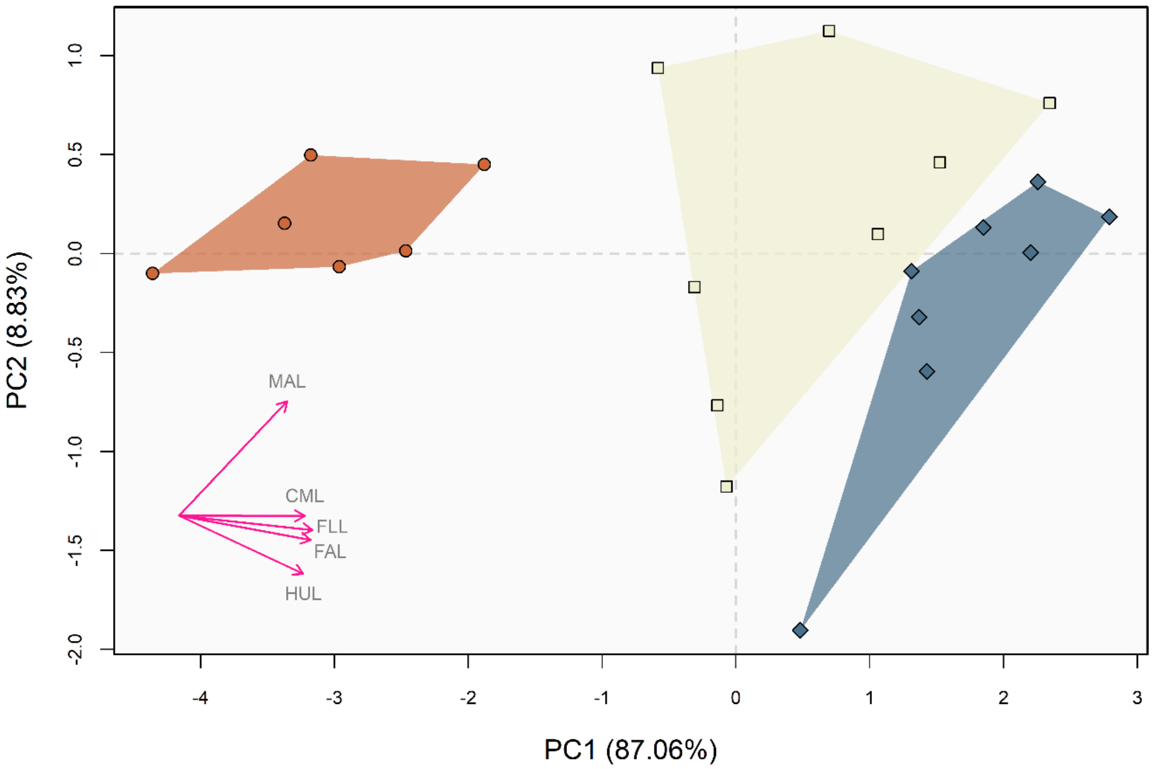

Supplementary Figure 14. PCA of forelimb linear measurements, illustrating morphological differences between the three breeds. The biplot represents variable correlations with each component, note that it has been offset from 0,0 and increased in size (doubled), to improve legibility. HUL, humeral length; FAL, forearm length; CML, carpometacarpal length; MAL, manus length; FLL, total forelimb length.

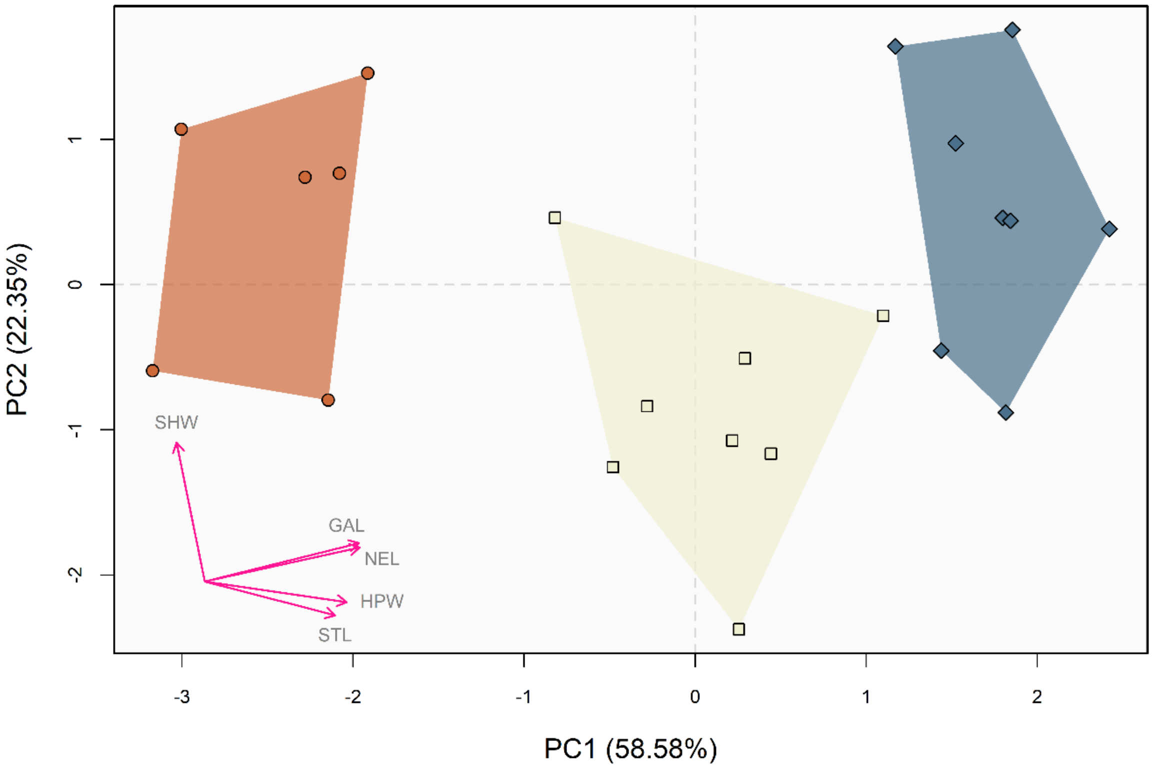

Supplementary Figure 15. PCA of non-appendicular linear measurements, illustrating morphological differences between the three breeds. The biplot represents variable correlations with each component, note that it has been offset from 0,0 and increased in size (doubled), to improve legibility. NEL, neck length; SHW, shoulder width; HPW, hip width; GAL, gleno-acetabular length; STL, sternal length.

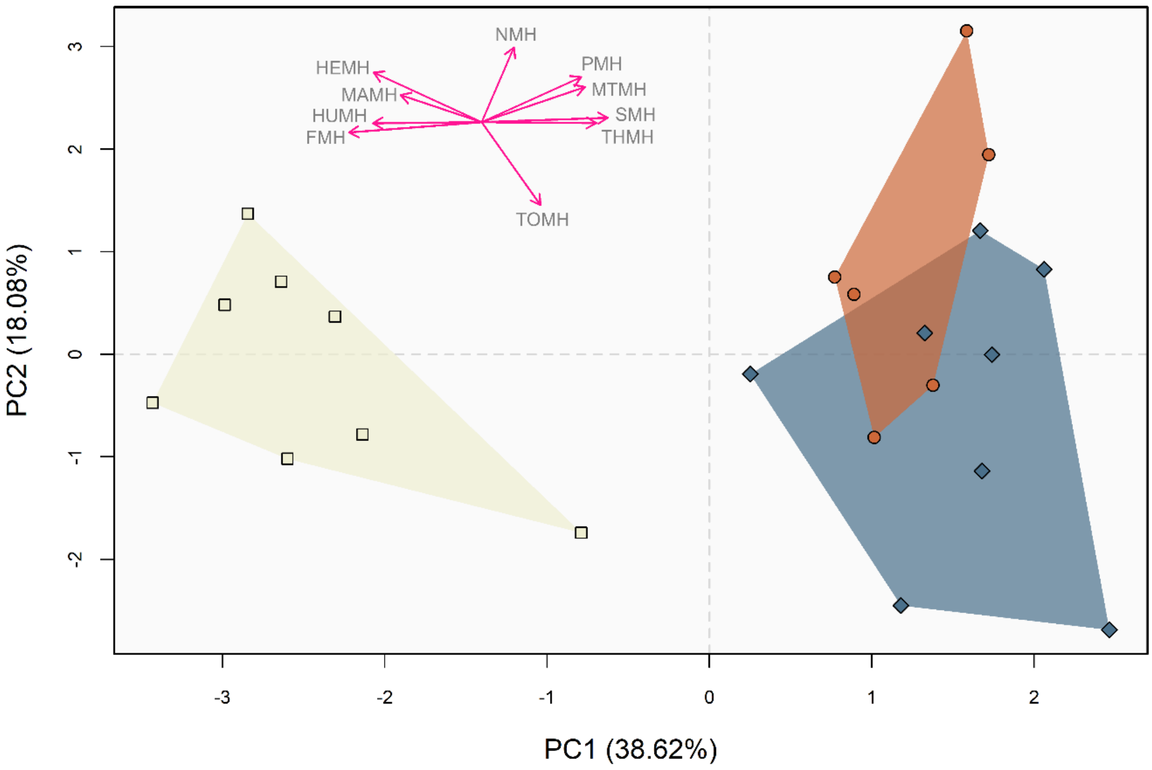

Supplementary Figure 16. PCA of the minimum convex hulls, illustrating volumetric morphological differences between the three breeds. Note that this measurement provides a fairer comparison of relative skeletal size than linear measurements (17). The biplot represents variable correlations with each component, note that it has been offset from 0,0 and increased in size (doubled), to improve legibility. HEMH, head hull volume; NMH, neck hull volume; TOMH, torso hull volume; HUMH, humeral hull volume; FMH, forearm hull volume; MAMH, manus hull volume; THMH, thigh hull volume; SMH, shank hull volume; MTMH, tarsometatarsal hull volume; PMH, pes hull volume.

Component	Eigenvalue	% variance
PC1	10.281	68.538
PC2	2.368	15.788
PC3	0.868	5.788
PC4	0.428	2.850
PC5	0.370	2.467
PC6	0.283	1.885
PC7	0.171	1.141
PC8	0.125	0.832
PC9	0.052	0.347
PC10	0.020	0.136
PC11	0.018	0.118
PC12	0.010	0.070
PC13	0.006	0.040
PC14	0.000	0.000
PC15	0.000	0.000

Supplementary Table 9. Eigenvalues and % variance for PCA of all linear measurements combined (Main text Figure 1A).

Component	Eigenvalue	% variance
PC1	5.56	55.58
PC2	2.08	20.77
PC3	0.89	8.88
PC4	0.61	6.13
PC5	0.42	4.17
PC6	0.21	2.14
PC7	0.11	1.13
PC8	0.08	0.78
PC9	0.04	0.42
PC10	0.00	0.00

Supplementary Table 10. Eigenvalues and % variance for PCA of the skin segment volumes (Main text Figure 1B).

Component	Eigenvalue	% variance
PC1	4.51	90.12
PC2	0.37	7.30
PC3	0.11	2.21
PC4	0.02	0.36
PC5	0.00	0.00

Supplementary Table 11. Eigenvalues and % variance for PCA of the hindlimb linear measurements (Supplementary Figure 13).

Component	Eigenvalue	% variance
PC1	4.35	87.06
PC2	0.44	8.83
PC3	0.18	3.51
PC4	0.03	0.60
PC5	0.00	0.00

Supplementary Table 12: Eigenvalues and % variance for PCA of the forelimb linear measurements (Supplementary Figure 14).

Component	Eigenvalue	% variance
PC1	2.929	58.576
PC2	1.117	22.349
PC3	0.522	10.434
PC4	0.297	5.934
PC5	0.135	2.707

Supplementary Table 13. Eigenvalues and % variance for PCA of the non-appendicular linear measurements (Supplementary Figure 15).

Component	Eigenvalue	% variance
PC1	3.86	38.62
PC2	1.81	18.08
PC3	1.46	14.57
PC4	0.96	9.62
PC5	0.76	7.64
PC6	0.54	5.41
PC7	0.26	2.62
PC8	0.17	1.72
PC9	0.10	0.97
PC10	0.07	0.75

Supplementary Table 14. Eigenvalues and % variance for PCA of the minimum convex hull volumes (Supplementary Figure 16).

Parameter	PC1	PC2
Humerus Length	0.932771	-0.18488
Forearm Length	0.925141	-0.33962
CMC Length	0.855866	-0.28831
Manus Length	0.696142	-0.2953
Forelimb Length	0.945964	-0.28918
Thigh Length	0.942098	0.131362
Shank Length	0.946314	0.257633
TMT Length	0.824099	0.513832
Pes Length	0.638631	0.699487
Hindlimb Length	0.907632	0.40166
Neck Length	0.93921	0.183817
Shoulder Width	0.036055	0.77573
Sternum Length	0.688631	-0.52229
GA Distance	0.912957	-0.06214
Hip Width	0.746898	-0.23292

Supplementary Table 15. Variable correlations with PC1 and PC2 from the all linear measurements combined analysis (main text Figure 1A).

Parameter	PC1	PC2
Head Volume	0.240058	0.48505
Neck Volume	0.906051	-0.03039
Torso Volume	-0.90506	-0.37552
Humeral Volume	-0.16587	0.738278
Forearm Volume	-0.66788	0.613934
Manus Volume	-0.4895	0.791992
Thigh Volume	0.94197	-0.00727
Shank Volume	0.889014	0.043932
Tarsometatarsal Volume	0.92376	0.240977
Pes Volume	0.785249	0.301288

Supplementary Table 16. Variable correlations with PC1 and PC2 from the skin segment volume analysis (main text Figure 1B).

Parameter	PC1	PC2
Thigh Length	0.923385	-0.32494
Shank Length	0.973432	-0.18636
TMT Length	0.970839	0.09924
Pes Length	0.874276	0.463318
Hindlimb Length	0.999515	-0.01997

Supplementary Table 17. Variable correlations with PC1 and PC2 from the hindlimb linear measurement analysis (Supplementary Figure 13).

Parameter	PC1	PC2
Humerus Length	0.92702	-0.29413
Forearm Length	0.982565	-0.12311
CMC Length	0.939101	-0.00081
Manus Length	0.807715	0.578435
Forelimb Length	0.997059	-0.07304

Supplementary Table 18. Variable correlations with PC1 and PC2 from the forelimb linear measurement analysis (Supplementary Figure 14).

Parameter	PC1	PC2
Neck Length	0.901223	0.265372
Shoulder Width	-0.16538	0.958046
Sternum Length	0.762323	-0.23198
GA Distance	0.905081	0.23554
Hip Width	0.830017	-0.14104

Supplementary Table 19. Variable correlations with PC1 and PC2 from the non-appendicular linear measurement analysis (Supplementary Figure 15).

Parameter	PC1	PC2
Head MHV	-0.66567	0.483867
Neck MHV	0.197869	0.726475
Torso MHV	0.362154	-0.80864
Humeral MHV	-0.67082	-0.01134
Forearm MHV	-0.81823	-0.10072
Manus MHV	-0.49939	0.264921
Thigh MHV	0.705866	-0.00858
Shank MHV	0.776624	0.042635
Tarsometatarsal MHV	0.635916	0.342261
Pes MHV	0.61175	0.439351

Supplementary Table 20. Variable correlations with PC1 and PC2 for the minimum convex hull analysis (Supplementary Figure 16).

Supplementary Notes 4: Raw energetics and spatiotemporal kinematics

Extended results

$\mathrm{Mass-specific}\ P_{\mathrm{met}}$ increased linearly with U (Supplementary Figure 17A; Supplementary Table 21). The $\mathrm{mass\text{–}specific}\ P_{\mathrm{met}}$ rate of increase in the Aylesbury was ~2.6-fold and ~1.4-fold the rate of the Indian runners and the mallards, respectively. $\mathrm{Mass\text{–}specific}\ P_{\mathrm{met}}$ was comparable at the lowest U in all breeds but diverged with increasing U (Supplementary Figure 17A). The higher $\mathrm{mass\text{–}specific}\ P_{\mathrm{met}}$ in the Aylesbury ducks compared to Indian runners and mallards, indicates that fast walking in the Aylesbury requires a proportionately higher metabolic rate. The relationship between $\mathrm{net\text{–}}P_{\mathrm{met}}$ and U had a similar pattern to $\mathrm{mass\text{–}specific}\ P_{\mathrm{met}}$, $\mathrm{net\text{–}}P_{\mathrm{met}}$ was greater in the Aylesbury ducks and lower in the Indian runners at the highest speed. However, standing metabolic rate was comparable between breeds (mallard = 6.21 ± 1.59, Indian runners = 6.55 ± 0.99, Aylesbury ducks = 6.38 ± 0.85; Kruskal-Wallis test; χ^2= 0.58; df = 2; p = 0.75). COT decreased curvilinearly with U (Supplementary Figure 17C; Supplementary Table 21) and all breeds shared a common slope and intercept. Similarly, net COT showed a mild negative linear relation with U (Supplementary Figure 17D; Supplementary Table 21) and again a common slope and intercept between the three breeds was found.

l_{stride} increased curvilinearly with increasing U. The rate of increase was steepest in Indian runners and Aylesbury ducks (Supplementary Figure 17E; Supplementary Table 21). l_{stride} appears to converge at the slowest speed (0.28 ms^{-1}) in all breeds, however, Aylesbury and Indian runners use relatively longer strides at higher speeds (Supplementary Figure 17E). f_{stride} increased linearly with increasing U, although at a higher rate in the mallards compared to the other two breeds (Supplementary Figure 17F; Supplementary Table 21). Curvilinear negative models were found between t_{stance} and U in all breeds (Supplementary Figure 17G; Supplementary Table 21). Compared to the others, the mallard had shorter t_{stance}, and this decreased with U at the fastest rate (Supplementary Figure 17G; Supplementary Table 21). t_{swing} is independent of U (Supplementary Figure 17G; Supplementary Table 21). Indian runner ducks have the longest t_{swing}, whilst mallards have the shortest. Finally, DF decreased linearly with U. None of the three breeds shared a common slope or intercept. DF in Aylesbury ducks decreased at the fastest rate, whilst in Indian runner it decreased at the slowest rate (Supplementary Figure 17H; Supplementary Table 21).

Figures & Tables accompanying Supplementary Notes 4

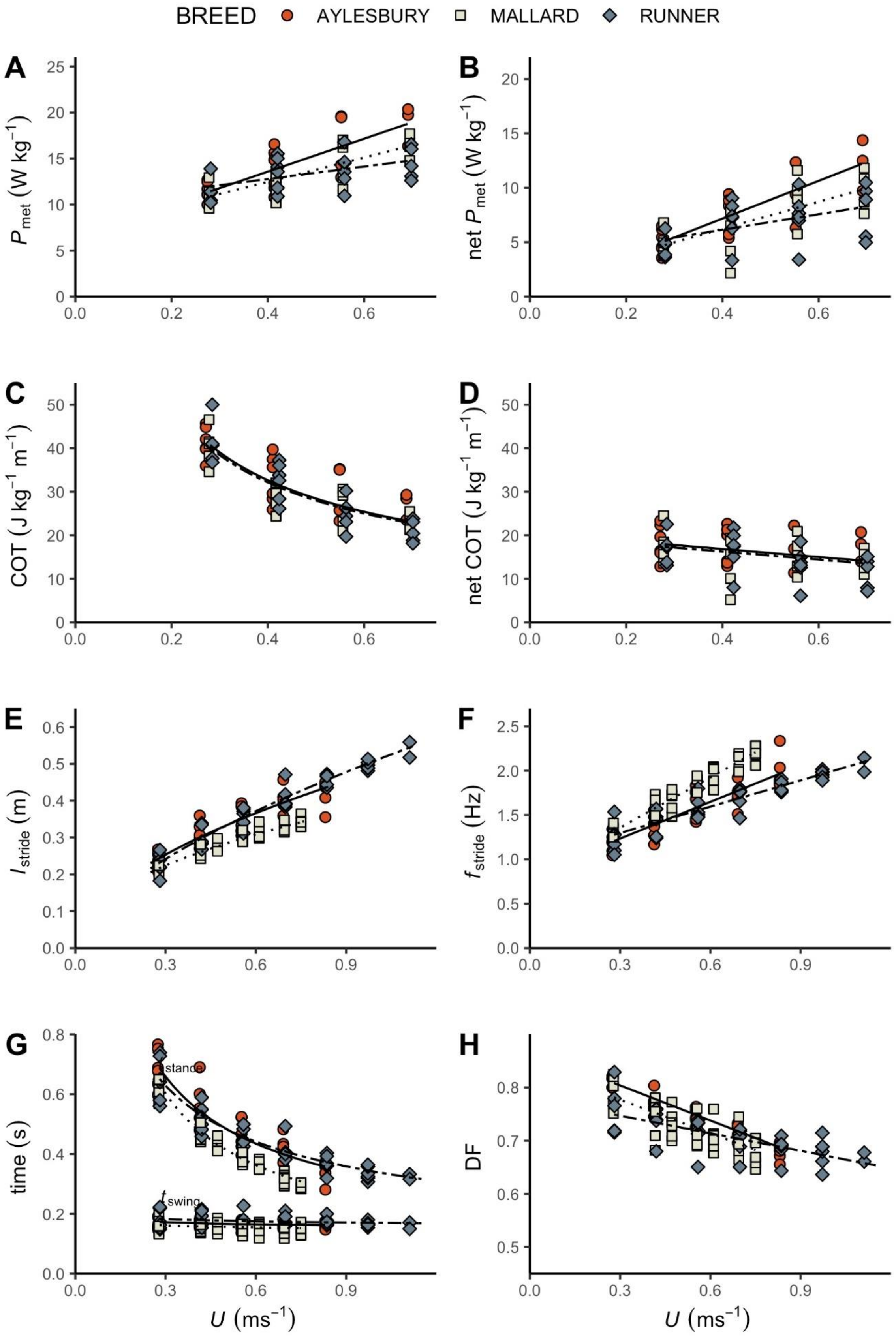

Supplementary Figure 17. Absolute (i.e. body size non-adjusted) energetics and kinematics of treadmill locomotion. *P*met (**A**) and net *P*met (**B**) increased linearly with U in all breeds with the highest rate of increase found in Aylesbury ducks. COT (**C**) and net COT (**D**) decreased with U and no differences were detected between breeds. Stride length (**E**) increased curvilinearly with speed, with the highest rate of increase in Indian runners. Stride frequency (**F**) increased linearly with U, with the greatest rate of increase found in mallards. Stance duration (**G**) decreased with speed in all breeds, and at the fastest decrease in mallards. No significant effect of speed or breed on swing duration was found (**G**). DF decreased linearly with U (**H**), with the fastest decline in Aylesburys and the slowest in Indian runners. Aylesbury (red circles, solid line), mallard (white squares, dotted line) and Indian runner (blue diamonds, dot-dash line).

Parameter	Final model	r^2	$n_p{}^2$	Equations
Energetics				
P_{met}	U ($F_{1,56}$ = 12.54; P <0.001 **)	0.72	0.18	IR: $19.60\ U + 4.15$
	Breed ($F_{2,56}$ = 69.33; P < 0.001 **)		0.71	MA: $19.60\ U$ +13.97
				AL: $19.60\ U + 23.76$
mass specific P_{met}	U ($F_{1,54}$ = 55.73; P <0.001 **)	0.53	0.51	IR: $6.76\ U + 10.07$
	Breed ($F_{2,54}$ = 4.00; P = 0.023 *)		0.13	MA: $13.22\ U$ +7.17
	U × Breed ($F_{2,54}$ = 4.19; P = 0.020*)		0.13	AL: $17.87\ U + 6.43$
net P_{met}	U ($F_{1,54}$ = 46.68; P <0.001 **)	0.49	0.46	IR: $7.15\ U + 3.28$
	Breed($F_{2,54}$ = 3.89; P = 0.026 *)		0.13	MA: $12.42\ U + 1.26$
	U × Breed ($F_{2,54}$ = 3.06; P = 0.055)		0.10	AL: $17.35\ U + 0.23$
$\log_{10}$ COT	$\log_{10} U$ ($F_{1,58}$ = 130.38; P <0.001 **)	0.69	–	IR: $18.33\ U^{-0.62}$
				MA: $18.33\ U^{-0.62}$
				AL: $18.33\ U^{-0.62}$
net COT	U ($F_{1,58}$ = 6.16; P = 0.233)	0.08	–	IR: $-9.17\ U + 20.21$
				MA: $-9.17\ U + 20.21$
				AL: $-9.17\ U + 20.21$
Spatiotemporal Kinematics				
$\log_{10} l_{\text{stride}}$	$\log_{10} U$ ($F_{1,103}$ = 1040.25; P < 0.001 **)	0.91	0.90	IR: $0.51\ U^{0.62}$
	Breed ($F_{2,103}$ = 54.98; P < 0.001 **)		0.53	MA: $0.39\ U^{0.46}$
	$\log_{10} U$ × Breed ($F_{2,103}$ = 6.234; P = 0.003*)		0.11	AL: $0.47\ U^{0.53}$

f_{stride}	U ($F_{1, 103}$ = 467.02; P < 0.001 **)	0.85	0.81	IR: $1.00\ U + 0.99$
	Breed ($F_{2, 103}$ = 71.49; P < 0.001 **)		0.58	MA: $1.89\ U + 0.79$
	$U \times$ Breed ($F_{2, 103}$ = 20.04; P < 0.001**)		0.28	AL: $1.37\ U + 0.82$
$\log_{10} t_{\text{stance}}$	$\log_{10} U$ ($F_{1, 103}$ = 8287; P < 0.001 **)	0.90	0.89	IR: $0.34\ U^{-0.51}$
	Breed ($F_{2, 103}$ = 65.93; P < 0.001 **)		0.56	MA: $0.25\ U^{-0.73}$
	$\log_{10} U \times$ Breed ($F_{2, 103}$ = 9.82; P < 0.001**)		0.16	AL: $0.32\ U^{-0.60}$
$\log_{10} t_{\text{swing}}$	$\log_{10} U$ ($F_{1, 105}$ = 1.441; P = 233)	0.25	0.01	IR: $0.17\ U^{-0.06}$
	Breed ($F_{2, 105}$ = 18.713; P < 0.001 **)		0.26	MA: $0.15\ U^{-0.06}$
				AL: $0.16\ U^{-0.06}$
DF	U ($F_{1, 103}$ = 190.92; P < 0.001 **)	0.67	0.65	IR: $-0.11\ U + 0.78$
	Breed ($F_{2, 103}$ = 8.21; P < 0.001 **)		0.14	MA: $-0.21\ U + 0.84$
	$U \times$ Breed ($F_{2, 103}$ = 10.32; P < 0.001**)		17	AL: $-0.22\ U + 0.87$

Supplementary Table 21: Model results for the non-normalised (absolute speed; *U*) energetic and spatiotemporal kinematic parameters. This comprises ANCOVA results for each studied parameter, identifying breed-specific differences across the studied speed range.

Supplementary Notes 5: Comparative Analysis of Avian Cost of Transport

Extended Methods

An underlying assumption of using ducks as a model of avian locomotion is that they experience comparable physiological demands to other birds during walking, and thus exhibit similar locomotor CoT to other birds of a similar body size. Some authors have argued that 'waddling', which is common to Mallards and Aylesburys, but not Indian runners (2-3), is a relatively costly gait (4). Studies of other waddling birds (cormorants) have found they experience similar CoT to parasagittal walkers of comparable body sizes (5); however, CoT is substantially increased in broiler chickens (6), which also waddle and share morphological characteristics with Aylesburys (7).

Here, we have collated minimum CoT of walking birds from the literature (5-6,8-14), comprising 13 birds total (11 distinct taxa, and three breeds of chicken). Where net− CoT instead of CoT was provided, CoT was back calculated using available speed and resting metabolic rate information (Table S8:1). Minimum CoT and body mass were first converted to logarithmic scale (base 10), and linear regression was performed in R to estimate correlation coefficients for three separate models. The first analysis (Model 1) was performed on all birds except for the ducks and is useful for testing the relative economy of ducks versus other birds. For the second analysis (Model 2), the three duck breeds were included, which allows us to produce a new bird-specific scaling equation for minimum walking CoT, that can be compared against previous work on birds and mammals (8). Lastly, we performed a phylogenetic least squares regression (Model 3) using an avian phylogeny modified from Jetz *et al*. (9) and a measure of phylogenetic signal (λ) assuming a Brownian model of trait evolution, as an alternative bird-specific equation that accounts for phylogenetic covariance. Note that the broiler was excluded from all statistical analyses because of its extremely high CoT, which greatly exceeds all other birds.

Extended Results & Discussion

All three models showed a strong negative allometric relationship between minimum walking CoT and body mass in birds, similar to previous studies of avian and mammalian locomotion (Supplementary Figure 18; 5,8). Model 1 is described by the equation $y = 1.391x^{-0.456}$, and minimum CoT for all three duck breeds are found to sit within the 95% confidence intervals of this line, indicating that duck CoT is within the region of similarly sized birds. Indeed,

despite both having been selectively bred for meat, the Aylesbury duck does not have the exceptionally elevated CoT found in broiler chickens.

Given the similarity between ducks and other birds, it is unsurprising that incorporating them into the bird-specific minimum CoT scaling analysis has minimal impact upon the equation; Model 2: $y = 1.392x^{-0.454}$. Compared to the general animal (bird and mammal) minimum CoT allometric analysis of Rubenson *et al*., (8); the coefficient is higher (Log10(17.8) = 1.25), indicative of a general trend of relatively higher minimum costs in birds compared to mammals; and the exponent is steeper ($^{-0.471}$), demonstrating that cost decreases more slowly with increasing body mass in birds. It is additionally worth noting that Rubenson *et al*., (8) also included a duck in their original analysis and found it to have amongst the highest minimum costs in their dataset. While this may be partly explained by the differences in bird-specific scaling outlined above, the value they used (31.03 J kg^{-1} m^{-1}) is also higher than those reported here (Supplementary Table 22).

The allometric equation for Model 3 is $y = 1.368x^{-0.441}$, and therefore quite similar to Models 1 & 2. PGLS will downweigh the residuals of taxa with more shared evolutionary history (i.e. longer shared branches), however the measure of phylogenetic signal in these residuals is low ($\lambda = -0.075$), and only a small ‘correction’ is applied to the model. The marginally negative λ, demonstrates that closely related taxa tend to be more dissimilar than expected, and this may predominantly reflect the densely sampled galloanserans (50% of all taxa), which diverge comparatively recently in our phylogeny, but also show marked differences in CoT (Supplementary Figure 18; Supplementary Table 22). While PGLS allows the impact of phylogenetic processes upon trait relationships to be evaluated, its predictive utility is (often) limited. For example, the birds included here are overwhelmingly either predominantly terrestrial (Ratites, Galliformes, Marabou), or semiaquatic (Anseriformes, Moorhen), or a combination thereof (Penguins). Therefore, attempting to predict the minimum CoT of a bird that exists outside this relatively narrow functional and phylogenetic sample (e.g. a passerine), would likely yield less reliable results than Model 2.

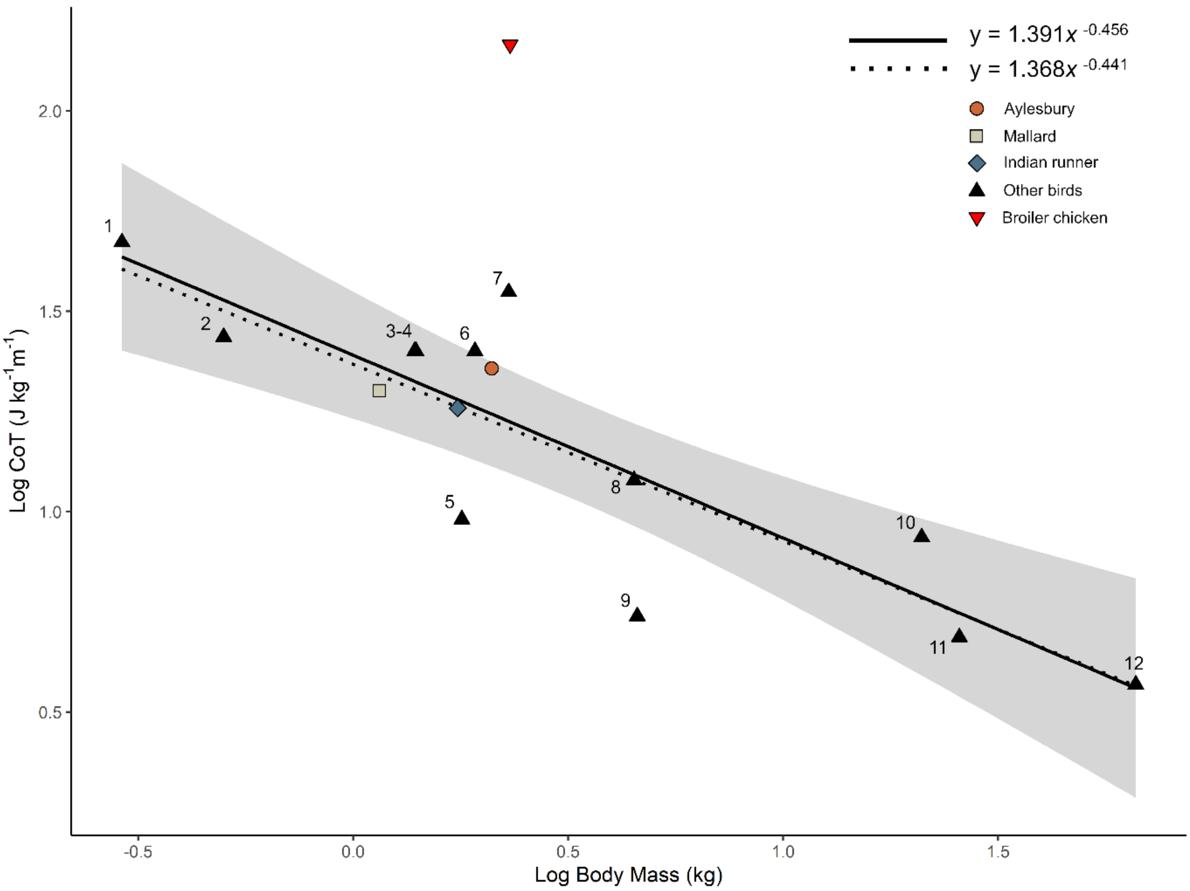

Supplementary Figure 18: Log-log plot of body against the minimum walking CoT in birds. The solid line with 95% confidence intervals corresponds to Model 1, while the dotted line without confidence intervals represents Model 3. Model 2 was not presented given its similarity to Model 1, and the equation is presented directly in the text above. ‘Other birds’ are numbered as follows; 1, Moorhen; 2, Svalbard Rock Ptarmigan; 3, Leghorn Chicken (Bantam); 4, Guineafowl; 5, Barnacle Goose; 6, Leghorn Chicken (Regular); 7, Great Cormorant; 8, Marabou Stork; 9, Peafowl; 10, Emperor Penguin; 11, Emu; 12, Ostrich. Also, as stated directly above, the broiler was not included in any analysis and is plotted here for comparative purposes only.

Common Name	Taxon	BM	Log(BM)	CoT	Log(CoT)	Reference
Ostrich	*Struthio camelus*	66.1	1.820	3.706	0.569	8
Emu	*Dromaius novaehollandiae*	25.7	1.410	4.866	0.687	10
Leghorn (large)	*Gallus domesticus*	1.92	0.283	25.172	1.401	13
Leghorn (bantam)	*Gallus domesticus*	1.39	0.143	25.172	1.401	13
Broiler	*Gallus domesticus*	2.317	0.365	146.435	2.166	6
Ptarmigan	*Lagopus muta hyperborea*	0.5	-0.301	27.335	1.437	14
Guineafowl	*Numida meleagris*	1.4	0.146	25.173	1.401	12
Peafowl	*Pavo cristatus*	4.58	0.661	5.488	0.739	15
Barnacle Goose	*Branta leucopsis*	1.79	0.253	9.581	0.981	11
Mallard	*Anas platyrhynchos*	1.15	0.061	20.054	1.302	New
Aylesbury	*Anas domesticus*	2.1	0.322	22.788	1.358	New
Indian Runner	*Anas domesticus*	1.75	0.243	18.148	1.259	New
Emperor Penguin	*Aptenodytes forsteri*	21	1.322	8.64	0.937	16
Great Cormorant	*Phalacrocorax carbo*	2.3	0.362	35.364	1.549	5
Moorhen	*Gallinula sp.*	0.29	-0.538	47.072	1.673	8
Marabou Stork	*Leptoptilos crumenifer*	4.5	0.653	11.995	1.079	8

Supplementary Table 22: Minimum CoT and body mass data for walking birds. BM is given in kg, and CoT is given in J kg^{-1} m^{-1}, log-transformed versions are base 10.

Supplementary Notes 6: Statistical analysis of joint angles and trunk pitch for discrete stride parameters

Extended Methods

For each accepted stride (see main text), we extracted specific flexion-extension (FE) and trunk pitch angles for five parameters; Minimum FE/pitch, Maximum FE/pitch, toe-on FE/pitch, toe-off FE/pitch and Mid-stance FE/pitch. We then performed statistical analysis on each of these parameters, comparing them across breeds. Generally, the parameters violated the normality and homogeneity of variance assumptions required by ANOVA, thus, analysis was undertaken using the Kruskal-Wallis, with *post-hoc* Dunn's test to identify which breeds significantly differed from one another. During preliminary analysis, we found that several parameters had multimodal and/or exaggerated distributions, often in association with statistical outliers. We therefore present here the raw data (in boxplots; Supplementary Figures 19-22). as well as the data used in the statistical analysis, which were cropped to remove those outliers (in violin plots; Supplementary Figures 23-26).

Extended Results

Hip Joint Angle. There is considerable overlap between all three breeds in their hip kinematics (Supplementary Figures 19 & 23), though many of the studied parameters are found to significantly differ between them in post-hoc analysis (Supplementary Table 23). The domesticated breeds are capable of higher degrees of hip extension than mallards (Supplementary Figure 19A; Supplementary Table 23), with Aylesburys tending to have the lowest flexion values overall (Supplementary Figure 19B; Supplementary Table 23). This trend extends to specific points in stride, as the domestic breeds have lower hip flexion at both toe-on (Supplementary Figure 19C; Supplementary Table 23; though differences between Mallards and Indian runners are not significant), toe-off (Supplementary Figure 19D; Supplementary Table 23), and midstance (Supplementary Figure 19E; Supplementary Table 23). The domestic breeds also show significant differences, with the Aylesbury more flexed at toe-on (Supplementary Figure 19C; Supplementary Table 23), and the runner at toe-off (Supplementary Figure 19D; Supplementary Table 23).

Knee Joint Angle. Aylesbury's are found to operate the most extended knee joints, (Supplementary Figure 24A; Supplementary Table 24), whilst Indian runners utilise significantly higher maximum knee flexion angles than the other breeds (Supplementary Figure 24B; Supplementary Table 24). Aylesbury's have the most extended knees at toe-on, but the most flexed knees at toe-off (though the latter does not significantly differ from Mallards, Supplementary Figure 24C-D; Supplementary Table 24). Conversely, Indian runners are more flexed than other breeds at toe-on (though again this does not significantly differ from Mallards), and more extended at toe-off (Supplementary Figure 24C-D; Supplementary Table 24). Mallards were found to use significantly more flexed knees at midstance than the domestic breeds (Supplementary Figure 24E; Supplementary Table 24).

Ankle Joint Angle. Only minor differences were found in terms of minimum ankle flexion, with Indian runners capable of greater ankle extension than Mallards (Supplementary Figure 25A; Supplementary Table 25). However, in terms of maximum flexion, Indian runners were found to be significantly more flexed than the other breeds, which did not significantly differ from one another (Supplementary Figure 25B; Supplementary Table 25). Analysis of the stance phase parameters showed widespread differences between the breeds (Supplementary Figure 25C-E; Supplementary Table 25), where Indian runners always operated the most extended ankle, with Aylesburys being the second most extended at toe-on, while Mallards were the second most extended at toe-off and midstance.

Trunk Pitch. In contrast with the joint kinematics, we find a clear trend across breeds whereby the Indian runners always have the greatest pitch (and often substantially greater pitch), while the mallards and Aylesburys have much lower pitch and overlap one another (Supplementary Figures 22 & 26). The higher trunk pitch of the Indian runner is always significant, whereas any marginal differences between Aylesburys and mallards are not statistically supported (Supplementary Table 26).

Figures & Tables accompanying Supplementary Notes 6

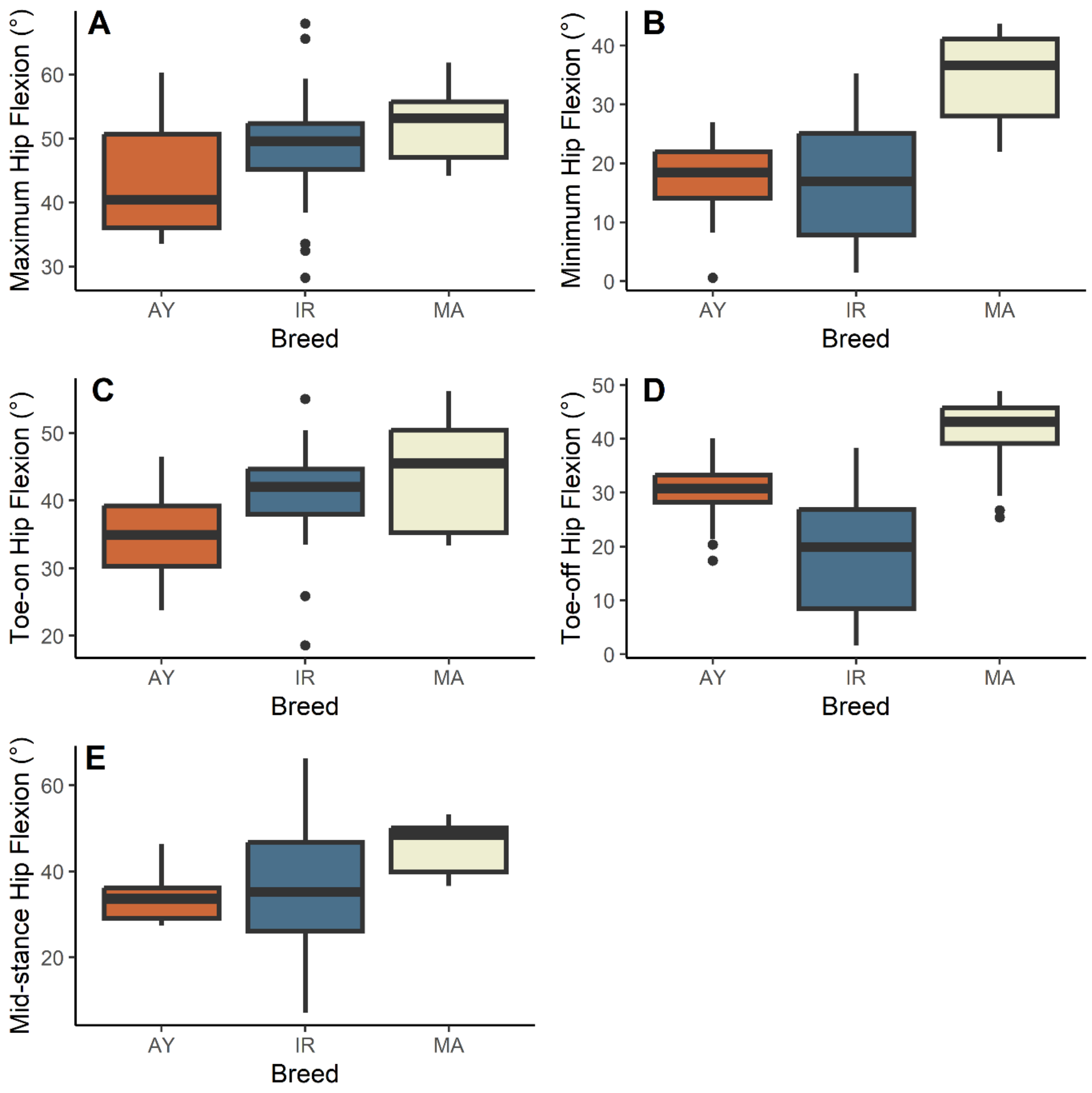

Supplementary Figure 19. Boxplots comparing hip flexion-extension angles between the duck breeds for specific, discrete stride parameters; **A**, maximum FE angle; **B**, minimum FE angle; **C**, FE angle at toe-on; **D**, FE angle at toe-off; **E**, mid-stance FE angle. Note that these plots show the raw data, before outliers were removed for statistical analysis (see Figure S5:5 below).

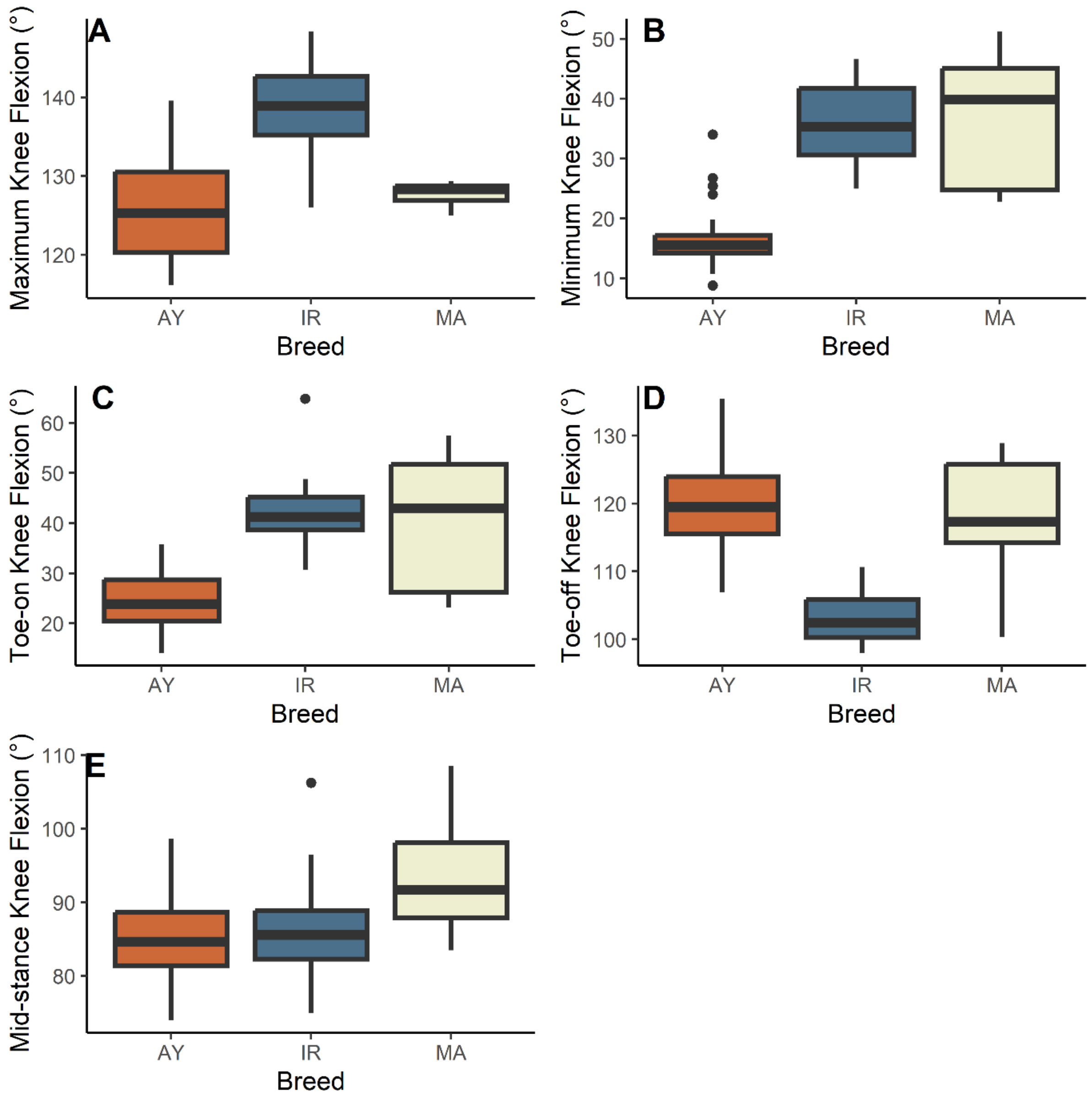

Supplementary Figure 20. Boxplots comparing knee flexion-extension angles between the duck breeds for specific, discrete stride parameters; **A**, minimum FE angle; **B**, maximum FE angle; **C**, FE angle at toe-on; **D**, FE angle at toe-off; **E**, mid-stance FE angle. Note that these plots show the raw data, before outliers were removed for statistical analysis (see Figure S5:6 below).

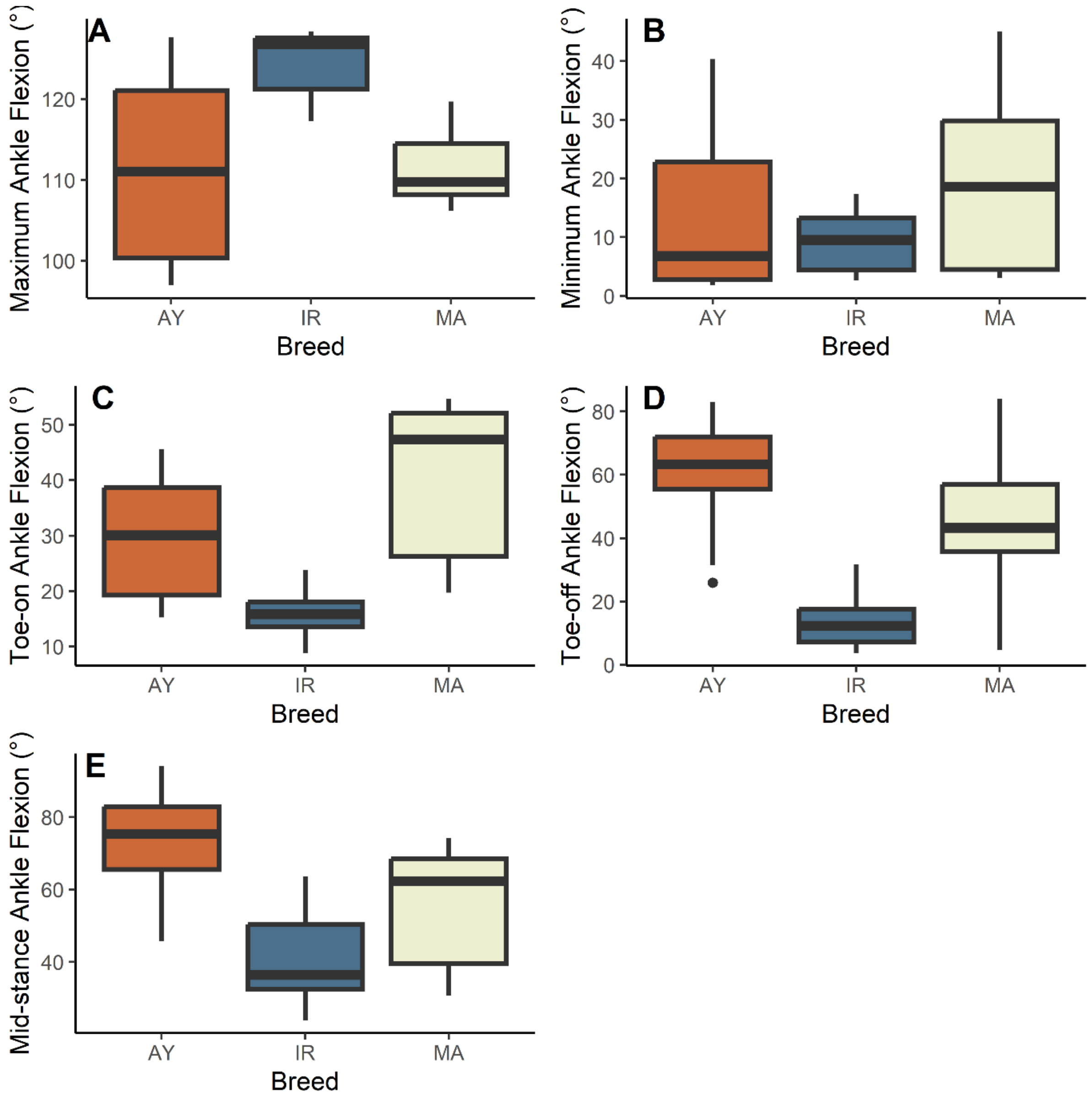

Supplementary Figure 21. Boxplots comparing ankle flexion-extension angles between the duck breeds for specific, discrete stride parameters; **A**, minimum FE angle; **B**, maximum FE angle; **C**, FE angle at toe-on; **D**, FE angle at toe-off; **E**, mid-stance FE angle. Note that these plots show the raw data, before outliers were removed for statistical analysis (see Figure S5:7 below).

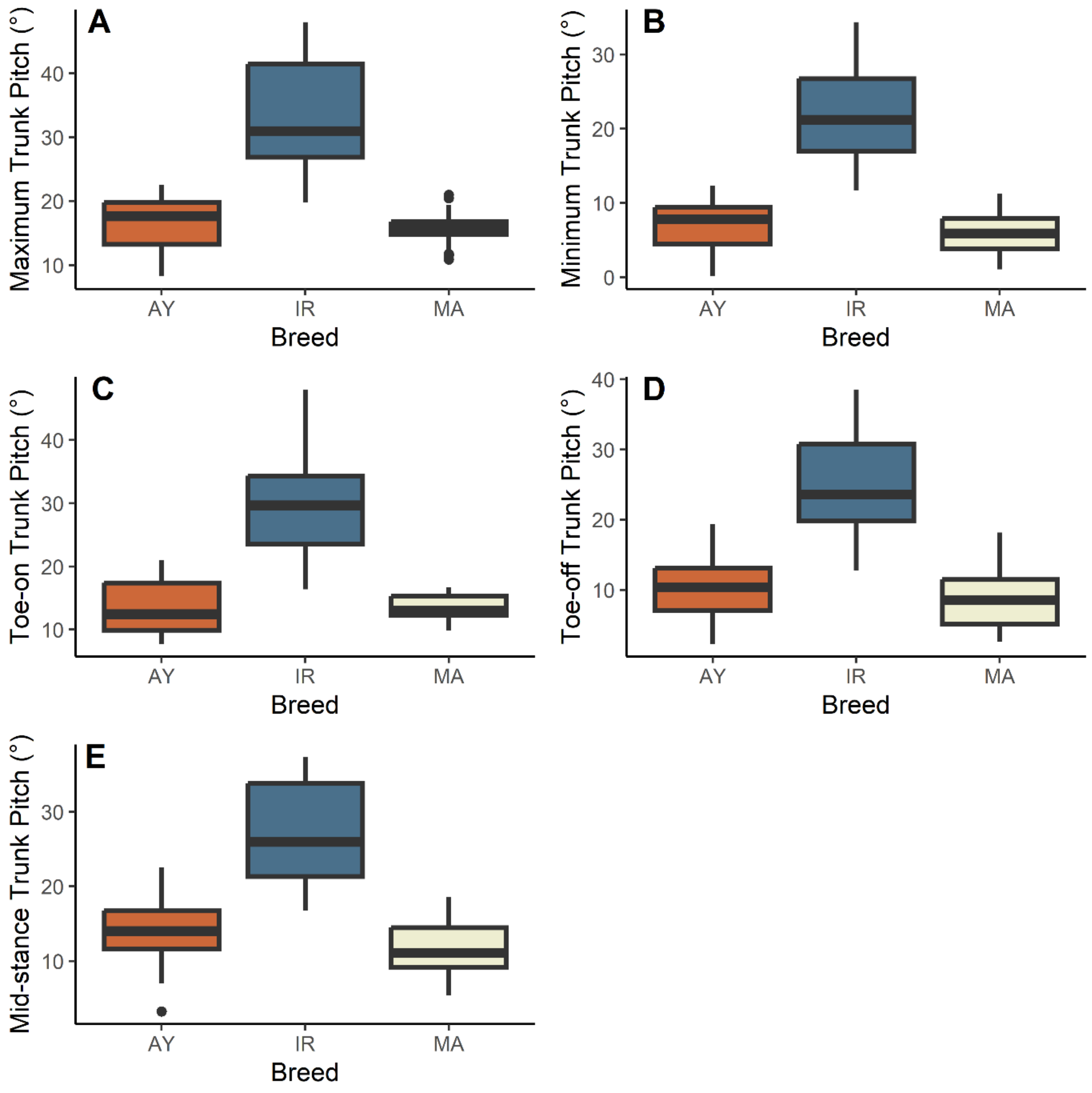

Supplementary Figure 22. Boxplots comparing trunk pitch between the duck breeds for specific, discrete stride parameters; **A**, minimum pitch; **B**, maximum pitch; **C**, pitch at toe-on; **D**, pitch at toe-off; **E**, mid-stance pitch. Note that these plots show the raw data, before outliers were removed for statistical analysis (see Figure S5:8 below).

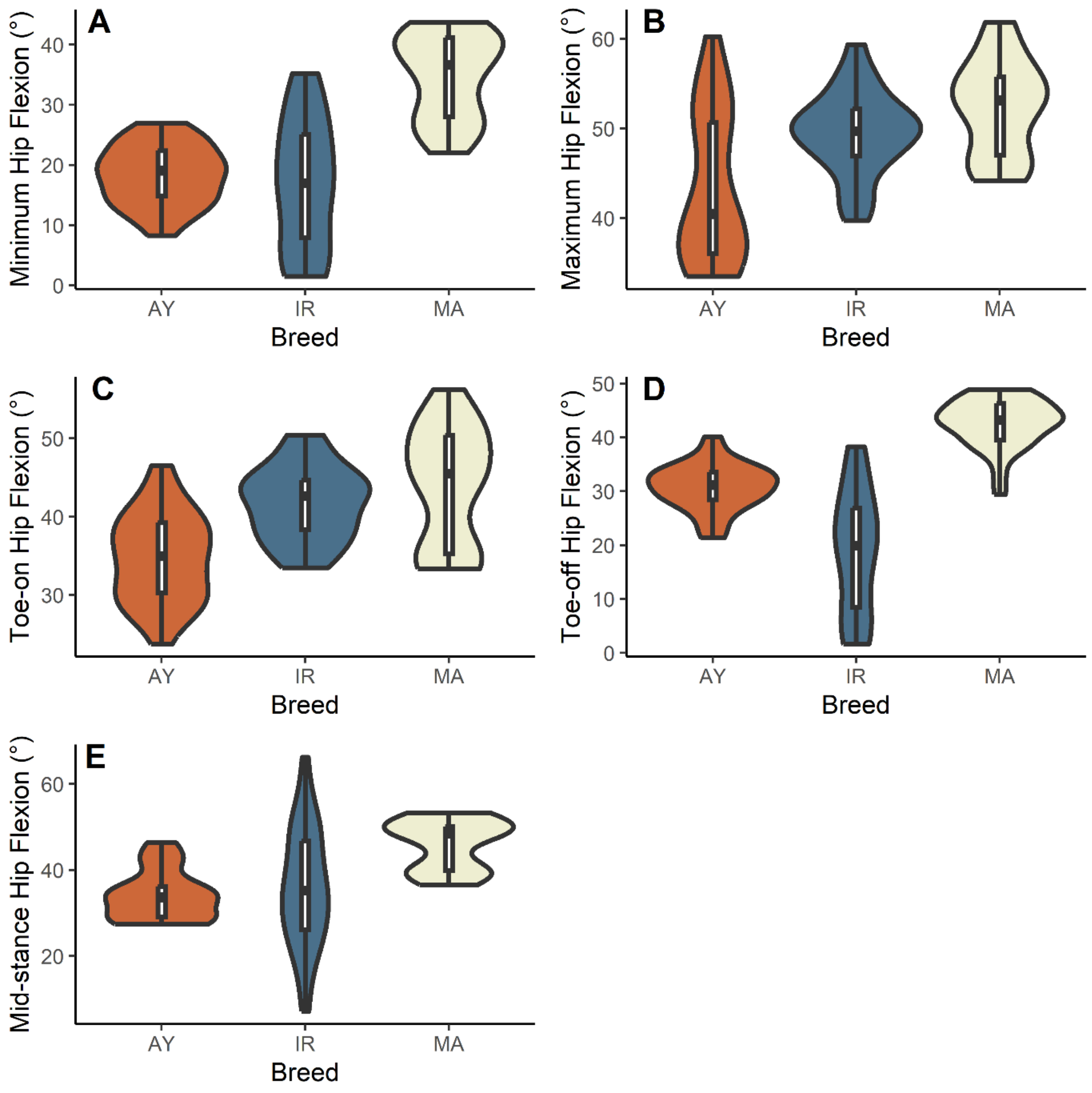

Supplementary Figure 23. Violin plots comparing hip flexion-extension angles between the duck breeds for specific, discrete stride parameters; **A**, minimum FE angle; **B**, maximum FE angle; **C**, FE angle at toe-on; **D**, FE angle at toe-off; **E**, mid-stance FE angle. The data presented here has been cropped of statistical outliers and the probability densities illustrate the resulting distribution.

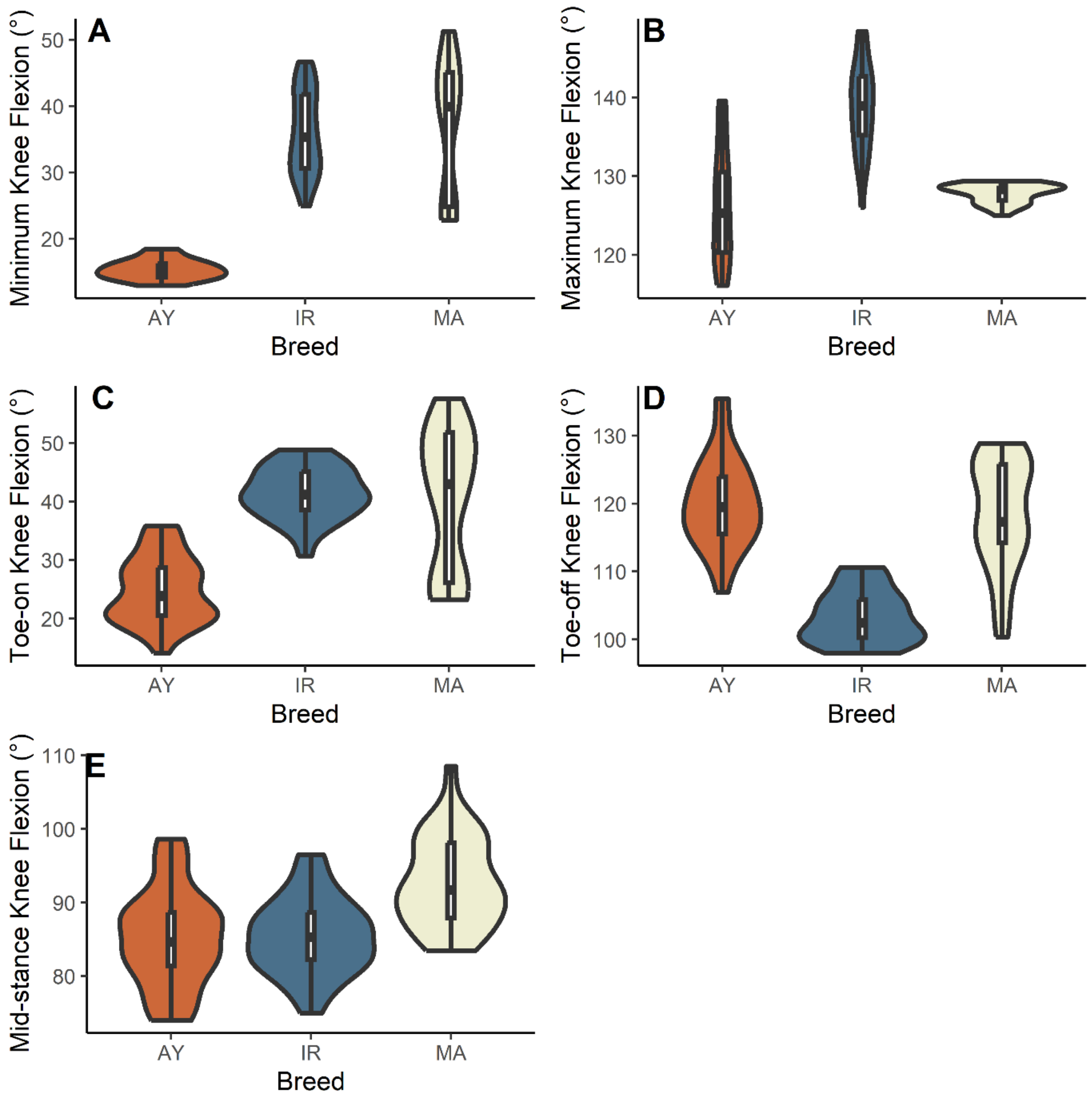

Supplementary Figure 24. Violin plots comparing knee flexion-extension angles between the duck breeds for specific, discrete stride parameters; **A**, minimum FE angle; **B**, maximum FE angle; **C**, FE angle at toe-on; **D**, FE angle at toe-off; **E**, mid-stance FE angle. The data presented here has been cropped of statistical outliers and the probability densities illustrate the resulting distribution.

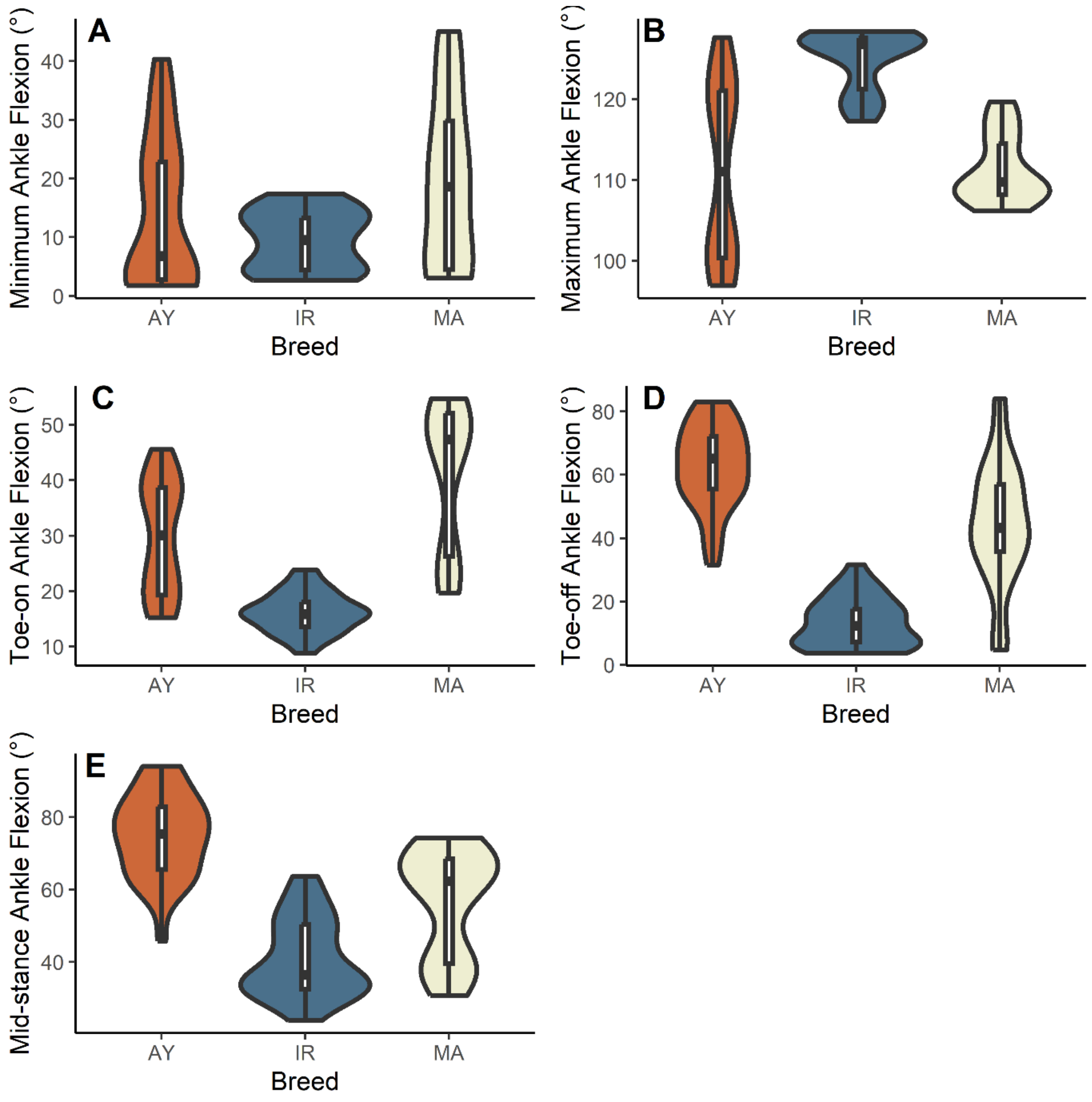

Supplementary Figure 25. Violin plots comparing ankle flexion-extension angles between the duck breeds for specific, discrete stride parameters; **A**, minimum FE angle; **B**, maximum FE angle; **C**, FE angle at toe-on; **D**, FE angle at toe-off; **E**, mid-stance FE angle. The data presented here has been cropped of statistical outliers and the probability densities illustrate the resulting distribution.

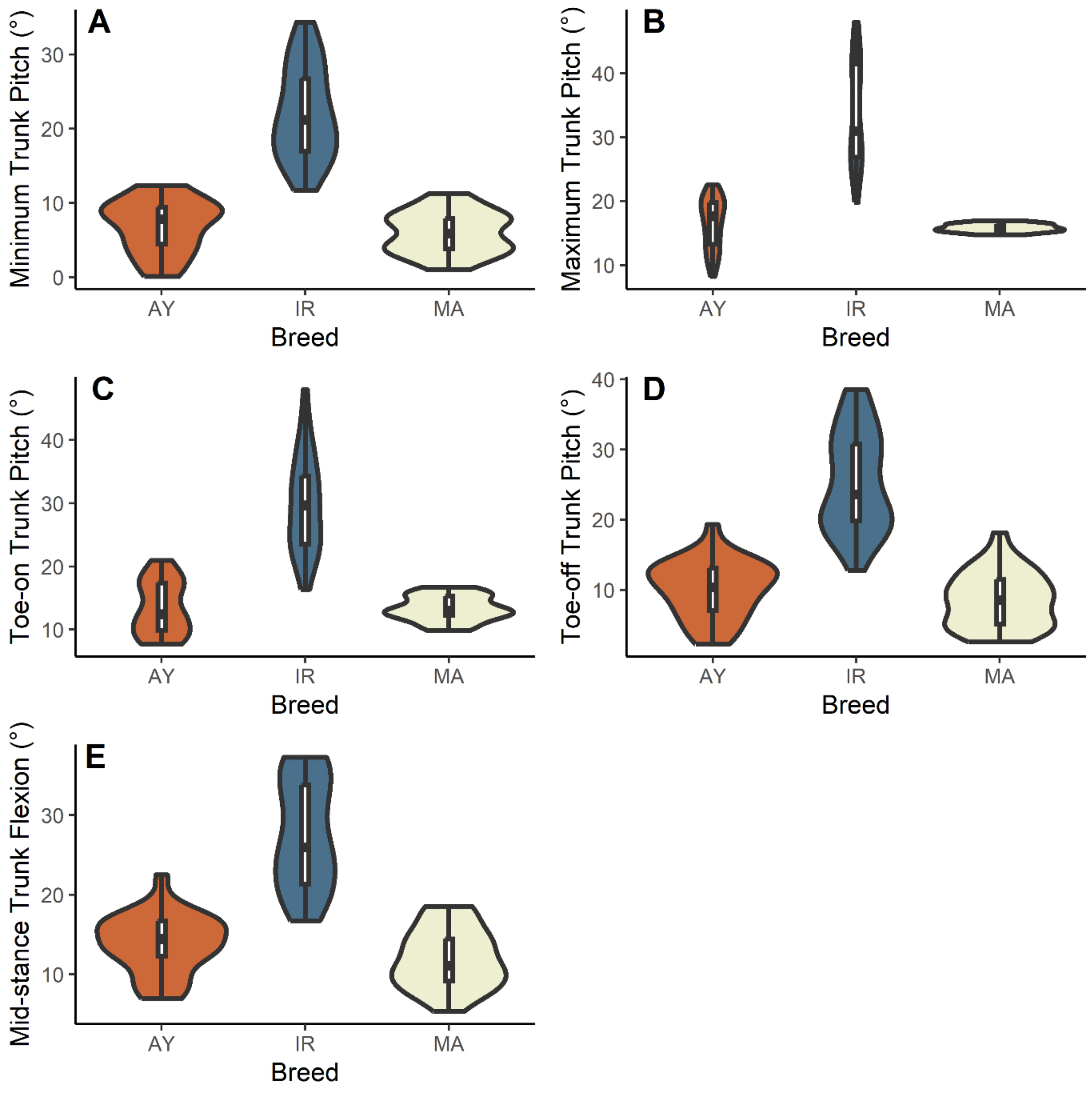

Supplementary Figure 26. Violin plots comparing trunk pitch between the duck breeds for specific, discrete stride parameters; **A**, minimum pitch; **B**, maximum pitch; **C**, pitch at toe-on; **D**, pitch at toe-off; **E**, mid-stance pitch. The data presented here has been cropped of statistical outliers and the probability densities illustrate the resulting distribution.

Parameter	N	Statistic	Df	*P*	Effect size	Dunn's Results		
						AY - IR	AY - MA	IR - MA
Minimum Flexion	89	46.669	2	<0.001	0.519	ns	****	****
Maximum Flexion	84	18.877	2	<0.001	0.208	*	****	ns
Toe-on Flexion	87	24.187	2	<0.001	0.264	***	****	ns
Toe-off Flexion	86	58.825	2	<0.001	0.685	**	****	****
Mid-Stance Flexion	90	29.057	2	<0.001	0.311	ns	****	***

Supplementary Table 23. Kruskal-Wallis and Dunn's test results for the hip joint flexion-extension angles. Note that N may vary between parameters as each was cropped for outliers individually. Dunn's results are given as either 'ns' for not significant, or * for significant (where * number indicates a higher significance value).

Parameter	N	Statistic	Df	*P*	Effect size	Dunn's Results		
						AY - IR	AY - MA	IR - MA
Minimum Flexion	83	49.573	2	<0.001	0.595	****	****	ns
Maximum Flexion	90	45.919	2	<0.001	0.505	****	ns	****
Toe-on Flexion	89	40.767	2	<0.001	0.451	****	****	ns
Toe-off Flexion	90	51.681	2	<0.001	0.571	****	ns	****
Mid-Stance Flexion	89	23.374	2	<0.001	0.249	ns	****	****

Supplementary Table 24. Kruskal-Wallis and Dunn's test results for the knee joint flexion-extension angles. Note that N may vary between parameters as each was cropped for outliers individually. Dunn's results are given as either 'ns' for not significant, or * for significant (where * number indicates a higher significance value).

Parameter	N	Statistic	Df	*P*	Effect size	Dunn's Results		
						AY - IR	AY - MA	IR - MA
Minimum Flexion	90	7.175	2	0.028	0.059	ns	ns	*
Maximum Flexion	90	44.882	2	<0.001	0.493	****	ns	****
Toe-on Flexion	90	53.449	2	<0.001	0.591	****	*	****
Toe-off Flexion	89	57.506	2	<0.001	0.645	****	*	****
Mid-Stance Flexion	90	51.514	2	<0.001	0.569	****	***	**

Supplementary Table 25. Kruskal-Wallis and Dunn's test results for the ankle joint flexion-extension angles. Note that N may vary between parameters as each was cropped for outliers individually. Dunn's results are given as either 'ns' for not significant, or * for significant (where * number indicates a higher significance value).

Parameter	N	Statistic	Df	*P*	Effect size	Dunn's Results		
						AY - IR	AY - MA	IR - MA
Minimum Pitch	90	59.764	2	<0.001	0.664	****	ns	****
Maximum Pitch	76	53.074	2	<0.001	0.700	****	ns	****
Toe-on Pitch	90	57.394	2	<0.001	0.637	****	ns	****
Toe-off Pitch	90	57.379	2	<0.001	0.637	****	ns	****
Mid-Stance Pitch	89	56.985	2	<0.001	0.639	****	ns	****

Supplementary Table 26. Kruskal-Wallis and Dunn's test results for the trunk pitch. Note that N may vary between parameters as each was cropped for outliers individually. Dunn's results are given as either 'ns' for not significant, or * for significant (where * number indicates a higher significance value).

Supplementary Notes 7: Positional relationships of the pes and centre of mass

Extended Methods

To calculate the positional relationship between CoM and the pes, we exported the following time-series data from our inverse kinematics; whole-body CoM, proximal foot position (derived from a weightless marker attached to the base of the distal tarsometatarsal head, approximate to the end of the heel pad), and distal foot position (taken from a second weightless marker attached to the contact between the final phalange and the ungual of digit III). All parameters were taken in the parasagittal plane.

Because the pes and CoM move relative to one another across stance, we sought to establish their relative positions across each cycle, so we could determine when the pes was likely to be positioned vertically beneath CoM. To do this, we subtracted the proximal foot position from both the CoM and distal foot position, so that the heel serves as a reference point for expressing the position of the CoM versus the foot length. We then subtracted this relative CoM value from the relative distal pes value to generate an alignment score between the CoM the Pes; a value of 0 indicates the CoM is positioned directly above the heel, a value of 1 would indicate the CoM was directly above the distal foot, while values within those bounds show the CoM is located somewhere above the pes, and imply vertical support of the CoM is taking place. Likewise, values below 0 and above 1 indicate a CoM located posteriorly and anteriorly to the pes respectively.

Extended Results

As expected, we found that all breeds underwent a period during the stance phase when the CoM was directly (vertically) above the foot (Supplementary Figure 27). In the mallard and Aylesbury, maximal support occurs around mid-stance, with the CoM passing over the central pes roughly at this time point (Supplementary Figure 27A-B, D). In the Indian runner, this occurs earlier in stance with the CoM (on average) passing the central pes around 40% stance, and having surpassed the distal pes prior to mid-stance (Supplementary Figure 27C-D).

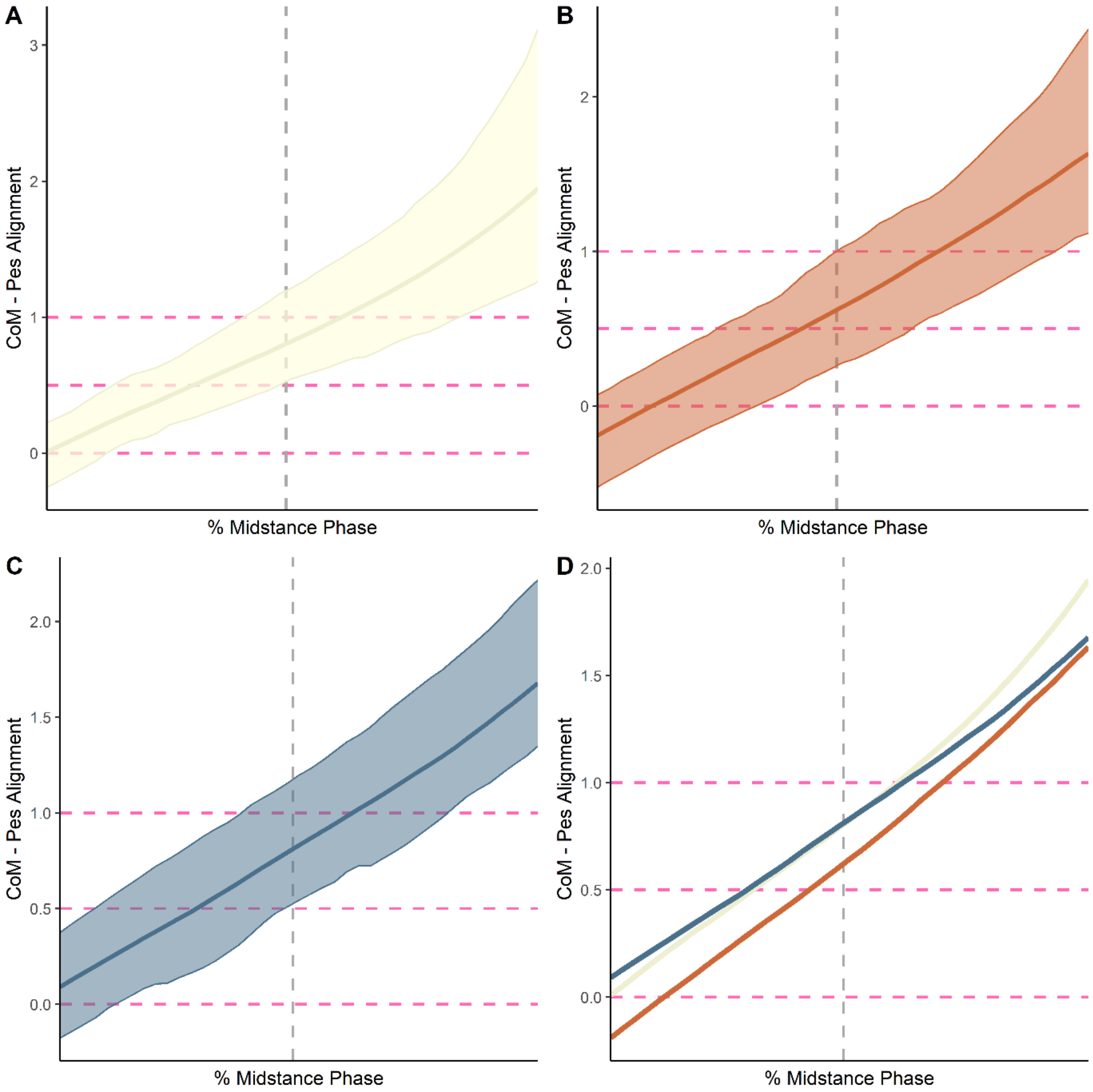

Supplementary Figure 27: Alignment of the whole-body CoM and the pes across the stance phase. For all subplots, the vertical grey lines indicate mid-stance (50% stance), while the horizontal pink lines indicate alignment scores of 0, 0.5 and 1, which indicate periods where the pes would be capable of contributing vertical support of the CoM (see text). The subplots are as follows; **A**, mallard; **B**, Aylesbury duck; **C**, Indian runner; and **D**, all breeds combined. **A**-**C** detail the mean and range from all analysed gait cycles, and include values from the entire stride duration, while **D** comprises only the mean for each breed. All plots are cropped to a ‘midstance phase’ defined as 30-70% of the entire stance phase.

Supplementary Notes 8: Influence of trunk pitching on effective limb length

Extended Methods

Statistical parametric mapping and discrete stride parameter analysis (main text Figure 5; Supplementary Notes 6), have shown that the three breeds utilise very different trunk orientations during walking, where the Mallard and Aylesbury duck remain fairly orthograde, while the Indian runner uses a more pronograde (= upright) posture. Considering difference in body shape (main text Figure 2), and similarity in postural index (PI; main text Figure 6), this finding is (at first glance) surprising, because a cranial CoM should effectively lower the PI since it requires relatively anterior placement of the pes and more flexed postures. Pronounced trunk pitching in the Indian runners may provide the answer to this issue, since pitching would rotate the CoM towards the acetabulum, allowing more extended postures to be used that yield similar PI values to the other breeds.

Here, we have used linear regression to investigate the relationship between midstance trunk pitch and two dependent variables: postural index (= PI) and hip angle. While PI is the primary variable of interest, hip angle provides a secondary comparison because hip flexion plays an important role in setting overall posture in bipeds, because rotation at this joint is responsible for moving the knee cranial to the CoM so the knee extensors may counter the flexor moment at the knee around midstance. Midstance was chosen as an easily definable stage that should approximate the height of vertical support during the stance phase (see Supplementary Notes 7, above). In addition, four variants of each model were run; all breeds combined, and each breed individually.

Extended Results

No significant relationship was found between trunk pitch and PI when investigated across all breeds, or Mallards and Aylesburys in isolation. However, a weak negative correlation was found in Indian runners (Supplementary Figure 28A; Supplementary Table 27). In contrast, midstance trunk pitch and hip angle were found to be negatively correlated across all breeds, and within each breed independently (Supplementary Figure 28B; Supplementary Table 28). The relative effect size varied notably across the breeds, being highest in Indian runners (*adj*. R^2 = 50%), followed by Aylesburys (*adj*. R^2 = 33%), then Mallards (*adj*. R^2 = 21%).

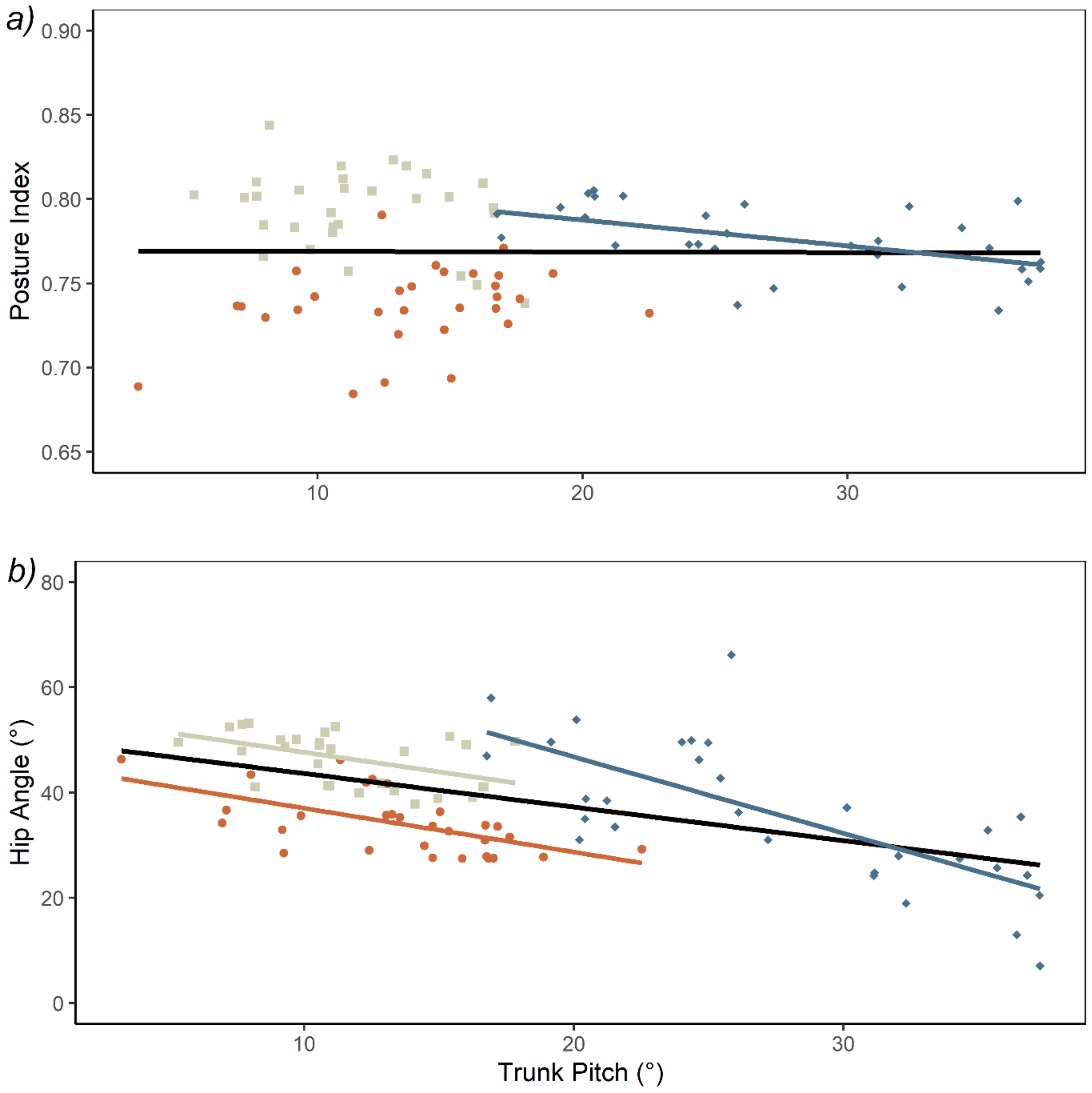

Supplementary Figure 28: Linear regression results of midstance trunk pitch against (**A**) postural index, and (**B**) midstance hip angle. Breeds follow the common colour and point scheme used in other figures. The 'All breeds combined' model is presented on both plots (black line), while breed-specific models are only presented where significant.

Model	F	DF	*p*	R^2	*adj.* R^2
All breeds	0.007	88	0.935	<0.001	-0.011
Mallards only	2.081	28	0.16	0.069	0.036
Aylesburys only	2.531	28	0.123	0.083	0.05
Indian Runners only	10	28	0.004	0.263	0.237

Supplementary Table 27: Model results for linear regression of midstance trunk pitch angle against midstance relative hip height (effective limb length).

Model	F	DF	*p*	R^2	*adj.* R^2
All breeds	33.77	88	<0.001	0.277	0.269
Mallards only	8.509	28	0.007	0.233	0.206
Aylesburys only	15.14	28	0.001	0.351	0.328
Indian Runners only	30.09	28	<0.001	0.518	0.501

Supplementary Table 28: Model results for linear regression of midstance hip angle against midstance relative hip height (effective limb length).

Additional Supplementary Figures

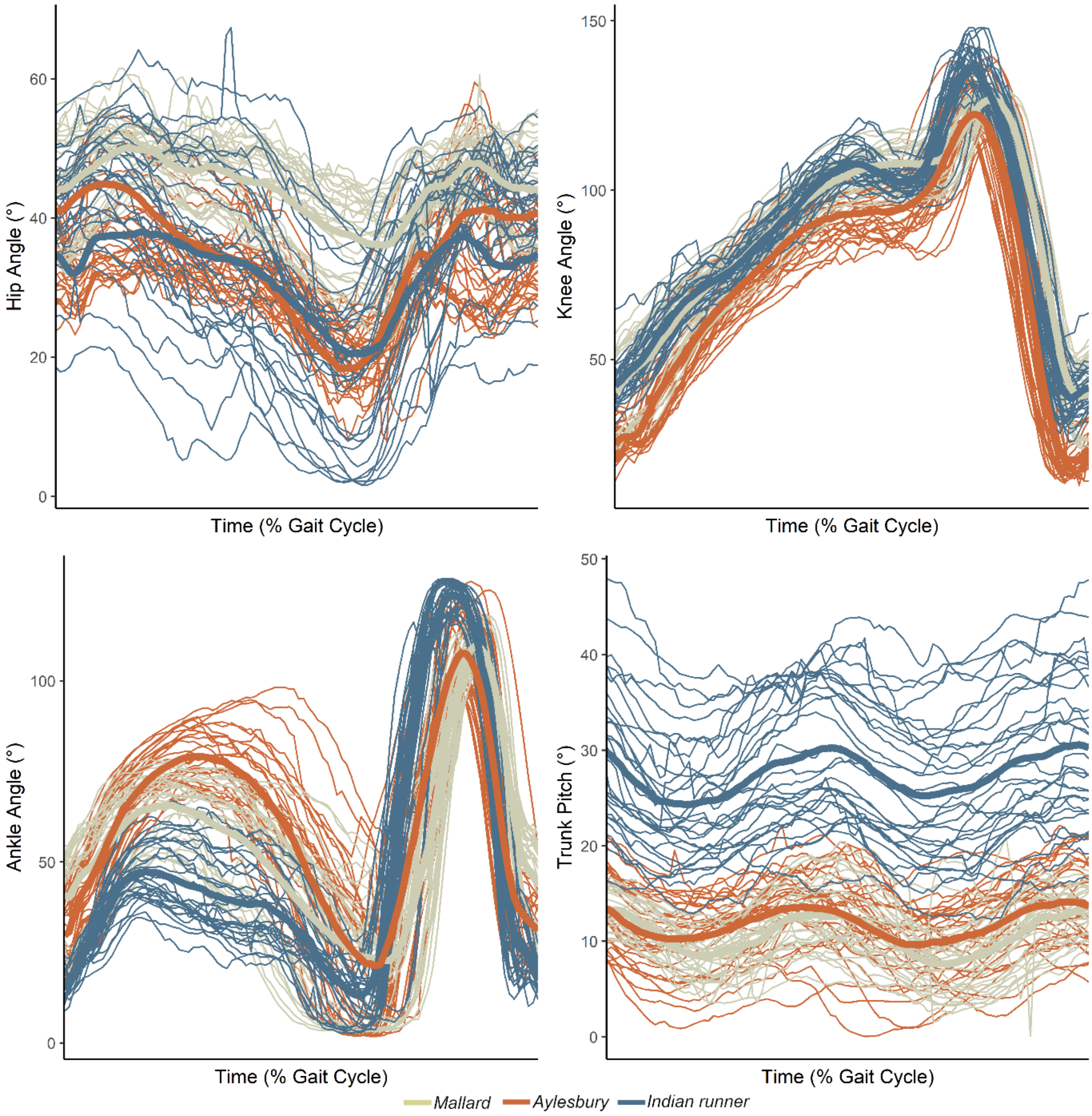

Supplementary Figure 29: All joint and trunk kinematic traces (thin lines) used in the statistical analyses, plotted against the breed-specific mean trace (thick lines). In total, there are 30 traces per breed derived from 3 mallards (10 traces each), 3 Indian runners (10 traces each), and 2 Aylesbury ducks (15 traces each).

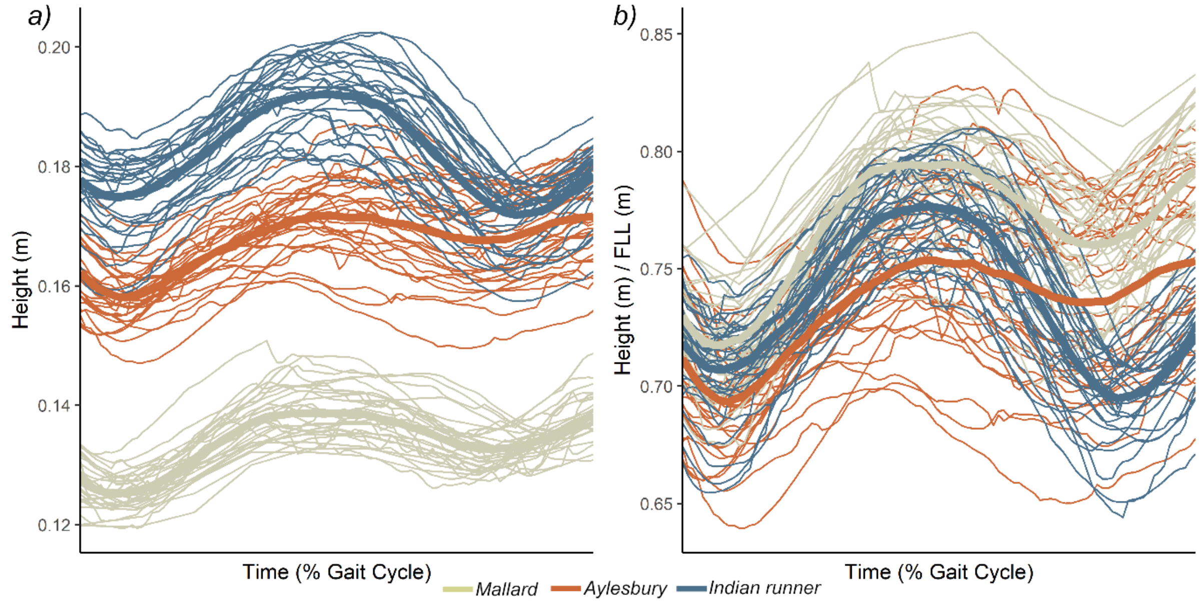

Supplementary Figure 30: All kinematic traces (thin lines) for effective limb length (*a*) and posture index (*b*) used in the statistic parametric mapping analysis, plotted against the breed-specific mean trace (thick lines). In total, there are 30 traces per breed derived from 3 mallards (10 traces each), 3 Indian runners (10 traces each), and 2 Aylesbury ducks (15 traces each).

REFERENCES FOR THE SUPPLEMENTARY MATERIAL

1. Kassambara, A. Rstatix: pipe-friendly framework for basic statistical tests. v. 0.7.0. (2020).
2. Abourachid, A. Kinematic parameters of terrestrial locomotion in cursorial (ratites), swimming (ducks), and striding birds (quail and guineafowl). *Comp. Biochem. Physiol. A.* **131**, 113-119, (2001).
3. Usherwood, J. R., Szymanek, K. L. & Daley, M. A. Compass gait mechanics account for top walking speeds in ducks and humans. *J. Exp. Biol*. **211**, 3744-3749, (2008).
4. Pinshow, B., Fedak, M. A. & Schmidt-Nielsen, K. Terrestrial locomotion in penguins: it costs more to waddle. *Science* **195**, 592-594, (1977).
5. White, C. R., Martin, G. R. & Butler, P. J. Pedestrian locomotion energetics and gait characteristics of a diving bird, the great cormorant, *Phalacrocorax carbo*. *J. Comp. Physiol. B.* **178**, 745-754, (2008).
6. Tickle, P. G., Hutchinson, J. R. & Codd, J. R. Energy allocation and behaviour in the growing broiler chicken. *Sci. Rep*. **8**, 4562, (2018).
7. Paxton, H., Daley, M. A., Corr, S. A. & Hutchinson, J. R. The gait dynamics of the modern broiler chicken: a cautionary tale of selective breeding. *J. Exp. Biol*. **216**, 3237-3248, (2013).
8. Rubenson, J., Heliams, D. B., Maloney, S. K., Withers, P. C., Lloyd, D. G. & Fournier, P. A. Reappraisal of the comparative cost of human locomotion using gait-specific allometric analyses. *J. Exp. Biol*. **210**, 3513-3524, (2007).
9. Jetz, W., Thomas, G. H., Joy, J. B., Hartmann, K. & Mooers, A. O. The global diversity of birds in space and time. *Nature*, **491**, 444-448, (2012).
10. Watson, R. R., Rubenson, J., Coder, L., Hoyt, D. F., Propert, M. W. & Marsh, R. L. Gait-specific energetics contributes to economical walking and running in emus and ostriches. *Proc. R. Soc. B.* **278**, 2040-2046, (2011).
11. Nudds, R. L., Gardiner, J. D., Tickle, P. G. & Codd, J. R. Energetics and kinematics of walking in the barnacle goose (*Branta leucopsis*). *Comp. Biochem. Physiol. A Mol. Integr. Physiol*. **156**, 318-324, (2010).
12. Marsh, R. L., Ellerby, D. J., Carr, J. A., Henry, H. T. & Buchanan, C. I. Partitioning the energetics of walking and running: swinging the limbs is expensive. *Science* **303**, 80-83, (2004).

13. Rose, K. A., Nudds, R. L. & Codd, J. R. Intraspecific scaling of the minimum metabolic cost of transport in leghorn chickens (*Gallus gallus domesticus*): links with limb kinematics, morphometrics and posture. *J. Exp. Biol*. **218**, 1028-1034, (2015).
14. Nudds, R. L., Folkow, L. P., Lees, J. J., Tickle, P. G., Stokkan, K-A. & Codd, J. R. Evidence for energy savings from aerial running in the Svalbard rock ptarmigan (*Lagopus muta hyperborea*). *Proc. Roy. Soc. B*. **278**, 2654-2661, (2011).
15. Wilkinson, H., Thavarajah, N. & Codd, J. R. The metabolic cost of walking on an incline in the Peacock (*Pavo cristatus*). *PeerJ* **3**, e987, (2015).
16. Griffin, T. M. & Kram, R. Penguin waddling is not wasteful. *Nature*. **408**, 929p, (2000).
