# Supplementary material for: Testing the form-function paradigm: body shape correlates with kinematics but not energetics in selectively-bred birds
Source: Commun Biol. 2024 Jul 24;7:900. doi: 10.1038/s42003-024-06592-w (PMC11269648; doi:10.1038/s42003-024-06592-w)
Supplement: Supplementary file 2 — Description of Additional Supplementary Materials [file 42003_2024_6592_MOESM2_ESM.pdf]

Description of Additional Supplementary Files

File name: Supplementary Data 1

Description: Contains all the numerical data needed to repeat the analyses in the main text.
