# Supplementary material for: Testing the form-function paradigm: body shape correlates with kinematics but not energetics in selectively-bred birds
Source: Commun Biol. 2024 Jul 24;7:900. doi: 10.1038/s42003-024-06592-w (PMC11269648; doi:10.1038/s42003-024-06592-w)
Supplement: Supplementary file 4 — Reporting Summary [file 42003_2024_6592_MOESM4_ESM.pdf]

nature portfolio

Corresponding author(s): Samuel Cross

Last updated by author(s): Jul 3, 2024

Reporting Summary

Nature Portfolio wishes to improve the reproducibility of the work that we publish. This form provides structure for consistency and transparency in reporting. For further information on Nature Portfolio policies, see our Editorial Policies and the Editorial Policy Checklist.

Statistics

For all statistical analyses, confirm that the following items are present in the figure legend, table legend, main text, or Methods section.

n/a	Confirmed	
☐	☒	The exact sample size (n) for each experimental group/condition, given as a discrete number and unit of measurement
☐	☒	A statement on whether measurements were taken from distinct samples or whether the same sample was measured repeatedly
☐	☒	The statistical test(s) used AND whether they are one- or two-sided<br>*Only common tests should be described solely by name; describe more complex techniques in the Methods section.*
☐	☒	A description of all covariates tested
☐	☒	A description of any assumptions or corrections, such as tests of normality and adjustment for multiple comparisons
☐	☒	A full description of the statistical parameters including central tendency (e.g. means) or other basic estimates (e.g. regression coefficient) AND variation (e.g. standard deviation) or associated estimates of uncertainty (e.g. confidence intervals)
☐	☒	For null hypothesis testing, the test statistic (e.g. F, t, r) with confidence intervals, effect sizes, degrees of freedom and P value noted<br>*Give P values as exact values whenever suitable.*
☒	☐	For Bayesian analysis, information on the choice of priors and Markov chain Monte Carlo settings
☒	☐	For hierarchical and complex designs, identification of the appropriate level for tests and full reporting of outcomes
☐	☒	Estimates of effect sizes (e.g. Cohen's d, Pearson's r), indicating how they were calculated

Our web collection on statistics for biologists contains articles on many of the points above.

Software and code

Policy information about availability of computer code

Data collection	Tracker v.4.97 was used to collect spatiotemporal kinematic data. Qualisys v.2.15 was used to collect 3D marker data. OpenSIM v.4.3 was used to generate joint kinematic data from the marker data. Mimics v.23.0 was used for segmentation of the CT scan data and Blender v.2.90.1 was used for reposing and measuring the 3D models.
Data analysis	R v.3.6.6 (implemented in R Studio v.4.0.5.) and Matlab v. R2022a, were used for data processing and formal analysis with standard/published functions which are cited in the main text.

For manuscripts utilizing custom algorithms or software that are central to the research but not yet described in published literature, software must be made available to editors and reviewers. We strongly encourage code deposition in a community repository (e.g. GitHub). See the Nature Portfolio guidelines for submitting code & software for further information.

Data

Policy information about availability of data

All manuscripts must include a data availability statement. This statement should provide the following information, where applicable:

- Accession codes, unique identifiers, or web links for publicly available datasets
- A description of any restrictions on data availability
- For clinical datasets or third party data, please ensure that the statement adheres to our policy

The numerical data needed to repeat the analyses are available in Supplementary Dataset 1. The 3D morphological and kinematic data are made available via the

University of Liverpool Research Data Catalogue and can be accessed via the following link https://doi.org/10.17638/datacat.liverpool.ac.uk/2734. Also accessible via that link is the code used to run the analyses, and this has been formatted with the corresponding numerical data (as provided in Supplementary Dataset 1) for ease of use.

Research involving human participants, their data, or biological material

Policy information about studies with human participants or human data. See also policy information about sex, gender (identity/presentation), and sexual orientation and race, ethnicity and racism.

Reporting on sex and gender	N/A
Reporting on race, ethnicity, or other socially relevant groupings	N/A
Population characteristics	N/A
Recruitment	N/A
Ethics oversight	N/A

Note that full information on the approval of the study protocol must also be provided in the manuscript.

Field-specific reporting

Please select the one below that is the best fit for your research. If you are not sure, read the appropriate sections before making your selection.

☐ Life sciences ☐ Behavioural & social sciences ☒ Ecological, evolutionary & environmental sciences

For a reference copy of the document with all sections, see nature.com/documents/nr-reporting-summary-flat.pdf

Ecological, evolutionary & environmental sciences study design

All studies must disclose on these points even when the disclosure is negative.

Study description	We collected locomotor energetic and kinematic data from three breeds of ducks (22 total) during treadmill trials at different walking speeds, and then collected morphometric data from CT scans of the euthanised birds. Data was analysed using standard analytical procedures for each of the three categories investigated (morphology, energetics, and kinematics). Morphological analysis involved statistical testing (ANOVA, PCA) of segment parameter differences between the breeds (36 parameters total), as well as a linear regression of leg and neck length, and CoM estimation for 1 bird from each breed. Kinematic analysis comprised ANCOVA of the spatiotemporal kinematics (90 trials total), as well as statistic parametric mapping of the joint and trunk kinematics, raw and normalised hip height, and CoM versus pes position in 8 birds total across the 3 breeds, across the same 90 strides. ANCOVA was also used to analyse breed-specific differences in the energetics of locomotion (104 trials total). Minimum cost of transport in each breed was compared against equivalent data in other birds, which was sourced from the literature.
Research sample	22 male, adult (>8 month), mallards (Anas platyrhynchos). These ducks were divided into 3 breeds, 'wild-type' mallards (n=8), Indian runner ducks (n=8), and Aylesbury ducks (n=8). These breeds were chosen because they diverge significantly in overall body shape, whilst being conspecific - this aligns directly with our research questions.
Sampling strategy	A sample size of 5-10 birds per category is standard for experimental studies of this type and is considered suitable for achieving the required statistical power. Thus, our sampling of 8/8/6 for each of the three breeds (22 total) is within this range.
Data collection	For the experimental data, the birds were exercised on a treadmill at different speeds and filmed laterally. Energetic data was acquired using open flow respirometry, while spatiotemporal kinematic data was tracked based on the position of the nearest (right) foot. For several birds of each breed, markers were attached to the trunk and limb joints and the trial repeated. This marker data was used to drive inverse kinematics of a 3D model in OpenSim, from which joint kinematic data could be calculated. The morphological data was collected post mortem from CT scans of each bird in Mimics. Authors ACM-G, KTB, JCM, KAR & JRC collected the experimental data, while SRRC & KTB collected the morphological and joint kinematic data. Data was tabulated into .xls format prior to analysis.
Timing and spatial scale	Experimental trials were undertaken between 30/05/17 and 25/11/17, during which one bird was sampled randomly approximately every day. This low rate of sampling ensured the birds did not become fatigued and were well rested prior to their follow up experiment.
Data exclusions	Numerical outliers were identified through statistical methods (QQ-plots, Grubbs Test) and statistical analyses were run with and without them to assess any qualitative influence upon the overall conclusions.
Reproducibility	The experimental trials are identical for all birds/breeds (treadmill locomotion at different speeds) and multiple trials were performed for each bird/breed, which we take as evidence for its reproducibility. In some instances a bird may refuse to participate

	in a trial on any given day, leading to a failed trial. This was a sporadic event and the bird would be removed to rest and tested later at another time.
Randomization	Birds/breeds were randomized for the experimental trials.
Blinding	Blinding was not possible (or deemed necessary) for these experiments. The grouping category (breed), and in most cases each individual bird, are visually distinct and would be impossible to hide from those collecting the data.

Did the study involve field work? ☐ Yes ☒ No

Reporting for specific materials, systems and methods

We require information from authors about some types of materials, experimental systems and methods used in many studies. Here, indicate whether each material, system or method listed is relevant to your study. If you are not sure if a list item applies to your research, read the appropriate section before selecting a response.

Materials & experimental systems

n/a	Involved in the study
☒	☐ Antibodies
☒	☐ Eukaryotic cell lines
☒	☐ Palaeontology and archaeology
☐	☒ Animals and other organisms
☒	☐ Clinical data
☒	☐ Dual use research of concern
☒	☐ Plants

Methods

n/a	Involved in the study
☒	☐ ChIP-seq
☒	☐ Flow cytometry
☒	☐ MRI-based neuroimaging

Animals and other research organisms

Policy information about studies involving animals; ARRIVE guidelines recommended for reporting animal research, and Sex and Gender in Research

Laboratory animals	22 Mallards (Anas platyrhynchos); composed of 8 wild-type (described as 'mallards' within the main text), 8 Indian runner ducks (domesticated breed), and 6 Aylesbury ducks (domesticated breed). The birds were mature adults, greater than 8 months of age.
Wild animals	The study did not involve wild animals.
Reporting on sex	All animals in the study were male, and sex was not considered in the study design. Sex was determined by the breeders using standard procedure for the species (plumage characteristics).
Field-collected samples	The study did not involve samples collected from the field.
Ethics oversight	University of Manchester Ethics Committee

Note that full information on the approval of the study protocol must also be provided in the manuscript.

Plants

Seed stocks	NA
Novel plant genotypes	NA
Authentication	NA
